# Upcycling polyolefins to methane-free liquid fuel by a Ru_1_-ZrO_2_ catalyst

**DOI:** 10.1038/s41467-025-57998-x

**Published:** 2025-03-21

**Authors:** Jicong Yan, Guanna Li, Zhanwu Lei, Xiaolu Yuan, Junting Li, Xiaoru Wang, Bo Wang, Fuping Tian, Tao Hu, Lei Huang, Yujia Ding, Xiaoke Xi, Feng Zhu, Shuo Zhang, Jiong Li, Yu Chen, Ruiguo Cao, Xiang Wang

**Affiliations:** 1 State Key Laboratory of Fine Chemicals, School of Chemical Engineering, Dalian University of Technology, Dalian, Liaoning China; 2 Biobased Chemistry and Technology, Wageningen University, WG Wageningen, the Netherlands; 3 Department of Materials Science and Engineering, University of Science and Technology of China, Hefei, Anhui China; 4 School of Chemistry, Dalian University of Technology, Dalian, Liaoning China; 5 Research Center of Nano Science and Technology, College of Sciences, Shanghai University, Shanghai, China; 6 Department of Physics and CSRRI, Illinois Institute of Technology, Chicago, IL USA; 7 TRACE EM Unit and Department of Materials Science and Engineering, City University of Hong Kong, Kowloon, Hong Kong SAR China; 8 Shanghai Synchrotron Radiation Facility, Shanghai Advanced Research Institute, Chinese Academy of Sciences, Shanghai, China

**Keywords:** Heterogeneous catalysis, Catalytic mechanisms, Catalyst synthesis

## Abstract

Upcycling waste plastics into liquid fuels presents significant potential for advancing the circular economy but is hindered by poor selectivity and low-value methane byproduct formation. In this work, we report that atomic Ru-doped ZrO_2_ can selectively convert 100 grams of post-consumer polyethylene and polypropylene, yielding 85 mL of liquid in a solvent-free hydrocracking. The liquid (C_5_-C_20_) comprises ~70% jet-fuel-ranged branched hydrocarbons (C_8_-C_16_), while the gas product is liquefied-petroleum-gas (C_3_-C_6_) without methane and ethane. We found that the atomic Ru dopant in the Ru-O-Zr moiety functionalizes its neighboring O atom, originally inert, to create a Brønsted acid site. This Brønsted acid site, rather than the atomic Ru dopant itself, selectively governs the internal C−C bond cleavage in polyolefins through a carbonium ion mechanism, thereby enhancing the yield of jet-fuel-ranged hydrocarbons and suppressing methane formation. This oxide modulation strategy provides a paradigm shift in catalyst design for hydrocracking waste plastics and holds potential for a broad spectrum of applications.

## Abstract (web summary)

Converting waste plastics into liquid fuels holds great promise for advancing the circular economy but is challenged by poor selectivity and unwanted methane byproduct formation. Here, the authors present a Ru_1_-ZrO_2_ catalyst that efficiently upcycles plastic waste into high-value jet fuel–range hydrocarbons with minimal methane production.

## Introduction

Polyolefins (PO), comprising two-thirds of total plastic production^1^, are the most widely used plastics due to their inherent durability and resistance to degradation. However, these traits also contribute to the persistence of PO waste in the environment, causing severe environmental issues and a loss of 95% economic value of plastic^2^. PO waste, primarily sourced from fossil feedstock, represents a large reservoir of carbon and hydrogen, offering great opportunities for a circular economy through upcycling processes^3–6^. Chemical upcycling can tackle this challenge^7^ by deconstructing PO into valuable chemicals^8,9^ and sustainable fuels^10–18^, thus enhancing the value chain of plastic waste recycling and creating a circular plastic economy.

Converting waste PO to liquid fuels presents a high potential to partially replace petroleum-route fuels and reduce global net carbon emissions. Common chemical upcycling strategies in practical terms include gasification and pyrolysis^14,15^, but these methods are energy-intensive, produce broad product distributions, and yield significant amounts of low-value methane. In contrast, hydrocracking with metal/oxide bifunctional catalysts has shown promise in producing high-value fuels, waxes, and lubricants under milder conditions, usually below 300 °C^16–24^. Nevertheless, achieving high selectivity for liquid fuels, particularly in the jet fuel range (C_8_-C_16_), over metal/oxide catalysts remains challenging.

Among metal/oxide catalysts, Ru-based catalysts have been studied in the hydroconversion of polyolefins due to their outstanding efficiency in breaking C–C bonds^25^. However, its tendency to generate substantial amounts of low-value methane significantly diminishes the economic viability of the hydrocracking process. Several operating strategies have been developed to suppress methane formation, such as decreasing reaction temperature^26^, shortening reaction time, and increasing H_2_ pressure^27,28^. However, these operating optimizations often result in the under-cracking of polyolefins, leading to a broad carbon distribution with low selectivity for jet-fuel-ranged hydrocarbons. To overcome the economic bundle by maximizing jet fuel component yield and minimizing methane production, the fundamental solution still lies in designing an active site capable of selectively breaking the internal C−C bond of PO rather than the terminal C−C bond. Efforts have been focused on the manipulation of supported metal species^17^, and regulation of the oxidation state^29,30^, particle size^21,31–34^ and surface decoration^35^ of these metal species has proved its effectiveness in reducing undesired methane production. For instance, positively charged Ru species have been reported to favor the cleavage of the internal C−C bond over the terminal C−C bond, thus inhibiting methane formation, but the yield of methane is still at 4.5% with a conversion of PE at 80%^36^. Ru nanoparticles with multiple exposed Ru sites tend to cleave the terminal C–C bond producing methane, and the sub-nanometer particle or single atom of Ru was found effective in suppressing methane formation, but a low methane yield was only obtained in the scenario of under-cracking of PO with a wide distribution of the liquid/wax products up to C_40_^31,32^. Decoration of 5 wt% Ru/ZrO_2_ with 25 wt% WO_3_ significantly reduced methane yield from 15% to 5%, but this improvement came at the cost of PO conversion reduced from 75% to 60%^35^. Therefore, the low methane output and the high jet fuel yield still cannot be simultaneously achieved at a high polyolefin conversion, appearing either under-cracking or over-cracking. Still, if the metal site governing the internal C−C bond cleavage is unclear, impeding the rational design of active sites that can selectively promote internal C-C scission while not triggering terminal cascades.

Instead of manipulating the supported metal species, herein, we functionalize the lattice oxygen of oxides via atomic doping to be the active site for selectively cleaving the internal C−C bond of polyolefins. In this proof-of-concept work, we chose ZrO_2_, an originally inert oxide, as the base material, and Ru, a well-known methanation metal typically favoring methane formation during hydrocracking, as the dopant. We found that the Ru dopant turns its neighboring originally inert O atom of the Ru-O-Zr moiety into an active site for selectively cleaving the internal C-C bond. This Ru-doped ZrO_2_ (Ru_1_-ZrO_2_) achieves ideal hydrocracking of 100 g of post-consumer PP and PE mixture in a solvent-free process, yielding 85 mL of liquid fuels with approximately 70% jet-fuel-range hydrocarbons, without forming low-value methane and ethane.

## Results

### Hydrocracking polyolefins

**Fig. 1:**
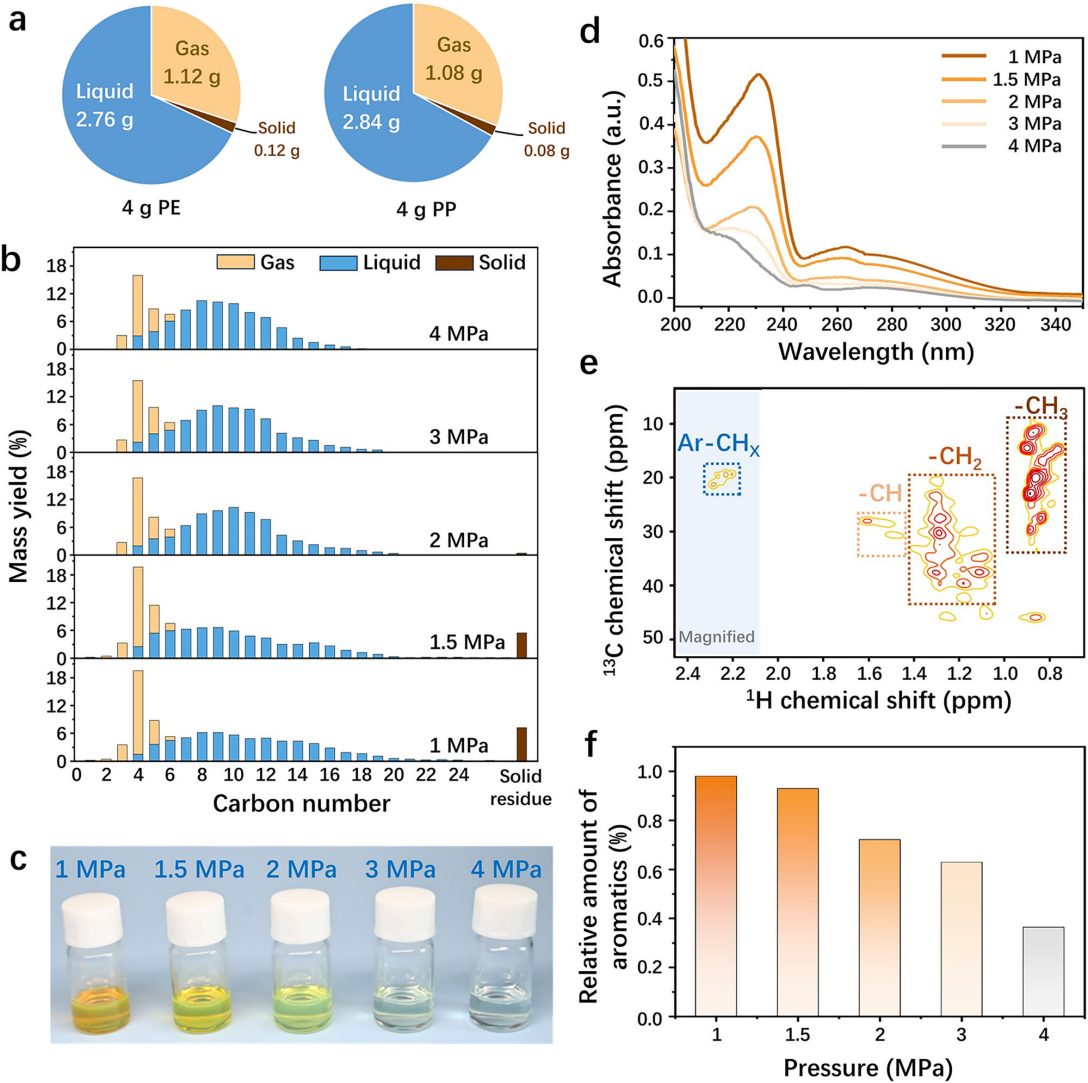
Solvent-free hydrocracking of PE and PP to methane-free fuels. **a** The yields of liquid and gaseous products in solvent-free hydrocracking 4 g PE or PP over Ru_1_-ZrO_2_ at 250 °C under 3 MPa H_2_ for 8 hours. **b–f** Hydrocarbon distribution (**b**), Photo (**c**), UV- Vis absorption spectra (**d**), ^13^C−^1^H HSQC NMR (**e**) and relative amount of aromatics (**f**) calculated based on ^1^H NMR (Supplementary Fig. 8) of the liquid product of hydrocracking PP on Ru_1_-ZrO_2_ at 300 °C under 1.5 MPa H_2_ (**e**) and 1-4 MPa H_2_ (**f**) for 8 hours.

In a typical hydrocracking process involving 4 grams of PE or PP, a conversion of over 98% can be achieved using Ru_1_-ZrO_2_ at 250 °C under 3 MPa H_2_ for 8 hours, yielding approximately 2.8 grams of liquid and 1.1 grams of gas, as illustrated in Fig. 1a. The products are in gas and liquid phases mainly ranging from C_3_ to C_20_, and remain consistent regardless of the substrate as PE or PP (Supplementary Fig. 1). The liquid consists of approximately 70% jet-fuel-range hydrocarbons (C_8_-C_16_), while the gas primarily comprises C_3_-C_6_ LPG-range hydrocarbons (liquified-petroleum-gas, C_3_-C_4_ > 70%), and is almost free of methane and ethane (<0.007% and <0.068% mass yields, respectively, as shown in Supplementary Figs. 1, 2). Notably, for the gaseous C_4_-C_6_ fraction, *iso*-alkanes dominate with a 93% weight fraction (Supplementary Fig. 3), even when the substrate is the less branched PE. This result implies that significant isomerization occurs over Ru_1_-ZrO_2_ during hydrocracking.

High temperatures enhance the thermodynamic driving force toward terminal C−C bond cleavage, leading to increased methane production^26,37^. However, they also shorten the required reaction time, which is often practical in industrial settings. Low H_2_ pressure is also highly desirable in real practice due to the lower equipment requirements, but it reduces competitive hydrogen adsorption on the catalyst resulting in higher methane formation rates^27,28,38^. Prolonged reaction times are often required for cracking the heavy hydrocarbons to get the liquid product, but it increases the likelihood of over-cracking lighter hydrocarbon products to methane. PP, which contains more methyl groups, tends to produce more methane than PE, due to terminal C−C bond cleavage^33,39^. To examine the product preference for jet-fuel-ranged hydrocarbons and resistance to methane formation, a proof test over Ru_1_-ZrO_2_ was conducted under conditions favorable for methane formation, i.e., PP as the substrate at 300 °C under H_2_ of 1–4 MPa for 8 hours (note: 2 hours is sufficient to achieve 98% PP conversion under these conditions, as shown in Supplementary Fig. 4).

As shown in Supplementary Fig. 5, increasing the H_2_ pressure from 1 MPa to 2 MPa enhances the PP conversion from 92% to nearly 100% and increases the liquid yield from 65 wt% to 72 wt%. Further increasing H_2_ pressure from 2 MPa to 4 MPa hardly affects the conversion of PP or the yield of liquid. Nonetheless, higher H_2_ pressure results in greater H_2_ consumption (Supplementary Fig. 6), indicating that more C-C bonds are cleaved, producing short-chain hydrocarbons but they remain in the liquid phase. This is evidenced by the narrower carbon number distribution of the liquid as the H_2_ pressure increases (Fig. 1b). Notably, although the carbon number distribution narrows to C_3_-C_18_ with increased H_2_ pressure, it remains centered around C_8_, leading to an increased selectivity of jet-fuel-ranged hydrocarbons (C_8_-C_16_) up to 77% in the liquid, while maintaining liquid and gas yields at 75 wt% and 25 wt%, respectively. These results suggest that the over-cracking of lighter liquid hydrocarbons is suppressed, while the heavier hydrocarbons larger than C_8_ are selectively deconstructed. As previously reported^26–28,37,38^, the low H_2_ pressure and high reaction temperatures favor the formation of methane and ethane. This trend holds true in this proof test (Supplementary Table 1 lists C_1_-C_2_ values from Fig. 1b). However, Ru_1_-ZrO_2_ still keeps the methane yield below 0.2 wt% while maintaining liquid fuels (C_5_-C_20_) around 70 wt%, even under the reaction condition most greatly favoring methane formation.

The influence of H_2_ pressure on the composition of the liquid product is more pronounced than that on the gas product, as visually indicated by the color change of the liquid from orange to yellow to colorless with the increase of H_2_ pressure from 1 MPa to 4 MPa (Fig. 1c). This observation motivated us to prioritized Ultraviolet-Visible (UV-Vis) absorption spectroscopy characterization for these liquid products. The absorption at 210–320 nm is present for all liquid products (Fig. 1d) but its intensity significantly decreases as the color of the liquid changes from orange to colorless. This adsorption can be attributed to the π-π^*^ transition of aromatics^40^. To further confirm the formation of aromatics, the ^13^C−^1^H HSQC NMR was employed. Besides the correlation peaks assigned to -CH_3_, -CH_2_, and -CH groups (as marked in Fig. 1e), the correlation peaks at (^1^H: 2.0–2.5 ppm, ^13^C: 15–20 ppm) are observed and resulted from CH_x_- directly connected with aromatic rings. The presence of the signals at (^1^H: 6.7–8.0 ppm, ^13^C: 115–135 ppm)^8^ attributed to aromatic rings also confirmed the presence of aromatics in the liquid product (Supplementary Fig. 7). The relative concentration of aromatics in the liquid product decreases as the H_2_ pressure increases (Fig. 1f, plotted based on the calculation from Supplementary Fig. 8), which is consistent with the UV-Vis spectra results, indicating lower H_2_ pressure facilitates the formation of aromatics. These findings not only reaffirm the absence of methane even under reaction conditions favoring terminal C-C bond cleavage but also indicate the occurrence of dehydrogenation and isomerization during the hydrocracking reactions.

### Identification of active site

**Fig. 2:**
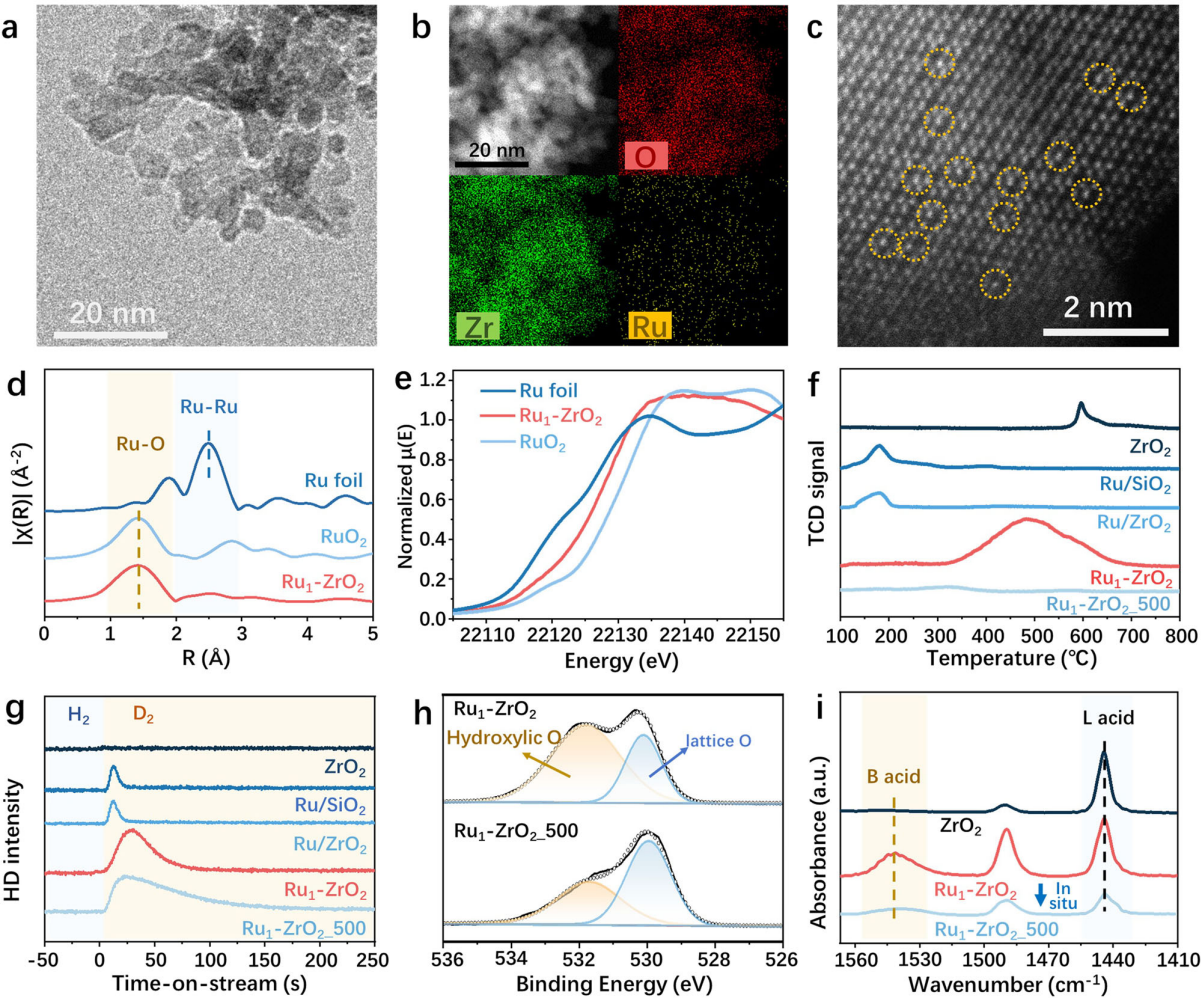
Relationship of Ru-O-Zr structure and performance of Ru_1_-ZrO_2_. **a–e** TEM image (**a**), EDS element mappings (**b**), HAADF-STEM image (**c**), Fourier transform of EXAFS spectra (**d**), and Ru *K*-edge XANES spectra (**e**) of Ru_1_-ZrO_2_. Ru foil and RuO_2_ were used as references. **f** H_2_-TPR profiles of ZrO_2_, Ru/SiO_2_, Ru/ZrO_2_, Ru_1_-ZrO_2_, and Ru_1_-ZrO_2__500. These samples were not pretreated in H_2_ at 275 °C to obtain their complete TPR profiles. **g** H_2_/D_2_ switch experiment tracking m/z = 3 (HD) by mass spectrometer: 10% H_2_/Ar flow switched to 10% D_2_/Ar flow with a constant rate of 20 mL/min at 250 °C at the time of 0 s. **h** O 1 *s* XPS spectra of Ru_1_-ZrO_2_ and Ru_1_-ZrO_2__500. **i** FTIR spectra of pyridine adsorption at room temperature on ZrO_2_ and Ru_1_-ZrO_2_ before and after (Ru_1_-ZrO_2__500) in situ treatment in 20% H_2_/Ar at 500 °C.

This as-prepared Ru_1_-ZrO_2_ with 1.3 wt% Ru loading exhibits a tetragonal phase of ZrO_2_ with no diffraction peaks attributed to Ru in X-ray diffraction (XRD) patterns (Supplementary Fig. 9). The particle size of Ru_1_-ZrO_2_ is around 5 nm (Fig. 2a), and energy-dispersive X-ray spectroscopy (EDS) element mappings confirm that the absence of Ru clusters in the catalyst (Fig. 2b). The HAADF-STEM image shows bright atoms randomly doped in the lattice of ZrO_2_ (Fig. 2c), presumed to be Ru. Fourier transform extended X-ray absorption fine structure (EXAFS) spectra display a major peak corresponding to the Ru-O scattering path at ~1.5 Å in the first coordination sphere (Fig. 2d). The first-shell Ru-O coordination number (CN) is determined to be four based on EXAFS fitting (Supplementary Table 2 and Supplementary Fig. 10). These results confirm that the Ru species in Ru_1_-ZrO_2_ is atomic Ru coordinated with four O atoms in the ZrO_2_ lattice. This is further supported by X-ray absorption near edge structure (XANES) and X-ray photoelectron spectroscopy (XPS) results (Fig. 2e and Supplementary Fig. 11), indicating that the Ru species in Ru_1_-ZrO_2_ are in an oxidized state rather than a metallic state.

Temperature-programmed reduction with H_2_ (H_2_-TPR) was conducted to understand the impact of the interaction between ZrO_2_ and Ru on the reduction of the Ru and ZrO_2_ components in the catalyst. Supported 1.5 wt% Ru/ZrO_2_, 1.6 wt% Ru/SiO_2_ and as-prepared bare ZrO_2_ were used as references. To obtain the full TPR profiles in the range of 100–800 °C, none of these samples were pretreated in H_2_, ensuring that Ru remained in the oxidized state at the start of the H_2_-TPR measurement. A peak at 180 °C is observed for supported Ru/SiO_2_ and Ru/ZrO_2_ (Fig. 2f) and attributed to the reduction of RuO_2_ clusters^39^ to metallic Ru (confirmed by HRTEM in Supplementary Fig. 12). For as-prepared bare ZrO_2_, a peak around 600 °C is observed and attributed to the reduction of ZrO_2_^41^. Unlike these two peaks, a significant peak around 500 °C is observed for Ru_1_-ZrO_2_. The peak is much closer in temperature to the ZrO_2_ reduction peak than to the RuO_2_ reduction peak, but its integration intensity is much higher than that of ZrO_2_ reduction peak. Quantitative analysis of these peaks indicates that the amount of H_2_ consumed by Ru_1_-ZrO_2_ is around 12 times higher than that consumed by supported Ru/SiO_2_, Ru/ZrO_2_, and ZrO_2_ (Supplementary Fig. 13). This result confirms the observation of HRTEM and EXAFS that no Ru clusters or nanoparticles are present in Ru_1_-ZrO_2_, and further indicates that the atomic Ru dopants render more O atoms in Ru_1_-ZrO_2_ reducible compared to as-prepared bare ZrO_2_. Therefore, it is confirmed that all Ru atoms were doped into the crystal of ZrO_2_, forming the moieties of Ru-O-Zr, where the O atoms were activated due to the doping of Ru.

None of the supported Ru/SiO_2_, Ru/ZrO_2_, and bare ZrO_2_ references show activity in the hydrocracking of PP (Supplementary Fig. 14). The significant difference between the active Ru_1_-ZrO_2_ and the inert references, as reflected in TPR profiles, is the prominent peak at 500 °C. We speculated that this peak-related structure, i.e., Ru-O-Zr, is relevant to the catalytic performance of Ru_1_-ZrO_2_. To verify this correlation, the Ru-O-Zr species were intentionally demolished by treating the Ru_1_-ZrO_2_ at 500 °C in a 20% H_2_/Ar flow of 50 mL/min for 0.5 hours (denoted as Ru_1_-ZrO_2__500). We found that in Ru_1_-ZrO_2__500, the disappearance of the Ru-O-Zr reduction signal in the H_2_-TPR profile (Fig. 2f) is accompanied by the loss of activity in PP hydrocracking, as demonstrated by the weight-balanced post-reaction solid residue (Supplementary Fig. 15) and the unchanged pressure during the reaction (Supplementary Fig. 16).

We further pursued which atomic site within the Ru-O-Zr moiety governs the internal C-C bond cleavage, so the changes in the Ru_1_-ZrO_2_ after H_2_ treatment at 500 °C were studied. It was found that Ru_1_-ZrO_2__500 maintains the tetragonal phase of ZrO_2_ (XRD in Supplementary Fig. 17), and Ru remains atomic dopant within the ZrO_2_ crystal, as depicted by HRTEM (Supplementary Fig. 18) and EXAFS (Supplementary Fig. 19). However, XANES (Supplementary Fig. 20) and XPS (Supplementary Fig. 21) indicate that the oxidation states of both Ru and Zr shift toward their reduced states after H_2_ treatment. Inductively coupled plasma-optical emission spectroscopy (ICP-OES) and XPS analyses suggest no loss of Ru after this H_2_ treatment (Supplementary Table 3). The capability of hydrogenation/dehydrogenation involving H_2_ dissociation of the catalysts was further examined using an H_2_/D_2_ switch experiment. As shown in Fig. 2g, the integrated intensity of the HD peak on Ru_1_-ZrO_2__500 is slightly higher than that on Ru_1_-ZrO_2_, but both of them are more than 8 times higher than that on supported Ru/SiO_2_ and Ru/ZrO_2_. This observation demonstrates that the hydrogenation/dehydrogenation capability of Ru_1_-ZrO_2__500 is enhanced or at least maintained compared to Ru_1_-ZrO_2_. During this H_2_ treatment, the significant change in the Ru-O-Zr moiety is the removal of its O atom, as evidenced by the formation of H_2_O (H_2_-TPR-MS in Supplementary Fig. 22) and the decreased intensity of hydroxylic O in O 1 *s* XPS spectra^42,43^ (Fig. 2h). Therefore, we conclude that within the Ru-O-Zr moiety, it is the O atom, rather than Ru, that is the key site governing C-C bond cleavage in polyolefin hydrocracking.

Given the observation of isomerization during the hydrocracking PO, the acid sites were further studied. Pyridine-FTIR spectra (Fig. 2i) show only Lewis acid sites with a feature peak at 1444 cm^−1^ on the inert ZrO_2_^16^. Upon doping with atomic Ru, another peak at 1540 cm^−1^, attributed to Brønsted acid sites^16^, is observed on Ru_1_-ZrO_2_. After in situ treatment of Ru_1_-ZrO_2_ at 500 °C in 20% H_2_/Ar (equivalent to Ru_1_-ZrO_2__500), a significant decrease in the intensities of the FTIR peaks is observed, particularly for the peak attributed to Brønsted acid sites, indicating a dramatic decrease in the amount of acid sites. This result is further confirmed by the temperature-programmed NH_3_ desorption (NH_3_-TPD). The integrated intensity of NH_3_ desorption peak for Ru_1_-ZrO_2_ is 11 times higher than that for Ru_1_-ZrO_2__500 (Supplementary Fig. 23). Moreover, the temperature for the complete NH_3_ desorption is observed up to 450 °C on Ru_1_-ZrO_2_, while it is only around 260 °C for Ru_1_-ZrO_2__500. These results reveal a dramatic decrease in both the population and strength of the acid sites on Ru_1_-ZrO_2_ after H_2_ treatment at 500 °C. Given that both the activated atom after atomic Ru doping and the removed atom after the H_2_ treatment at 500 °C are the O atom of the Ru-O-Zr moiety, it is concluded that the O atom of the Ru-O-Zr moiety has a dangling H, which acts as the Brønsted acid site.

We further performed charge and chemical bonding analysis for Ru-O-Zr moiety with a dangling H on the Ru-doped ZrO_2_. Crystal Orbital Hamilton Population (COHP) analysis (Supplementary Fig. 24) revealed that, compared to ZrOHZr (assuming it exists), the bonding and antibonding O-H interactions in RuOHZr shifted to higher energy levels. This indicates that the O-H interaction in RuOHZr is relatively weaker, suggesting higher acidity of the OH group. Moreover, the integrated COHP values (Supplementary Table 5) show that the O-H bond distance in the RuOHZr moiety is longer than ZrOHZr and is closer to the Fermi level, further suggesting a weaker O-H interaction, i.e. a stonger acidity, in RuOHZr, which is in line with the results of NH_3_-TPD.

### Mechanism of selective internal C-C bond cleavage

We performed the hydrocracking of *n*-octane, aiming to get a preliminary understanding of the way of PO hydrocracking on Ru_1_-ZrO_2_. The products from *n*-octane are also free of methane and ethane, predominated by *iso*-butane with a selectivity of 60% in the gas products (Supplementary Fig. 25a). This product distribution indicates that the isomerization followed by β-scission of the carbonium ion regime is the primary pathway in the hydrocracking of *n*-octane (details in Supplementary Fig. 25b). The methane-free branched products from the hydrocracking of longer-chain hydrocarbons (Supplementary Fig. 26), such as *n*-dodecane (C_12_) and squalane (C_30_), further verified this conclusion. With this understanding, we conducted density functional theory (DFT) calculations to explore why the products are free of methane and dominated by isomerized hydrocarbons during PP hydrocracking over Ru_1_-ZrO_2_.

**Fig. 3:**
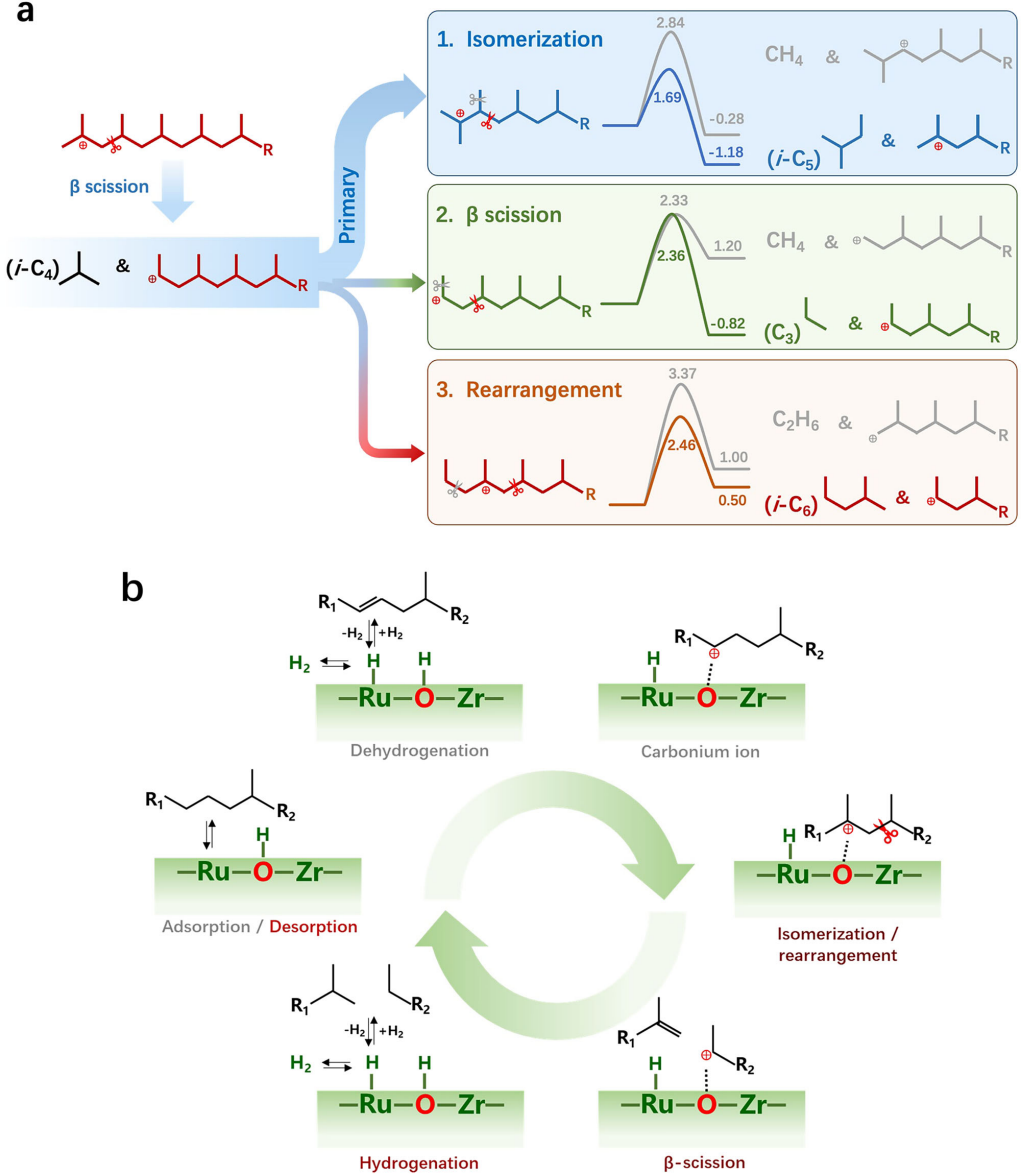
β-scission mechanism of PO hydrocracking over Ru_1_-ZrO_2_. **a** Reaction pathways and DFT calculated energy profiles of PP hydrocracking to gas products via β-scission mechanism with carbonium ions (energy unit: eV). The molecule structures in red or blue color are the same type of carbonium circularizing in the hydrocracking loop. **b** Schematic catalytic cycle of PO (PE or PP) hydrocracking on Ru_1_-ZrO_2_.

During PP deconstruction, the formation of the more stable tertiary carbonium ion is favored over, primary and secondary carbonium ions^44–46^. For the production of lighter hydrocarbons, the tertiary carbonium ion forms at the terminal of the carbon chain either initially or as a result of precedent β-scission of an internal C-C bond (Fig. 3a). The β-scission triggered by the terminal tertiary carbonium ion (scissors-marked molecule in the left panel), forms one *i*-C_4_ molecule and a long-chain hydrocarbon with a secondary carbonium ion. Subsequent depolymerization from the resulting secondary carbonium ion of the residue long-chain hydrocarbon can proceed through three options: 1) isomerization (blue route), 2) direct β-scission (green route), or 3) rearrangement to form a tertiary carbonium ion (orange route). Regarding route 1), after isomerization there are two plausible C-C bond cleavage sites (marked with grey and red scissors) via β-scission, forming methane and *i*-C_5_, respectively. DFT calculations indicate that the reaction forming methane is energetically less favorable (∆*E* = −0.28 eV, *E*_*a*_ = 2.84 eV) than the other one forming *i*-C_5_ (∆*E*= −1.18 eV, *E*_*a*_ = 1.69 eV). Similarly, route 3) also has two C-C cleavage options (marked with grey and red scissors) via β-scission, and the one leading to ethane formation is less dominant than the other one forming *i*-C_6_. The reaction energy of β-scission to form propane is −0.82 eV in route 2), while it is an endothermic process to produce methane with a reaction energy of 1.20 eV.

Furthermore, reaction energy and transition state calculations (details in Supplementary Figs. 27, 28) demonstrated that route 1) isomerization (blue route) is kinetically and thermodynamically the most favorable pathway (primary pathway) among the three routes assessed. The prevalence of *i*-C_4_ and *i*-C_5_ as preferred gaseous products, stemming from route 1) indicated by DFT calculation, aligns well with the distribution of the gaseous species obtained from hydrocracking PP (Supplementary Fig. 3). In all pathways, the carbonium ion either succeeds and circularizes the hydrocracking loop to produce shorter-chain hydrocarbons or is terminated by a hydrogenation reaction.

The catalytic cycle of PO (PE or PP) hydrocracking on Ru_1_-ZrO_2_ is depicted in Fig. 3b. The PO is dehydrogenated on the atomic Ru site of the Ru-O-Zr moiety and then transferred to the Ru-neighboring O site, which is topped with a dangling H and functionalized as the C-C bond breaking site upon the doping of atomic Ru into ZrO_2_. On the O site, a carbonium ion forms, followed by isomerization or rearrangement and subsequent β-scission of the internal C-C bond, which is the rate-determining step. This process forms two shorter-chain hydrocarbons, one of which contains a new carbonium ion. The in situ-generated carbonium ion either undergoes a hydrogenation reaction to desorb or repeats the cycle of isomerization and β-scission. H_2_ dissociation competes with PO dehydrogenation over Ru and supplies surface H species for hydrogenation reaction or spillover to the Ru-neighboring O site for hydrocracking. This mechanism explains why PO dehydrogenation-induced aromatics formation is suppressed as H_2_ pressure increases. The atomic metal (Ru) and acid site (O) in close proximity substantially reduce mass transfer between them, resulting in high efficiency and inhibition of the side reaction of hydrogenolysis to methane.

### Practice

**Fig. 4:**
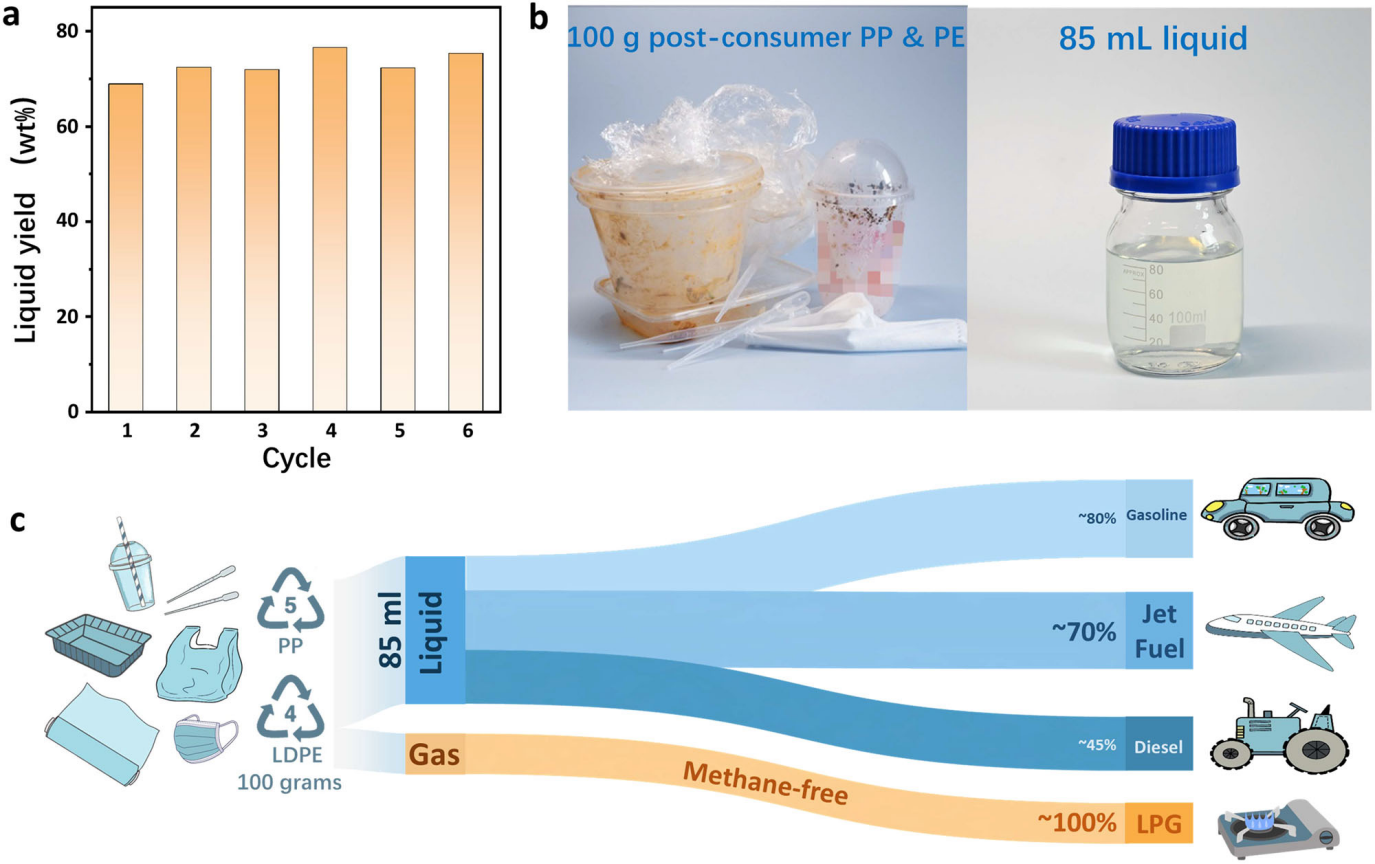
Potential of Ru_1_-ZrO_2_ in practice. **a** Liquid yields in the cycling experiments on Ru_1_-ZrO_2_. **b**, **c** Hydrocracking of 100 g post-consumer PP and PE substrates on Ru_1_-ZrO_2_ at 300 ^o^C under 3 MPa H_2_ for 8 hours: photos of the post-consumer plastics and the liquid product (**b**) and product selectivity (**c**).

The robustness of Ru_1_-ZrO_2_ was assessed by the cycling experiment. No decrease in liquid yield, which is maintained at around 70 wt%, was observed after 6 cycles (Fig. 4a). HRTEM and EDS mapping (Supplementary Fig. 29) revealed Ru species do not undergo aggregation after the reaction. Furthermore, EXAFS and XANES (Supplementary Fig. 30) confirmed the catalyst’s exceptional stability, with Ru remaining embedded as single atoms within the ZrO_2_ lattice after the reaction. In real practice, the one-pot upcycling of 100 grams of mixed post-consumer PP and PE wastes, such as lunch boxes, soft drink cups, masks, bags, pipets, packing films, over Ru_1_-ZrO_2_ at 300 °C under 3 MPa yields 85 mL of centrifuge-collected liquid (Fig. 4b). The hydrocarbon distribution (Supplementary Fig. 31) is identical to that derived from virgin polyolefins (Fig. 1b), consisting of 70% jet-, 80% gasoline- and 45% diesel-ranged fuels in the liquid (Fig. 4c), and 100% LPG in gas product (containing more than 70% C_3_-C_4_ hydrocarbons) without forming methane and ethane.

Driven by global initiatives to reduce carbon emissions and stringent environmental regulations, the demand for sustainable aviation fuel is on a rapid rise over the coming years, starting from 2023, with a compound annual growth rate (CAGR) of 45%^47^. The use of centrifugation to obtain 85 mL of liquid containing 70% jet-fuel-ranged hydrocarbons significantly lowers the cost of post-reaction product separation. It demonstrates the potential for scaling up the production of waste plastic-derived sustainable jet fuel. Furthermore, the absence of methane and ethane in the gas products (LPG) allows them to be directly liquified at ambient pressure, making them suitable for use in heating appliances, cooking equipment, and vehicles.

## Discussion

The hydrocracking PO waste to liquid fuels not only mitigates plastic pollution but also conserves valuable fossil resources, contributing to the advancement of a circular economy. The use of atomic Ru-doped ZrO_2_ marks a paradigm shift in catalyst design, moving from traditional supported-metal manipulation to oxide-based mediation. In this case, the atomic Ru activates its neighboring O atom within the Ru-O-Zr moiety, making it the critical center for selective internal C–C bond cleavage, rather than relying on the Ru site itself. The atomic-level communication between the hydrogenation/dehydrogenation site (metal dopant) and the isomerization/cracking site (neighboring O site) ensures the efficient transfer of PO in between, preventing undesirable hydrogenolysis to methane and ethane at the metal site. Despite ruthenium’s well-established role as a methanation catalyst, this oxide modulation strategy demonstrates its potential to prevent methane formation for the ideal hydrocracking of polyolefins to methane-free fuels. Furthermore, the Ru_1_-ZrO_2_ catalyst has demonstrated its effectiveness in reactions on the hundred-gram scale, offering promising application potential for large-scale chemical upcycling of polyolefin plastics.

## Methods

### Synthesis of Ru_1_-ZrO_2_ and Ru_1_-ZrO_2__500

Ru_1_-ZrO_2_ was prepared by the hard-template method. Typically, Zr(NO_3_)_4_•5H_2_O and Ru(NO)(NO_3_)_x_(OH)_y_ were added to deionized water to form a solution. This solution was added to Carbon black powder which was used as the hard-template. The obtained mixture was dried at 80 °C for 12 hours, and then calcined in air at 500 °C for 6 hours to remove the Carbon black template. Before the hydrocracking reaction, the sample was pretreated in 20% H_2_/Ar with a flow rate of 50 mL/min at 275 °C (300 °C was used for pretreatment when the hydrocracking reaction temperature was 300 °C) to obtain Ru_1_-ZrO_2_. The loading of Ru in Ru_1_-ZrO_2_ used in this work was determined by ICP (Supplementary Table 3) at 1.3 wt%. The Ru_1_-ZrO_2_ catalyst was treated in 20% H_2_/Ar with a flow rate of 50 ml/min at 500 °C for 0.5 hours to obtain Ru_1_-ZrO_2__500. The bare ZrO_2_ as the reference was prepared using the same method without loading Ru.

### Hydrocracking reaction

In a typical hydrocracking reaction, PP, or PE, normal alkane (C_8_ and C_12_), or squalane (4 g), and the Ru_1_-ZrO_2_ catalyst (0.4 g) were loaded into a stainless steel autoclave batch reactor (25 mL). The reactor was then flushed with Ar under 0.4 MPa for 8 cycles, followed by 5 cycles of H_2_ flush with 0.5 MPa pressure above the target reaction pressure. Afterward, the reactor was pressurized with H_2_ to the target pressure (1–4 MPa) at room temperature. Then, the reactor was placed in its associated heater with a temperature controller and heated to target temperatures (250 °C or 300 °C) at a rate of 7.5 °C/min and maintained for target durations (2, 8 hours). Stirring with a rate of 600 rpm was engaged after the temperature reached 160 ± 5 °C. The temperature and pressure were automatically recorded once per minute during the entire reaction. At the end of the reaction, the reactor was cooled to and maintained at 10 °C for 15 minutes to equilibrate the gas and liquid product transformation before the product collection.

The hydrocracking of 100 g of post-consumer PP and PE plastic wastes with 10 g of catalysts was performed in a batch stainless steel autoclave with a volume of 1 L. The post-consumer products, such as lunch boxes, soft drink cups, masks, shopping bags, pipets, and packing films, were picked up from the trash bin and washed before the hydrocracking reaction. The procedure was the same as the mentioned above.

### Product collection and analysis

#### Product collection and calculation

The products were collected and analyzed according to the method depicted in Supplementary Fig. 32. Gas product was charged to a gas sampling bag via the valve-connected tube on the cap of the reactor. The liquid products and insoluble solids, including the catalyst were then collected in a weighted centrifuge tube (${M}_{T}$), and then weighed and denoted as ${M}_{P}$. The liquid product was collected by centrifuging and stored in a vial for GC analysis later. The separated insoluble solids were kept in the centrifuge tube and then washed with *n*-hexane after drying at 80 °C for 24 hours (the products solved in the wash solution of *n*-hexane were mainly under C_22_ which was identical to that of centrifuging collected liquid. Only trace amounts of hydrocarbons C_22_-C_25_ was observed). The centrifuge tube with post-washed solids was dried at 80 °C in an oven for 24 hours, aiming to vaporize all the liquid, and then weighed as ${M}_{1}$.

The masses of products in different phases were calculated using the following formulas:

1 $${M}_{L}=\, {M}_{P}-{M}_{1}$$

2 $${M}_{S}=\, {M}_{1}-{M}_{T}-{M}_{C}$$

3 $${M}_{G}=\, {M}_{{Plastic}}-{M}_{L}-{M}_{S}$$

where ${M}_{L}$, ${M}_{S}$, ${M}_{G}$ and$\,{M}_{{Plastic}}$ are the mass of liquid product, solid residue, gas product, and plastic substrate, respectively. The ${M}_{C}$ is the mass of the catalyst, in most cases in this study it is 0.4 g.

The yield of products (${Y}_{i}$) and conversion were calculated as follows:

4 $${Y}_{i}=\frac{{M}_{i}}{{M}_{{Plastic}}}\times 100\%$$

5 $${Conversion}=\left(1-\frac{{M}_{S}}{{M}_{{Plastic}}}\right)\times 100\%$$

where $i$ is $L$, $S$, $G$, representing liquid product, solid residue, and gas product, respectively. Given the mass of the H_2_ input and consumption was much less than that of plastic substrates, it was ignored when calculating the conversion of plastics.

#### Gas product analysis

The gas product collected in the gas bag was analyzed using a GC (Agilent 8890 Series) with a FID detector and a HP-PlOT-Q column. The method was established and calibrated by using a standard gas reference which involved methane of 0.1 mol%, ethane of 0.1 mol%, propane of 0.1 mol%, isobutane of 0.1 mol%, butane of 0.1 mol%, neopentane of 0.1 mol%, isopentane of 0.1 mol% and pentane of 0.1 mol%. A gastight syringe was used to inject 0.3 mL gas sample to quantify the relative ratio of C_1_-C_6_ hydrocarbons. A typical chromatogram showing the separation of species with retention time is shown in Supplementary Fig. 2.

The mass yield of each component of the gas product can be calculated according to Eq. 6:

6 $${Y}_{{G}_{i}}=\frac{{A}_{i}}{{\sum }_{1}^{6}{A}_{i}}\times {Y}_{G}$$

where ${Y}_{{G}_{i}}$ is the yield of the gas hydrocarbon with the carbon number $i$; ${A}_{i}$ is the chromatographic area of gas hydrocarbon with the carbon number as $i$; $i$ represents carbon number of 1-6; ${Y}_{G}$ is the yield of gas product obtained in equation (4).

#### Liquid products analysis

They were quantitatively analyzed on an Agilent 8890 gas chromatograph equipped with an Agilent HP-5 capillary column and FID. The inlet and detector temperatures were 300 °C, respectively. Typically, 1 μL of liquid product was injected into the chromatogram using a syringe. The standard *n*-alkanes C_7_-C_40_ purchased from Sigma-Aldrich was used to establish the GC method and perform a quantitative analysis. A typical chromatogram for the liquid product and the standard mixture of *n*-alkanes (C_7_-C_40_) obtained from Sigma-Aldrich is shown in Supplementary Fig. 33. The same method was concurrently used for qualitative analysis using GC-MS. (A typical GC-MS result is shown in Supplementary Fig. 34).

The products for hydrocarbons based on carbon number can be calculated according to the following formula:

7 $${Y}_{{L}_{i}}=\frac{{A}_{i}}{{\sum }_{5}^{25}{A}_{i}}\times {Y}_{L}$$

where ${Y}_{{L}_{i}}$ is the yield of the liquid hydrocarbon with the carbon number $i$; ${A}_{i}$ is the chromatographic area of liquid hydrocarbon with the carbon number as $i$; $i$ represents carbon number of 5-25; ${Y}_{L}$ is the yield of liquid product obtained in equation (4).

### Mass balance examination

To calculate the mass balance, the mass yield of gas was measured independently by using the volume of the gas product combined with GC analysis as follows:

The pressure as a function of the gas volume in the reactor at 10 °C was established using a measuring bubbler. Therefore, the post-reaction gas volume can be calculated by using the pressure at the end of the reaction. Combined with the ratio of gaseous species mentioned above, the mass of each species can be calculated independently by using the formula below:

8 $${M}_{{G}_{i}}=\frac{\frac{{A}_{i}}{{k}_{i}}\times {W}_{i}\times {V}_{G}}{22.4}$$

Where ${A}_{i}$ is the chromatographic area of different carbon numbers; ${k}_{i}$ represents the correction factor for the respective component, which is established using 0.3 mL injection of standard gas reference mentioned above and shown in Supplementary Fig. 2; ${W}_{i}$ is the corresponding relative molecular weight; ${V}_{G}$ is the volume of the gas at the end of the reaction.

The mass balance was examined by:

9 $${mass\; balance}=\frac{{\sum }_{1}^{6}{M}_{{G}_{i}}+{M}_{L}+{M}_{S}}{\begin{array}{c}{M}_{{Plastic}}\\ \,\end{array}}\times 100\%$$

Where ${M}_{{G}_{i}}$, ${M}_{L}$ and ${M}_{S}$ are the mass of gas, liquid and solid residue. They are all measured independently based on the above methods. Given that the mass of the H_2_ input and consumption is much less than that of plastic substrates, it is ignored when calculating the mass balance.

Due to the mass loss of products during the independent collection of gas and liquid products and the error of measurements, particularly for the gas volume, the mass balance cannot meet 100% but all still keep the mismatch within 10%. The result of mass balance is shown in Supplementary Fig. 35.

### In situ Pyridine-FTIR characterization

**In situ Pyridine-FTIR** was conducted in a vacuum cell with a Fourier transform infrared spectrometer ThermoFisher iS50r equipped with an MCT detector. Ru_1_-ZrO_2_ sample was pressed to a wafer and loaded into the sample holder. Before pyridine adsorption, the Ru_1_-ZrO_2_ wafer was degassed under vacuum at 150 °C for 20 min and then cooled to room temperature. The background spectra of the samples were acquired in the vacuum before introducing pyridine vapor. The pyridine vapor was introduced into the cell by using a vacuum pump and kept until the intensity of the IR peaks did not increase. Subsequently, the cell was evacuated to remove gas-phase pyridine until the IR peak did not decrease at room temperature and then the wafer was heated to 450 °C to desorb pyridine. After the desorption, the Ru_1_-ZrO_2_ wafer was reduced at 500 °C for 30 min in 20% H_2_/Ar, aiming to in situ obtain Ru_1_-ZrO_2__500. After this reduction, the wafer was cooled to room temperature, and the above procedure was repeated for pyridine adsorption/desorption. The ZrO_2_ was pressed to the wafer for Py-FTIR characterization as a reference. Due to the difference in transmittance and the weight of ZrO_2_ and Ru_1_-ZrO_2_ wafers in ex-situ measurements, the intensity of the peak at 1444 cm^−1^ was normalized to the spectra of ZrO_2_ and Ru_1_-ZrO_2_ in Fig. 2i.

### H_2_/D_2_ exchange experiment

H_2_/D_2_ exchange experiment was performed at 250 °C and mass spectrometry was used to record the signal intensities. Typically, 50 mg catalyst was loaded into the reactor and 10% H_2_/Ar was introduced with a flow rate of 20 ml/min at 250 °C. After the stabilization of the mass spectrometry signal, the flow of 10% H_2_/Ar was switched to the flow of D_2_/Ar with a rate of 20 mL/min. The signal of M/Z = 3 (HD) was recorded throughout the process. After the testing of Ru_1_-ZrO_2_ was completed, the reactor was gradually heated to 500 °C at a rate of 10 °C/min under the atmosphere of 20 ml/min 20% H_2_/Ar for in situ reduction for 0.5 hours, aiming to obtain the sample of Ru_1_-ZrO_2__500 in situ. Subsequently, the reactor was cooled down to 250 °C to conduct the H_2_-D_2_ exchange experiment again for Ru_1_-ZrO_2__500 catalyst.

### Computational details

All DFT calculations were performed using the Vienna Ab Initio Simulation package (VASP 5.4.4)^48,49^. The generalized gradient approximation (GGA) with PBE exchange and correlation functional was used to account for the exchange-correlation energy^49,50^. The kinetic energy cutoff of the plane wave basis set was set to 400 eV. The threshold for energy convergence for each iteration was set to 10^−5 ^eV. Geometries were assumed to be converged when forces on each atom were less than 0.05 eV/Å. Gaussian smearing of the population of partial occupancies with a width of 0.05 eV was used during iterative diagonalization of the Kohn-Sham Hamiltonian.

The bulky ZrO_2_ unit cell in the tetragonal phase was first fully optimized. The optimized lattice vectors of a = b = 3.612 Å and c = 5.212 Å have a good agreement with the experiment parameters^51^. The most stable (101) surface of ZrO_2_ tetragonal phase was simulated by a 3×4×1 supercell slab model including three ZrO_2_ sub-layers (each includes two oxygen atomic layers and one Zr atomic layer) separated by a vacuum layer with a thickness of 15 Å along the surface normal direction to avoid spurious interactions between periodic slab models. The bottom two layers of ZrO_2_ were fixed, while the rest was allowed to relax during the geometry optimization. One Zr atom on the surface was replaced by Ru to create a Ru-O-Zr moiety. The lattice parameters were fixed throughout the surface calculations. Corrections for on-site Coulomb interactions by use of the DFT + U procedure were done with effective U = 4 eV for Zr. The Brillouin zone integration and k‐point sampling were restricted to the gamma point. The nudged-elastic band method with the improved tangent estimate (CI-NEB) was used to determine the minimum energy path and to locate the transition state structure for each elementary reaction step^52^. The transition state was confirmed by observing only one imaginary frequency corresponding to each reaction coordinate.

The alkane of C11 with the branched methyl groups was used as a model compound. The adsorption energy of the reaction intermediate was calculated as ${E}_{{ads}}\,=\,{E}_{{adsorbate}+{surface}}-\,{E}_{{adsorbate}}-\,{E}_{{clean}-{surface}}$. The activation energy (${E}_{a}$) of a chemical reaction was defined as the energy difference between the initial and transition states, while the reaction energy (Δ*E*) was defined as the energy difference between the initial and final states.

## Supplementary information

Supplementary Information

Transparent Peer Review file

## Source data

Source Data

## Data Availability

All data generated or analyzed during this study are included in this published article (and its supplementary information file). Source data are provided with this paper.
