# Supplementary material for: Upcycling polyolefins to methane-free liquid fuel by a Ru1-ZrO2 catalyst
Source: Nat Commun. 2025 Mar 21;16:2800. doi: 10.1038/s41467-025-57998-x (PMC11928669; doi:10.1038/s41467-025-57998-x)
Supplement: Supplementary file 1 — Supplementary Information [file 41467_2025_57998_MOESM1_ESM.pdf]

Supplementary information

for

Upcycling polyolefins to methane-free liquid fuel by a Ru_1-ZrO_2 catalyst

Jicong Yan†, Guanna Li†, Zhanwu Lei†, Xiaolu Yuan, Junting Li, Xiaoru Wang, Bo Wang, Fuping Tian, Tao Hu, Lei Huang, Yujia Ding, Xiaoke Xi, Feng Zhu, Shuo Zhang, Jiong Li, Yu Chen, Ruiguo Cao, Xiang Wang*

Corresponding author: xiangwang@dlut.edu.cn (X. W.)

The file includes:

Supplementary Methods

Supplementary Tables 1 to 5

Supplementary Figures 1 to 36

Supplementary References 1 to 5

Supplementary Methods

Materials

$Ru(NO)(NO_3)_x(OH)_y$, x+y=3, (Ru 1.5% w/v), and $Zr(NO_3)_4 \cdot 5H_2O$ were purchased from Rhawn Reagent Co. BLACK PEARLS 2000 (BP2000) was purchased from Cabot Co. $Cu(NO_3)_2 \cdot 3H_2O$ and Squalane (95%) was purchased from Aladdin Biochemical Technology Co. Ltd. *n*-Pentane (99.5%) was obtained from Energy Chemical Co. ZrO_2 was purchased from Sinopharm Chemical Reagent Co. SiO_2 was obtained from Beijing Xudong Chemical Co. Ltd. *n*-Hexane (AR), *n*-Octane (AR) and *n*-Dodecane (AR) were available from Tianjin Kemiou Chemical Reagent Co. Ltd. Dichloromethane-d2 (99.8 atom % D) was purchased from Cambridge Isotope Laboratories. Polypropylene (PP, average Mw~260,000 Da, melt index 30 g/10 min, **Supplementary Table 4** and **Supplementary Fig. 36a**) and polyethylene (PE, average Mw~120,000 Da, melt index 50 g/10 min, **Supplementary Table 4** and **Supplementary Fig. 36b**) substrates were obtained from Sinopec Maoming Petrochemical Co. Postconsumer PP and PE products, e.g. lunch boxes, masks, non-woven fabric, Zip-loc bags, pipets were recycled from trash bins and then washed and cut into fragments for hydrocracking reaction. Finally, a 25-mL autoclave reactor and a 1000-mL autoclave reactor were purchased from Anhui Kemi Instrument Co. Ltd.

A standard gas reference containing 0.1 mol% of each component: methane, ethane, propane, isobutane, butane, neopentane, isopentane and pentane, was purchased from Dalian Kylingas Co., Ltd. and used for the calibration of GC for gas product analysis.

A standard liquid reference containing *n*-alkanes (C_7-C_{40}) was purchased from Sigma-Aldrich and used to calibrate GC for liquid product analysis.

Catalyst stability test

For the first cycle, following the above procedure in hydrocracking reaction, 0.4 g fresh Ru_1-ZrO_2 catalyst and 4 g PP were loaded into the reactor and the reactor was pressurized to 3 MPa followed by heating to 300 ℃ and held for 8 hours. After the reaction, all the gas and liquid products

were separated and the used catalyst was collected by centrifuging. The post-reacted catalyst was refilled into the reactor and replenished with ~10% of catalysts due to the loss of catalyst during the production collection and separation to carry out the second cycle reaction. After the second cycle, the post-reacted catalyst was cleaned using the following procedure. The post-reacted catalyst was calcined in air at 500 °C for 6 hours to remove organic residue and then pretreated with the same procedure used for the fresh catalyst as mentioned above. This clean-up process was repeated every other cycle.

Product characterization

Gel permeation chromatography (GPC) analyses of the molecular weight and molecular weight distribution (PDI = M_w/M_n) of the polymers at 150 °C were performed on a high-temperature chromatograph, PL-GPC 220 instrument equipped with a triple detection array, including a differential refractive index (RI) detector, a two-angle light scattering (LS) detector, and a four-bridge capillary viscometer

Nuclear magnetic resonance (NMR) analysis was conducted with a BRUKER AVANCE III 600 at room temperature for the liquid product diluted in CD_2Cl_2. Chemical shifts (ppm) were calibrated using the residual proton signals of the solvent and referenced to tetramethylsilane (TMS).

Ultraviolet-visible (UV-Vis) spectra were obtained on Shimadzu UV-1900. The liquid sample obtained after the reaction was meticulously diluted with *n*-hexane to keep its concentration at $1.2*10^{-6}$ before being transferred into quartz cuvettes for testing within the 200-600 nm range while concurrently utilizing *n*-hexane as a reference.

Catalyst characterization

Transmission electron microscope (TEM) and High-Resolution Transmission Electron Microscope (HRTEM) observations were conducted using a JEM-F200 transmission electron microscope with an acceleration voltage of 200 kV. Low-magnification HAADF-STEM images

were obtained using an FEI Talos F200X instrument operated at 200 kV. Atomic resolution HAADF-STEM images were acquired using a double aberration-corrected JEM-ARM300F2 (S) TEM operating at 300 kV, which offers enhanced resolution capabilities. Energy dispersive spectroscopy (EDS) mappings were carried out using a FEI Talos F200X microscope equipped with a Super X-EDS system, also operated at 200 kV.

X-ray diffraction (XRD) patterns were collected by using a Rigaku, SmartLab. X-ray diffractometer equipped with Cu Ka radiation (λ = 1.5418).

X-ray photoelectron spectra (XPS) were performed by using a Kratos Axis supra+ with monochromatic Al Ka X-ray sources (1486.6 eV).

The X-ray Fluorescence (XRF) results were obtained on the BRUKER AXS S8 TICER.

Inductively Coupled Plasma-Optical Emission Spectroscopy (ICP-OES) was used to determine the loading amount of Ru in the sample. Before ICP analysis, 20 mg of the sample was dissolved in 24 mL of aqua regia, then hydrothermally treated at 150 °C for 12 hours. Subsequently, the sample was further diluted with deionized water.

X-ray Absorption Fine Structure (XAFS) data were collected at room temperature at the BL11B beamline of the Shanghai Synchrotron Radiation Facility (SSRF). A monochromatic X-ray beam was generated using a double-crystal Si(311) monochromator. The beamline was calibrated using a Ru foil. The first peak of the first derivative curve of XAFS data from the Ru foil was calibrated to 22117 eV. The data collection was conducted in transmission mode for the reference foil and in fluorescence mode for samples using a Kr-filled Lytle detector. All XAFS data were aligned, merged, and normalized in Athena from the IFEFFIT suite. The built-in AUTOBK algorithm was used to minimize background below R_{bkg}=1.0 Å. EXAFS data of Ru_1-ZrO_2 and Ru_1-ZrO_2_500 were modeled in Artemis using one Ru-O path calculated from RuO_2 structure and one Ru-Ru path calculated from metallic Ru structure, and were fitted in k, k^2, and k^3 weighting over a k-range of 2-9 $Å^{-1}$ and an R-range of 1-3 Å.

Temperature-programmed reduction with H_2 (H_2-TPR) analysis was carried out with a chemisorption analyzer (BSD-Chem C200). The sample amount was about 50 mg. After pretreatment at 200 °C under N_2 for 30 minutes, the sample was heated under 10% H_2/N_2 with a flow rate of 30 mL/min from 50 °C to 800 °C at a heating rate of 10 °C /min.

Ammonia temperature-programmed desorption (NH_3-TPD-MS) was performed with Micromeritics 2920 with a mass spectrometer of Hiden Analytical DECRA. The catalyst was degassed at 150 °C for 30 min in a He flow of 30 mL/min. After the degassing, 30 mL/min of 10% NH_3/He was introduced for sufficient adsorption, followed by a purge with He of 30 ml/min until the baseline stabilized. Afterwards, the reactor was heated from 100 °C to 800 °C with a ramp of 10 °C /min, and the desorption NH_3 was recorded.

Supplementary Tables

Supplementary Table 1. The yields of methane and ethane products at different pressures of H_2 as shown in **Fig. 1b**.

**Product**	**Mass yield %**				
	**1 MPa**	**1.5 MPa**	**2 MPa**	**3 MPa**	**4 MPa**
**Methane**	0.214	0.163	0.040	0.025	0.025
**Ethane**	0.483	0.374	0.158	0.117	0.116

Reaction conditions: 300 ℃, 8 hours, 600 RPM, 4 g PP, 0.4 g Ru_1-ZrO_2.

Supplementary Table 2. EXAFS fitting results of Ru_1-ZrO_2 and Ru_1-ZrO_2_500.

Sample	Path	S_0^2	ΔE_0 (eV)	CN	R (Å)	Debye–Waller ($Å^2$)	R-factor
	Ru-O		-	4.0±0.6	2.0±0.02		
**Ru_1-ZrO_2**		0.85				0.0042	0.015
	Ru-Ru		1.0±1.6	1.7±0.6	2.7±0.02		
	Ru-O			3.1±0.5	2.1±0.03		
**Ru_1-ZrO_2_500**		0.85	3.2±2.6			0.0020	0.049
	Ru-Ru			2.1±0.6	2.7±0.03		

Supplementary Table 3. The loadings of Ru in ZrO_2, Ru_1-ZrO_2 and Ru_1-ZrO_2_500 samples characterized using XPS, EDS and ICP.

Sample	Ru wt%			Surface Zr-O mole ratio (XPS)
	XPS	EDS	ICP	
ZrO_2	0	0	0	0.29
Ru_1-ZrO_2	1.75	1.88	1.28	0.29
Ru_1-ZrO_2_500	1.66	1.92	1.35	0.35

Supplementary Table 4. Molecular weight analysis of polyolefin using GPC.

Sample	Product model	Melt index (g/10 min)	M_w (Da)	M_n (Da)	M_P (Da)
**PP**	Z30S	30	262, 137	30, 223	142,605
**PE**	M1850A	50	120, 409	10, 525	60, 630

Supplementary Table 5. Integrated values of the spin-polarized COHP denote net bonding characters for the O-H interactions of RuOHZr and ZrOHZr moieties of the surface slab models of Ru_1-ZrO_2 and ZrO_2

COHP	Atom	Atom	Distance *d*(O-H)	ICOHP spin_up	ICOHP spin_down
RuOHZr	O	H	0.97442	-3.86930	-3.88393
ZrOHZr	O	H	0.97404	-3.89581	-3.90427

Supplementary Figures

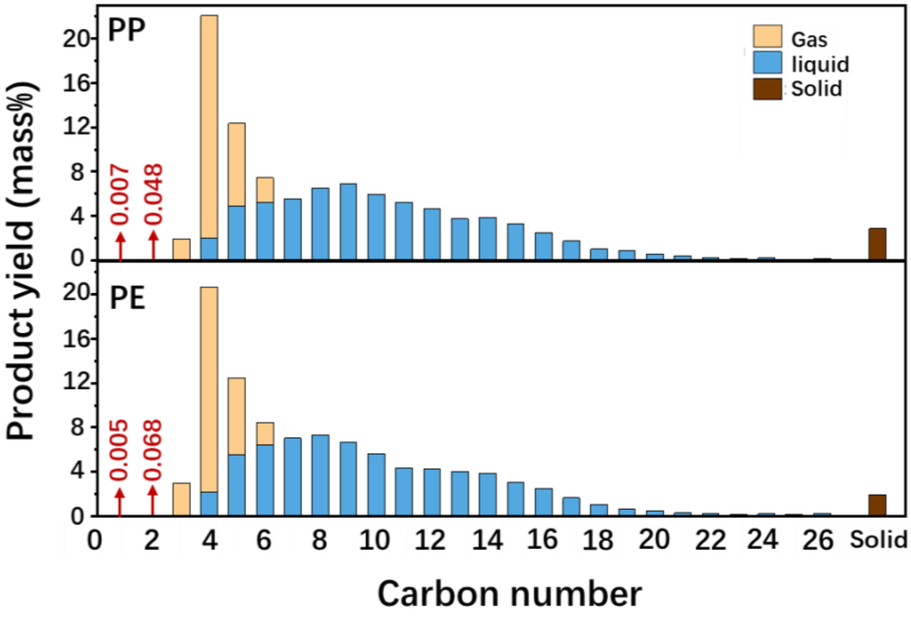

Supplementary Figure 1. Hydrocarbon distribution based on carbon number after hydrocracking of PP and PE over Ru_1-ZrO_2 at 250 ℃ under 3 MPa of H_2 for 8 hours. The gas and liquid products are labeled in orange and blue bars, respectively. The solid residue is shown in brown.

The yields of CH_4 and C_2H_6 are too low to show in **Supplementary Fig. 1**. The trace amount of them was detected by using GC with an FID detector (**Supplementary Fig. 2**) which is far more sensitive than a TCD detector, and the number was marked in **Supplementary Fig. 1**.

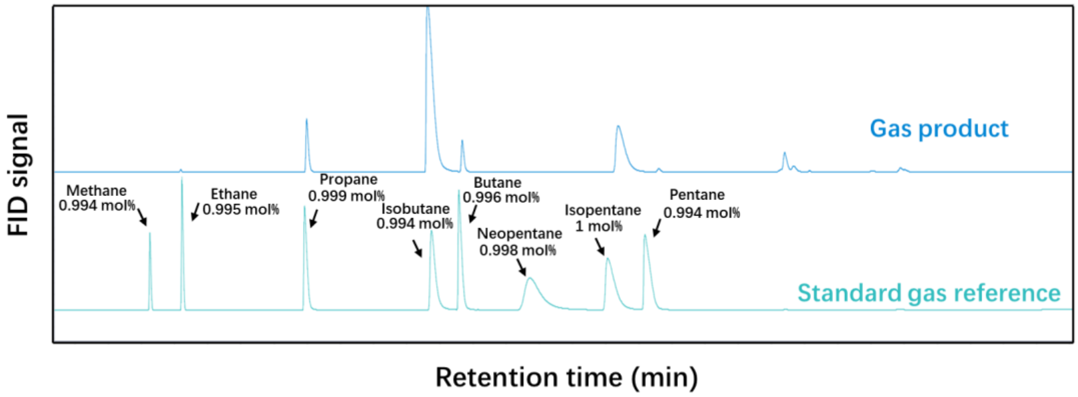

Supplementary Figure 2. GC chromatogram recorded using an FID detector of gas products from the reaction of PP over Ru_1-ZrO_2 at 250 °C under 3 MPa of H_2 for 8 hours, and the standard gas reference with certified concentrations purchased from Dalian Kylingas Co., Ltd.

The GC chromatogram of a standard alkane mixture with a certified concentration at around 1 mol% was used to identify the species of the gas products. At the retention times for the CH_4 and C_2H_6, the peaks are barely seen in the GC chromatogram even if using a sensitive detector FID, indicating their trace amount.

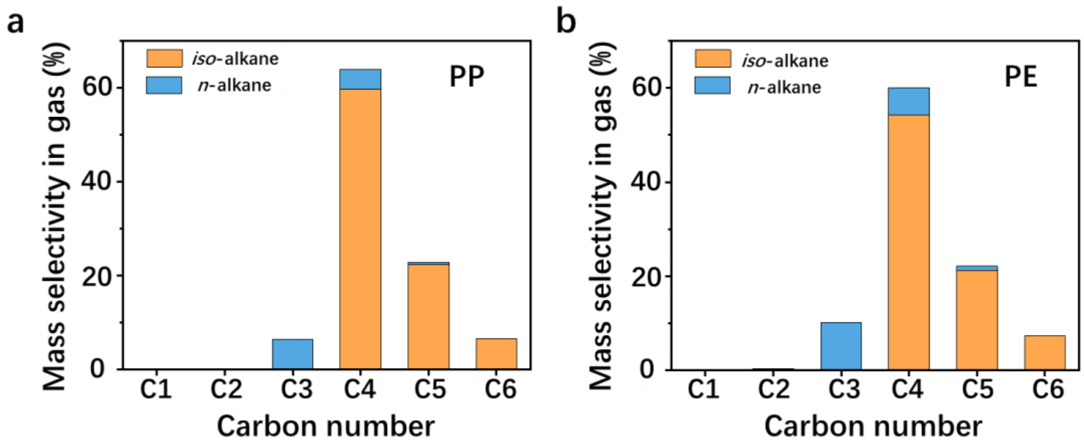

Supplementary Figure 3. Hydrocarbon distribution based on carbon number in the gas products obtained after the hydrocracking of **(a)** PP and **(b)** PE over Ru_1-ZrO_2 at 250 ℃ under 3 MPa of H_2 for 8 hours.

The distributions and yields of gas phase *iso*-alkanes using less branched PE substrates are similar to those using PP substrates. This indicates that isomerization occurs during the reactions regardless of PP or PE substrate, leading to similar gas phase products.

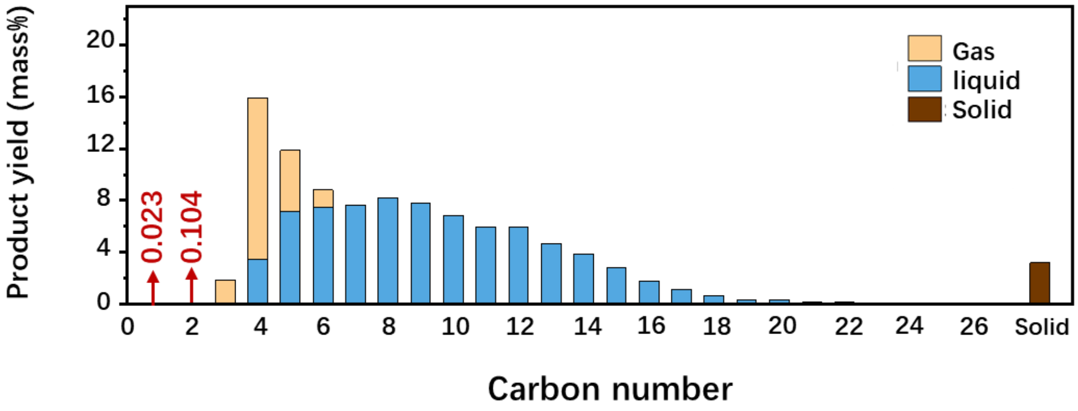

Supplementary Figure 4. Hydrocarbon distribution based on carbon number after hydrocracking of PP over Ru_1-ZrO_2 at 300 °C under 3 MPa of H_2 for 2 hours.

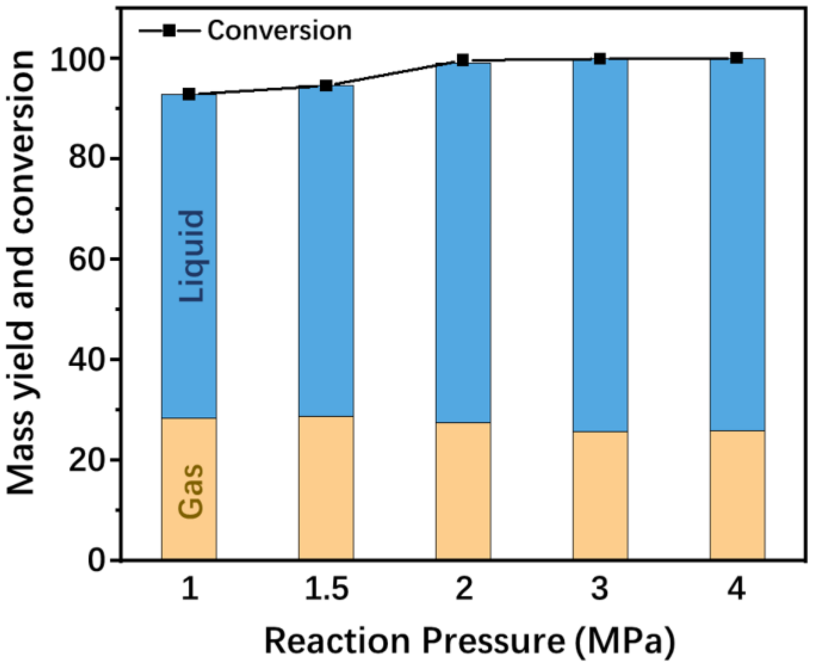

Supplementary Figure 5. The mass yields of gas and liquid products, and the conversion of PP over Ru_1-ZrO_2 after 8 hours at 300 ℃ under 1-4 MPa of H_2.

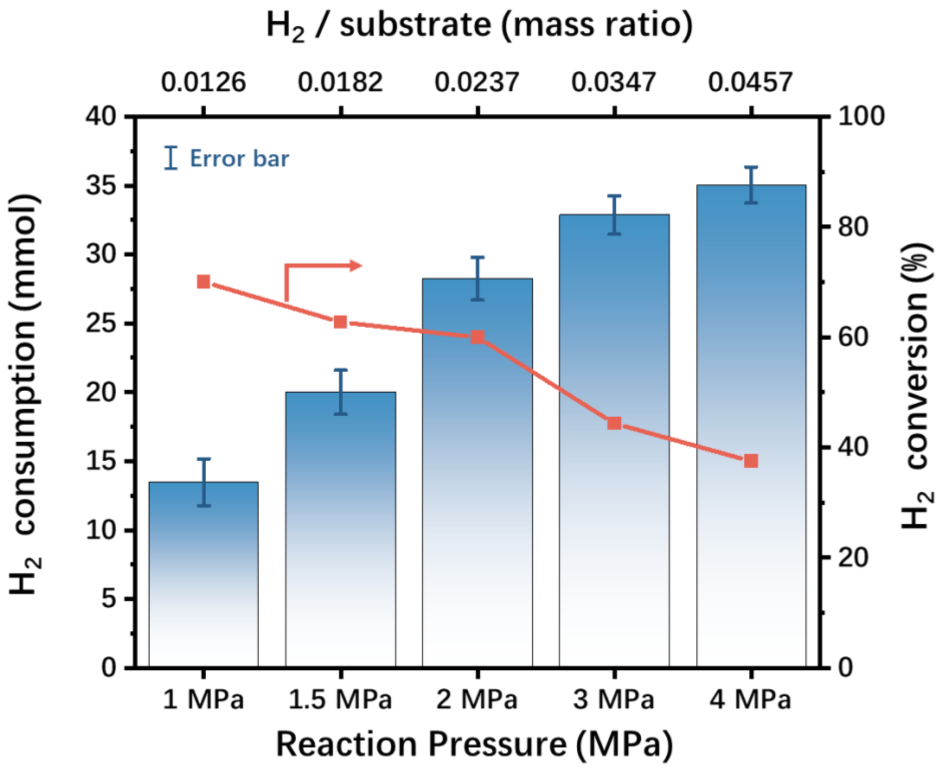

Supplementary Figure 6. Consumption and conversion of H_2 after 8 hours of hydrocracking PP over Ru_1-ZrO_2 at 300 °C under 1-4 MPa of H_2.

The consumption of H_2 was calculated by analyzing the gas products combined with the measured volume of the reactor (See Product Analysis in the Method section). As the amount or the pressure of H_2 increases (while maintaining a constant substrate mass), the H_2 conversion gradually decreases, but the consumption of H_2 increases. Given that the alkanes dominate the product hydrocarbons, one C-C bond cleavage consumes one H_2 molecule. Therefore, the high H_2 consumption indicates more cleavages and the higher pressure of H_2 results in more C-C bonds being cleaved.

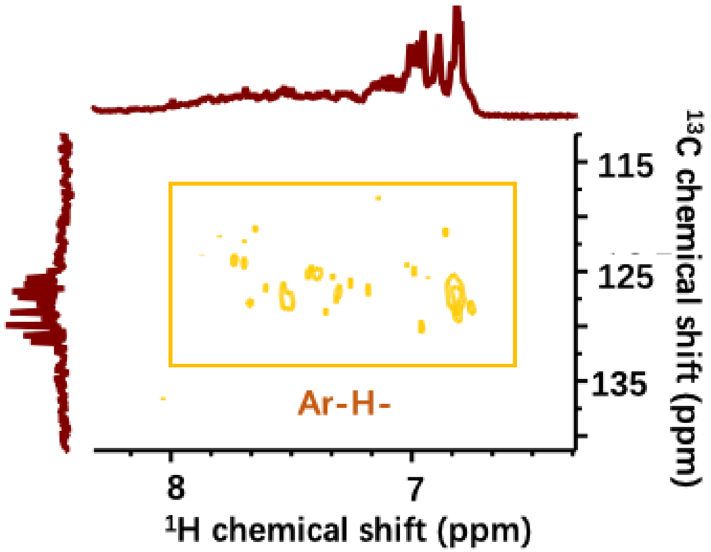

Supplementary Figure 7. $^{13}C-^{1}H$ HSQC NMR of the liquid product obtained from PP hydrocracking over Ru_1-ZrO_2 in 1.5 MPa, 300 °C for 8 hours.

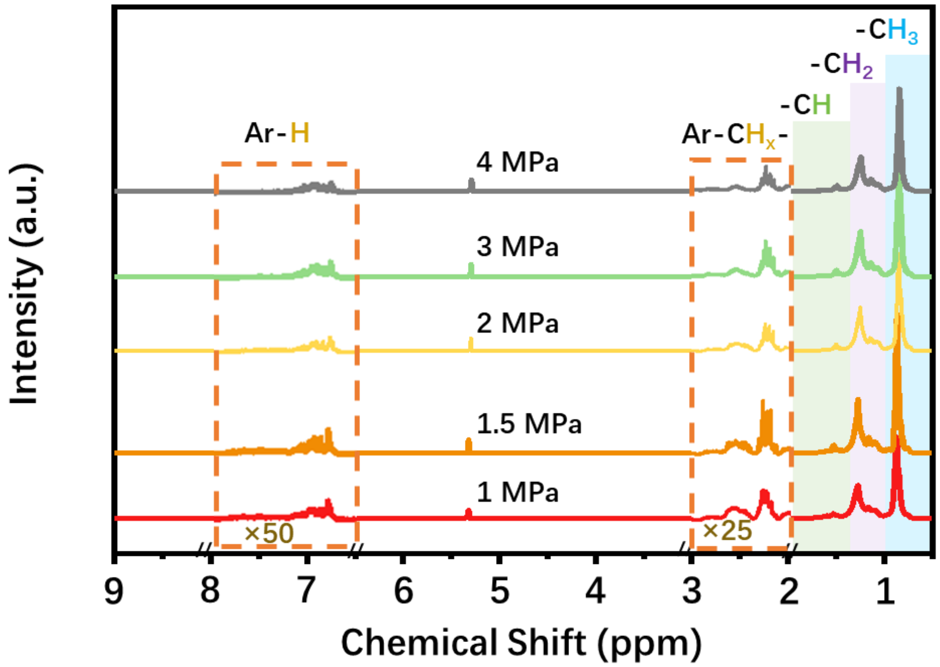

Supplementary Figure 8. ^{1}H NMR of the liquid product of hydrocracking PP on Ru_1-ZrO_2 at 300 ℃ under 1-4 MPa H_2 for 8 hours.

The appearance of ^{1}H nuclear magnetic resonance (NMR) signals in the regions of 6.5-9.0 ppm and 2.0-3.0 ppm of the liquid is due to the presence of aromatics. However, in the 5-6 ppm region, only signals corresponding to the solvent CD_2Cl_2 were observed. Other unsaturated olefins were not detected due to their low concentration. The relative amount of aromatics was estimated by using the ratio of the integration intensity of aromatics and the integration intensity of all species detected by ^{1}H NMR.

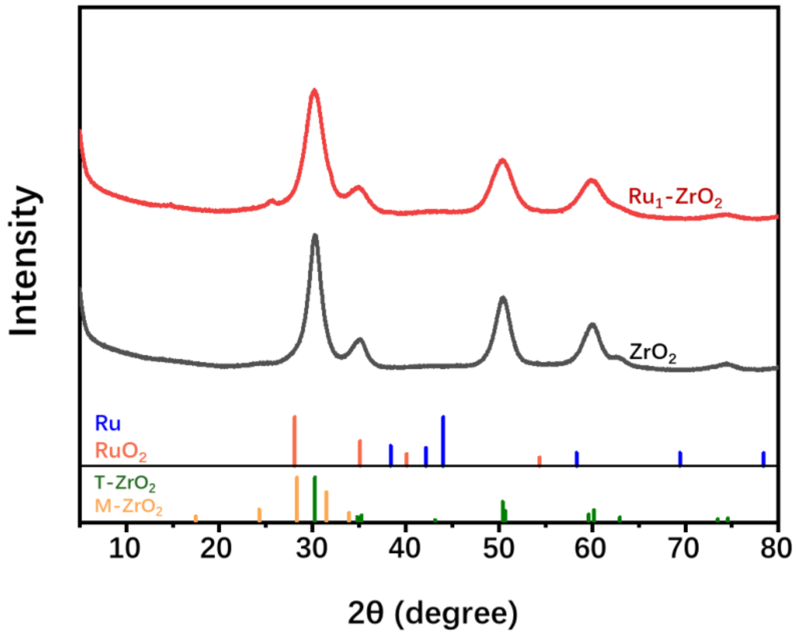

Supplementary Figure 9. XRD patterns of Ru_1-ZrO_2 and ZrO_2

The XRD patterns of Ru_1-ZrO_2 and ZrO_2 catalyst show the peaks of tetragonal ZrO_2 (T-ZrO_2, PDF#50-1089) at 30.27°, 34.81°, 50.38°, 60.20°, corresponding to (0 1 1), (0 0 2), (1 1 2), (1 2 0) crystal faces, respectively. In addition, a small diffraction peak is observed at ~25°, which is likely due to the monoclinic ZrO_2 (M-ZrO_2, PDF#97-006-8727). No characteristic diffraction peaks of hexagonal close-packed Ru (PDF#06-0663) and RuO_2 (PDF#97-001-5071) were observed, generally appearing at 44°. This indicates that Ru is highly dispersed on the ZrO_2 surface.

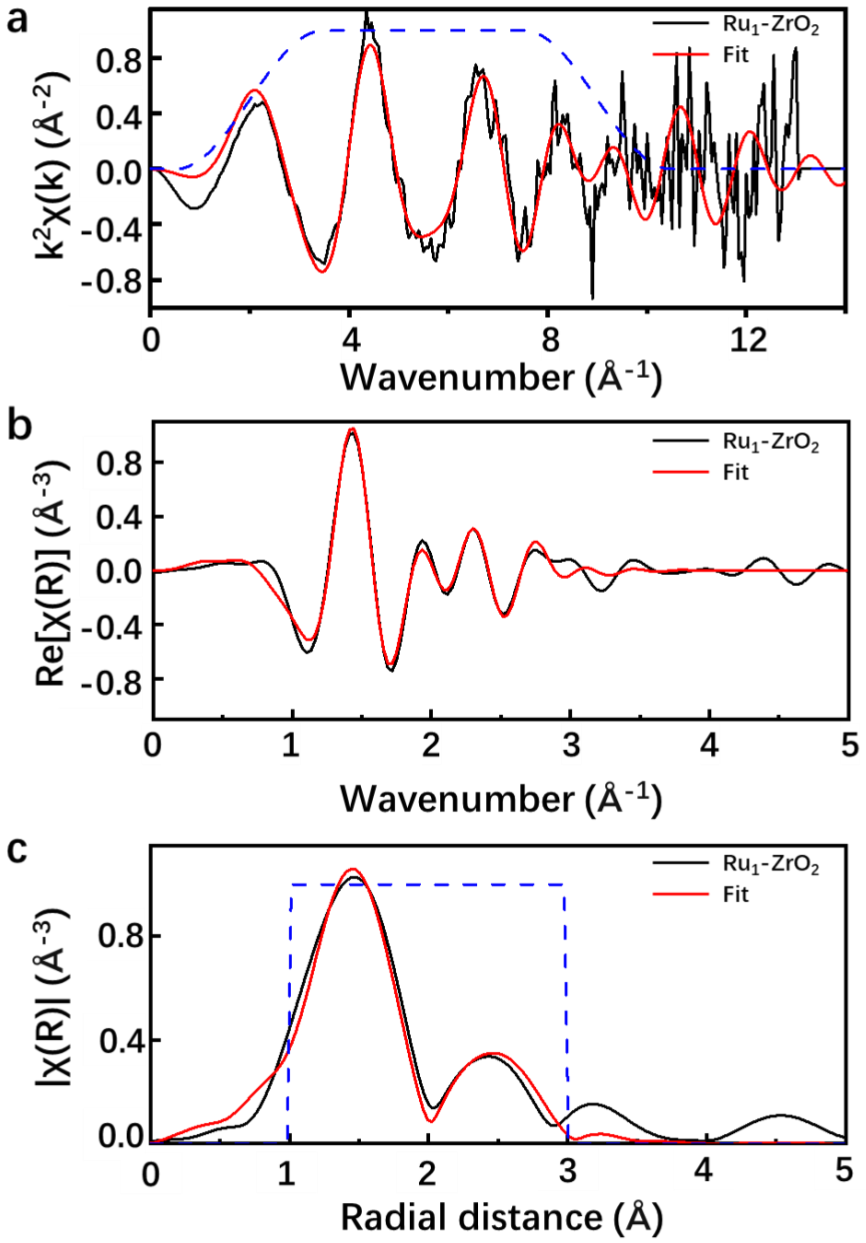

Supplementary Figure 10. Ru K-edge EXAFS data fitting shown in **(a)** $k^2\chi(k)$, **(b)** Re[χ(R)] and **(c)** |χ(R)| plots, using black lines for experimental data, red lines for fit, and blue dashed lines for the window of Fourier transform in k-space, and the window of backward Fourier transform in R-space.

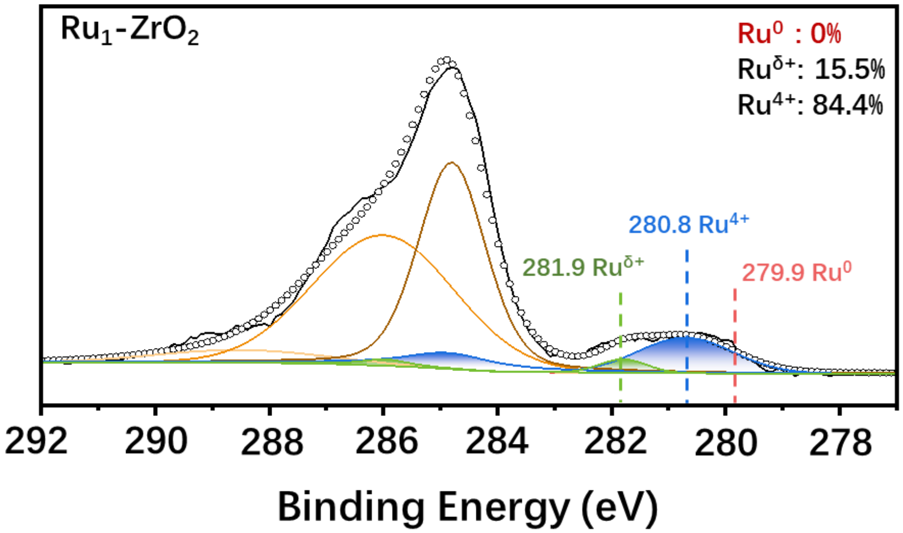

Supplementary Figure 11. Ru 3*d* XPS spectra of Ru_1-ZrO_2

The Ru 3*d* XPS spectrum exhibits two contributions, Ru^{4+} 3$d_{5/2}$ and $Ru^{\delta+}$ 3$d_{5/2}$, located at respectively 280.8 eV and 281.9 eV, while metallic Ru^0 3$d_{5/2}$ (279.9 eV) was not detected. This demonstrates that only the oxidized state of ruthenium (Ru) exists in Ru_1-ZrO_2.[1,2] The spectrum has been fitted by considering two resolved doublets (with a spin–orbit splitting of ~4.17 eV between 3$d_{3/2}$ and 3$d_{5/2}$).

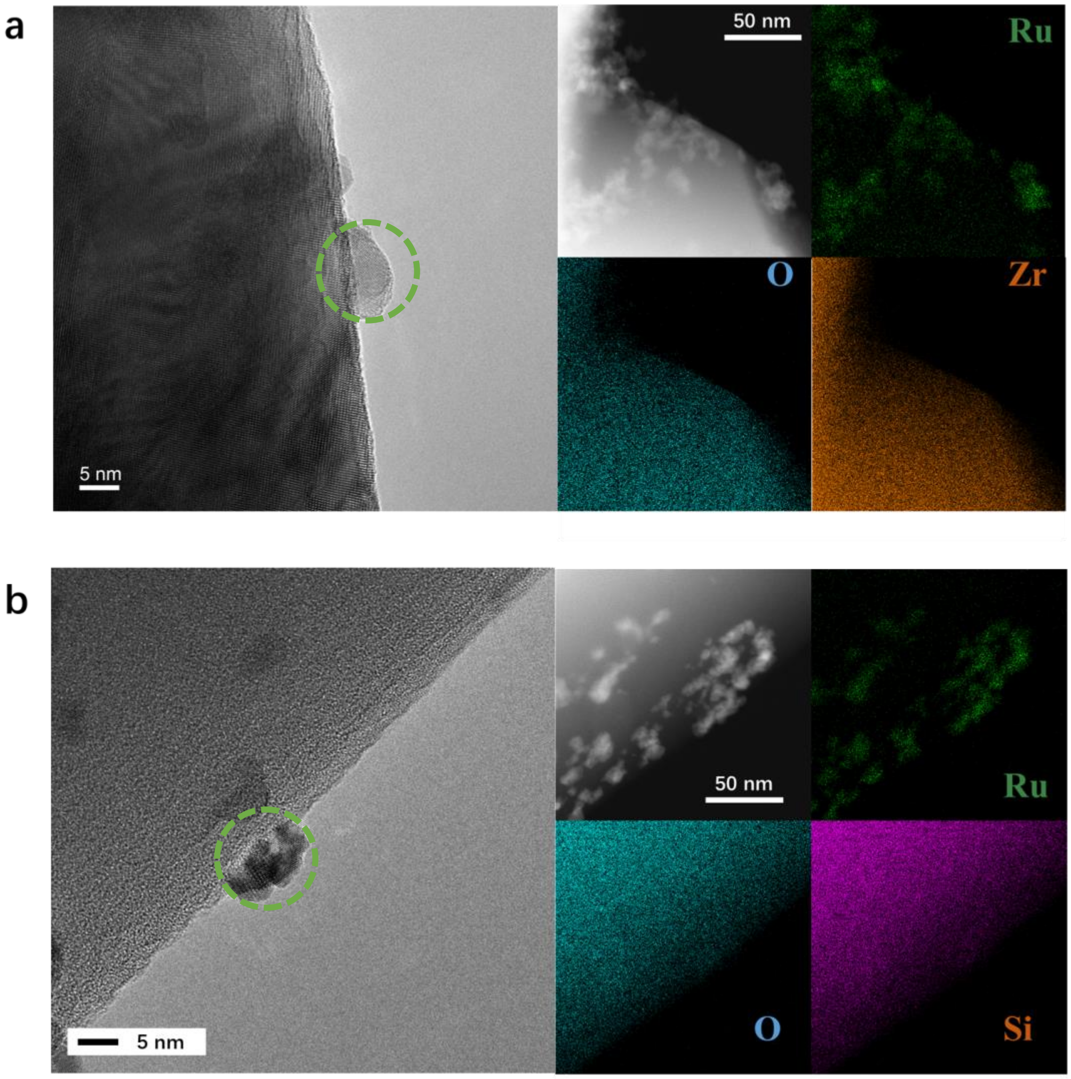

Supplementary Figure 12. HRTEM and EDS element mapping images of **(a)** Ru/ZrO_2 and **(b)** Ru/SiO_2.

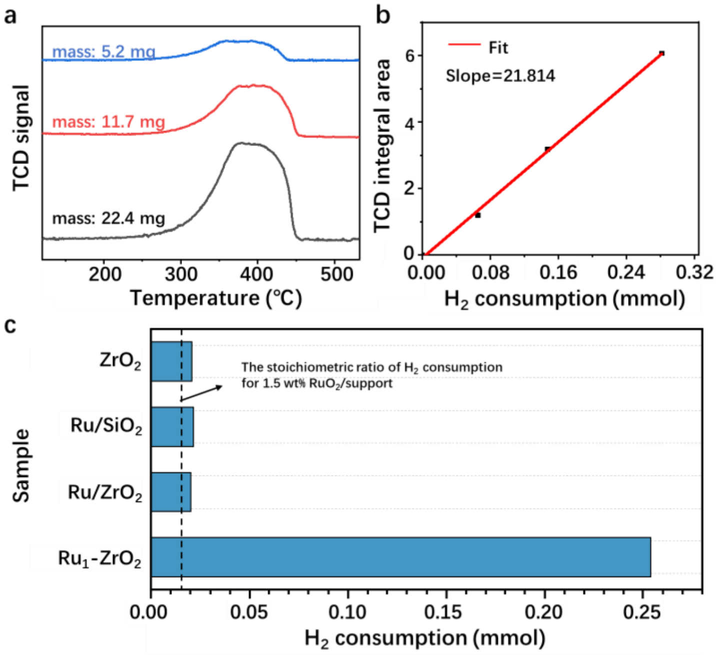

Supplementary Figure 13. (a) H_2-TPR curves of CuO with different masses. **(b)** Integration of the reduction peak in **(a)** as a function of stoichiometric H_2 requirement for reducing CuO. **(c)** H_2 consumptions of ZrO_2, Ru/SiO_2, Ru/ZrO_2 and Ru_1-ZrO_2 calculated by using the calibration established in **(b)** with the integrations of the reduction peaks in **Fig. 2f**.

The amounts of H_2 consumed by supported 1.5 wt% Ru/ZrO_2 and 1.6 wt% Ru/SiO_2 are almost identical to the stoichiometric value for reducing the RuO_2 with the loading of 1.5 wt% (assume Ru in supported Ru/ZrO_2 and Ru/SiO_2 is in the form of RuO_2).

Calibration curve is established as follows:

Weigh 5.2 mg, 11.7 mg, and 22.4 mg of CuO for H_2-TPR testing , obtaining TCD signal integration areas of 1.19, 3.18, and 6.09, respectively. The relationship between the consumption of H_2 and the amount of CuO was depicted as the following reaction equation.

$$CuO + H_2 = Cu + H_2O$$

Linear fitting was performed on the hydrogen consumption and integration area, resulting in a slope of 21.8.

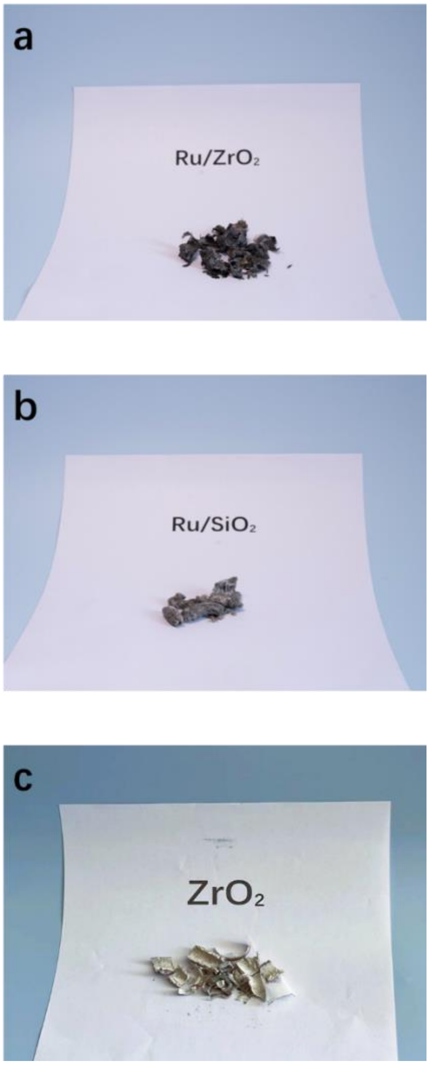

Supplementary Figure 14. Photos of the post-reacted solid residues over **(a)** Ru/ZrO_2, **(b)** Ru/SiO_2 and **(c)** ZrO_2.

The post-reaction solid residue was weighted and showed around 4.4g, which is the sum of the 0.4 g of catalyst and 4 g of PP substrate. This result indicates that the reaction was not initiated on Ru/ZrO_2, Ru/SiO_2 and ZrO_2.

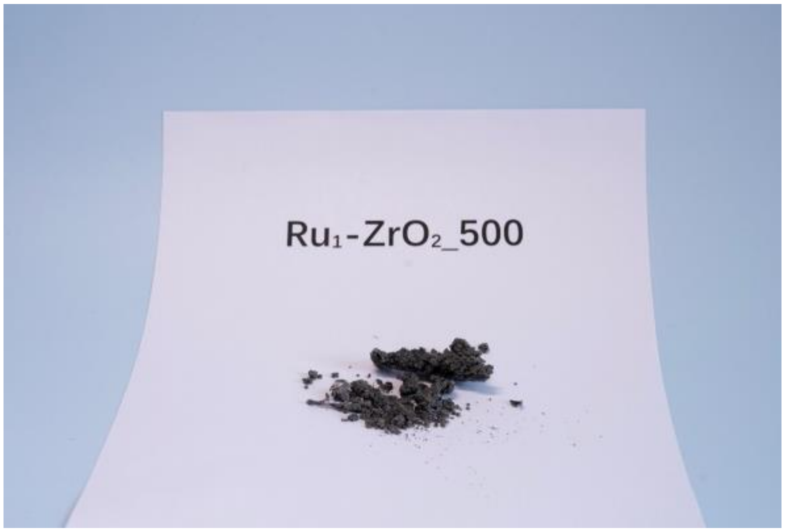

Supplementary Figure 15. Photo of the post-reacted solid residues over Ru_1-ZrO_2_500.

The post-reaction solid residue was weighted and showed around 4.4g, which is the sum of the 0.4 g of catalyst and 4 g of PP substrate. This result indicates that the reaction was not initiated on Ru_1-ZrO_2_500.

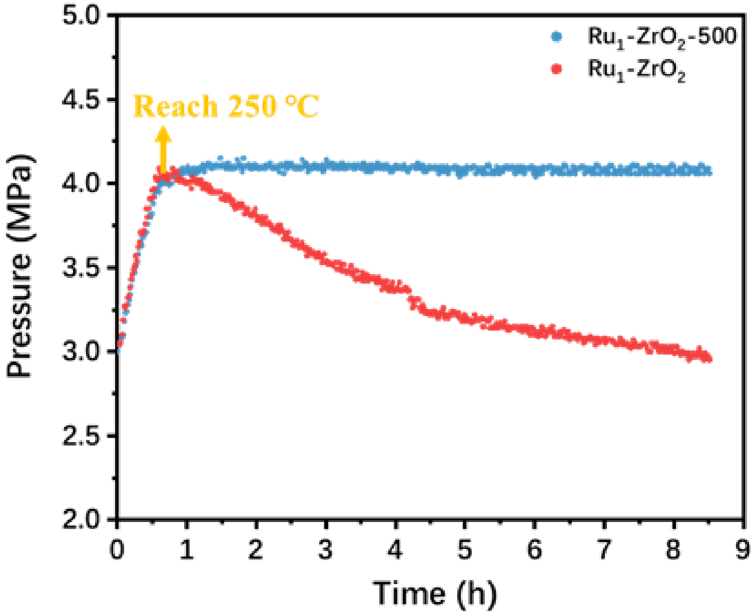

Supplementary Figure 16. Pressure variation during the reaction of Ru$_1$-ZrO$_2$ and Ru$_1$-ZrO$_2$-500 at 250 ℃ under 3 MPa H_2 (room temperature) for 8h.

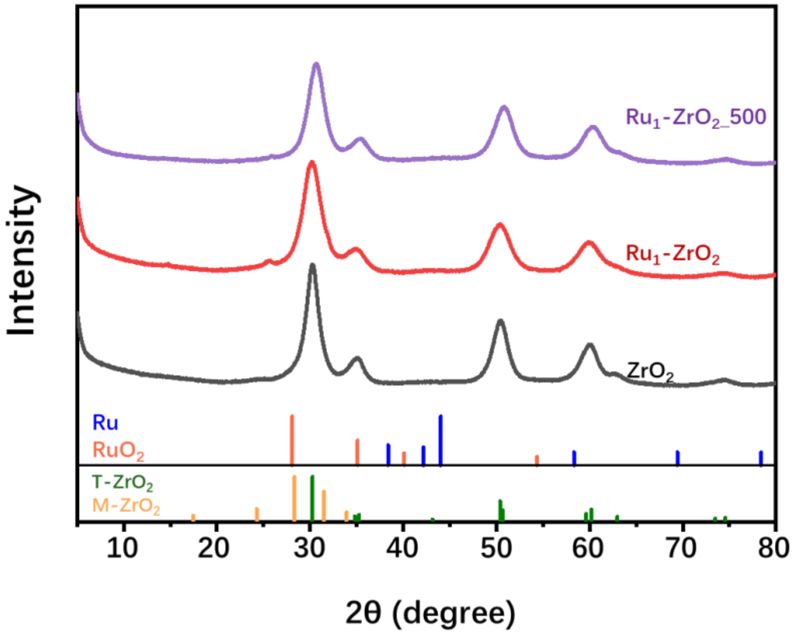

Supplementary Figure 17. XRD of Ru_1-ZrO_2_500, Ru_1-ZrO_2 and ZrO_2.

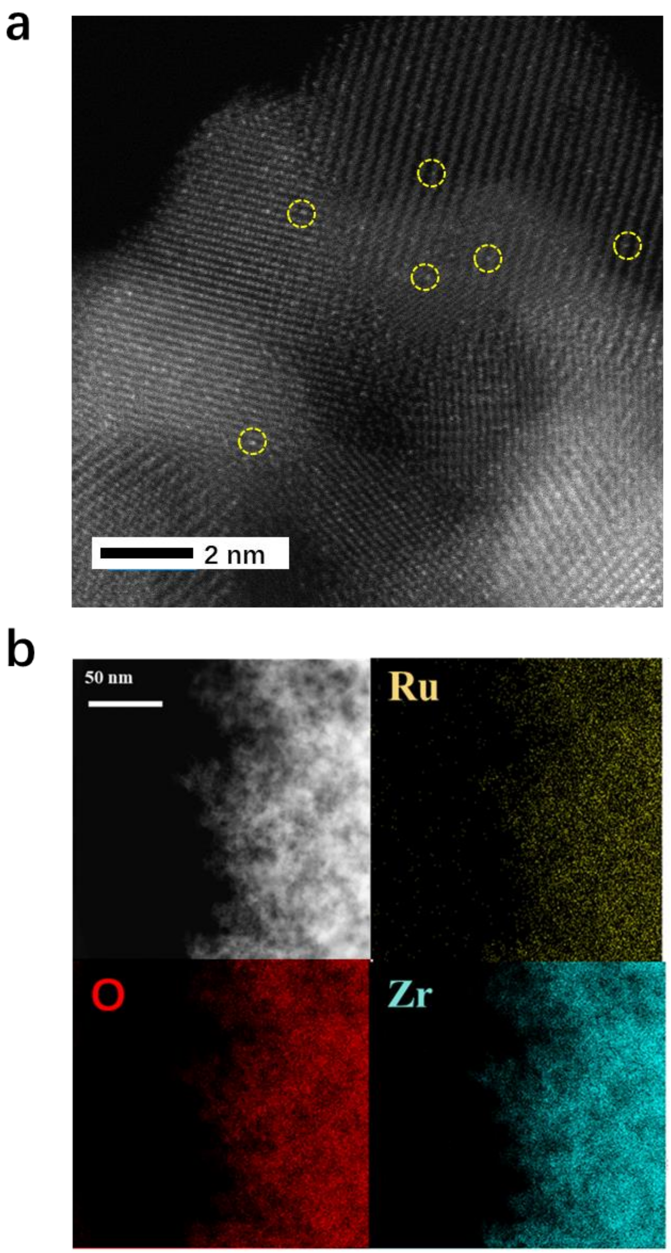

Supplementary Figure 18. (a) HRTEM image and **(b)** EDS element mapping of Ru_1-ZrO_2_500.

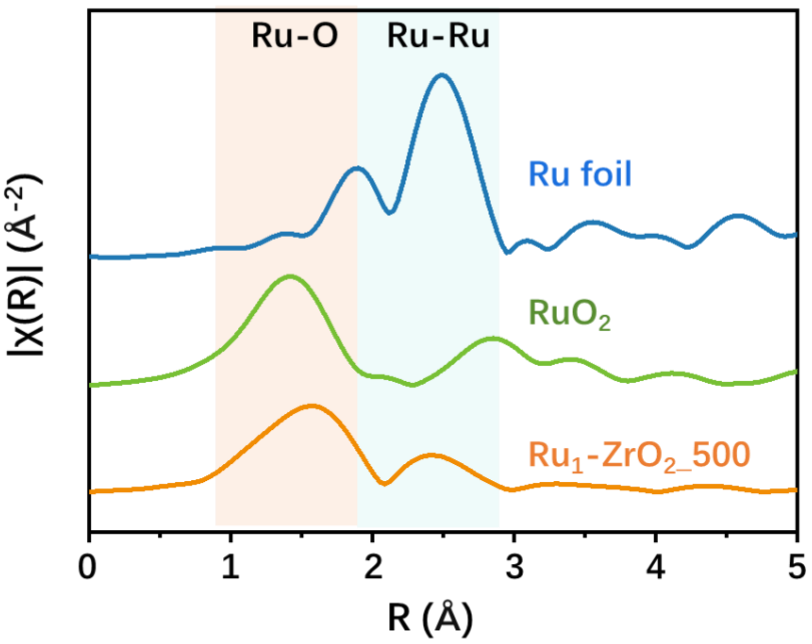

Supplementary Figure19. Fourier transform of Ru EXAFS spectra of Ru_1-ZrO_2_500. Ru foil and RuO_2 were used as references.

A major peak of the Ru-O scattering path at ~1.5 Å in the first coordination sphere was observed for Ru_1-ZrO_2_500.

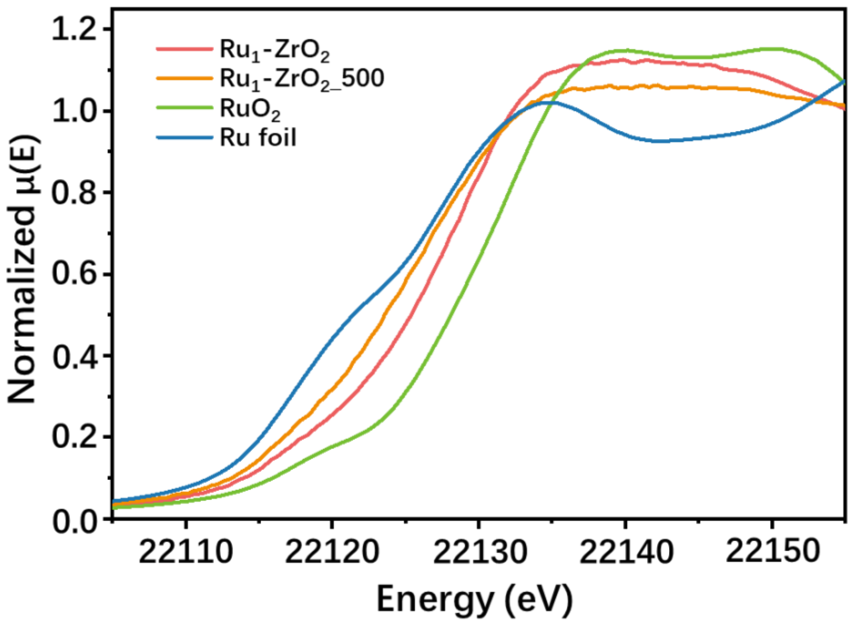

Supplementary Figure 20. Ru *K*-edge XANES spectra of Ru_1-ZrO_2 and Ru_1-ZrO_2_500. Ru foil and RuO_2 were used as references.

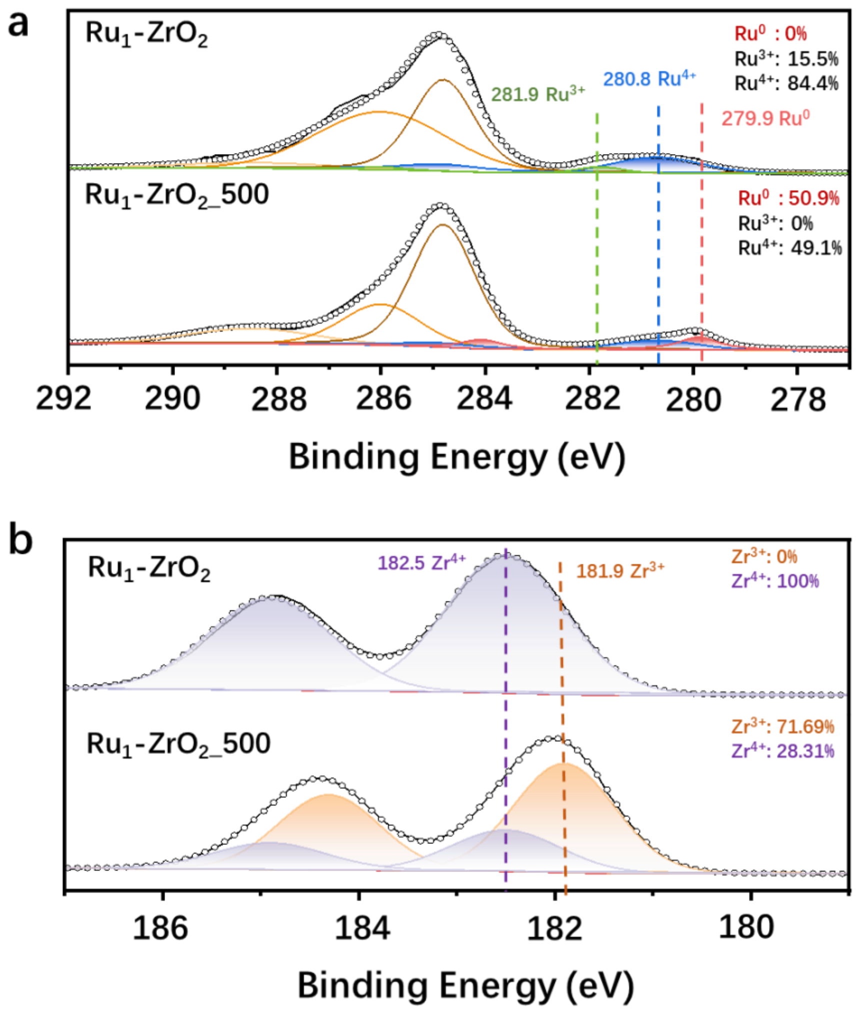

Supplementary Figure 21. (a) Ru 3*d* and **(b)** Zr 3*d* XPS spectra of Ru_1-ZrO_2_500 and Ru_1-ZrO_2.

The XPS results show that Ru exists predominantly in the Ru^{4+}(84.4%) and Ru^{3+}(15.5%) oxidation states in Ru_1-ZrO_2, with no significant presence of metallic Ru^0. These findings align with the XANES data and confirm that Ru remains in an oxidized state. However, in Ru_1-ZrO_2-500, approximately half of the Ru species were reduced to Ru^0. The Zr 3*d* XPS peak around 182 eV exhibits two contributions, Zr^{4+} $3d_{5/2}$ and Zr^{3+} $3d_{5/2}$, located at respectively 182.5 eV and 181.9 eV.[3,4] It is observed that the contribution of Zr^{3+} in Ru_1-ZrO_2_500 is much more significant than that in Ru_1-ZrO_2, indicating a reduction from Zr^{4+} to Zr^{3+} after the H_2 treatment on Ru_1-ZrO_2.

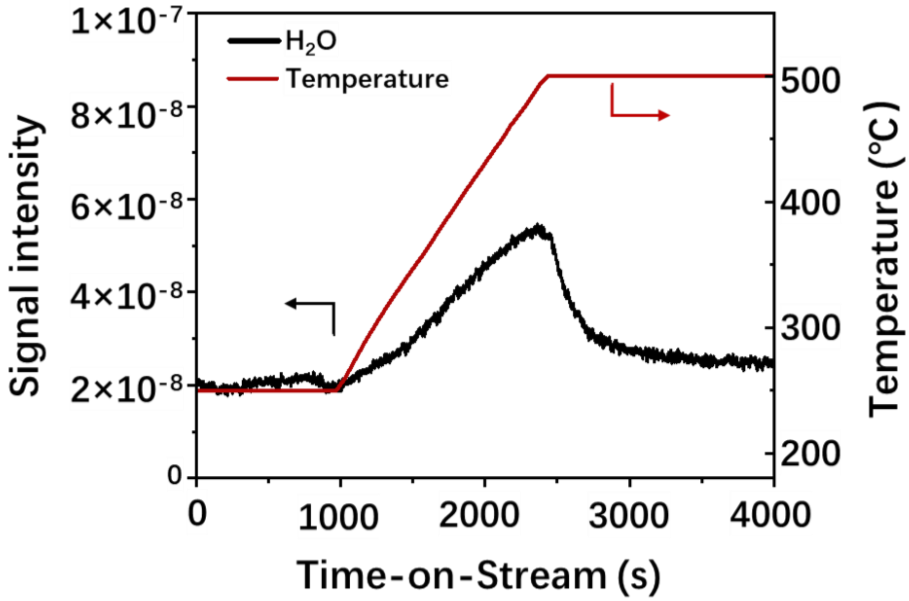

Supplementary Figure 22. H_2-TPR-MS of Ru_1-ZrO_2. The temperature increased from 250 ℃ to 500 ℃ in 10% H_2/Ar with a flow rate of 20 mL/min. The 18 M/Z was recorded to track the H_2O formation in the stream.

The signal of H_2O in the MS with the peak at 500 ℃ aligns well with the H_2-TPR profile using a TCD as the detector in **Fig. 2f**. It demonstrates that the removed atom by H_2 in Ru_1-ZrO_2 is the O atom, and the product is H_2O.

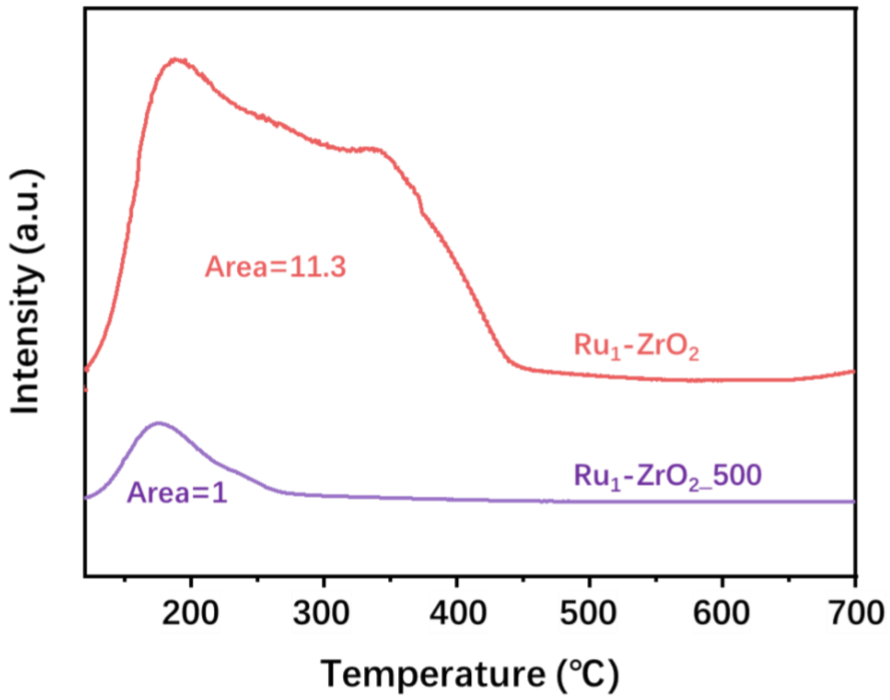

Supplementary Figure 23. NH_3-TPD-MS (M/Z=15) profiles of Ru_1-ZrO_2 and Ru_1-ZrO_2_500.

The desorption of NH_3 temperature is up to 450 ℃ on Ru_1-ZrO_2, which indicates a strong acid site.[5] In contrast, complete NH_3 desorption from Ru_1-ZrO_2_500 was reached at 260°C, indicating the acid site over Ru_1-ZrO_2_500 is weaker compared to that of Ru_1-ZrO_2. Moreover, the integrated intensity of NH_3 desorption peak of Ru_1-ZrO_2 is 11 times higher than that of Ru_1-ZrO_2_500. This result indicates that most of the acid sites disappear after the H_2 treatment at 500 ℃.

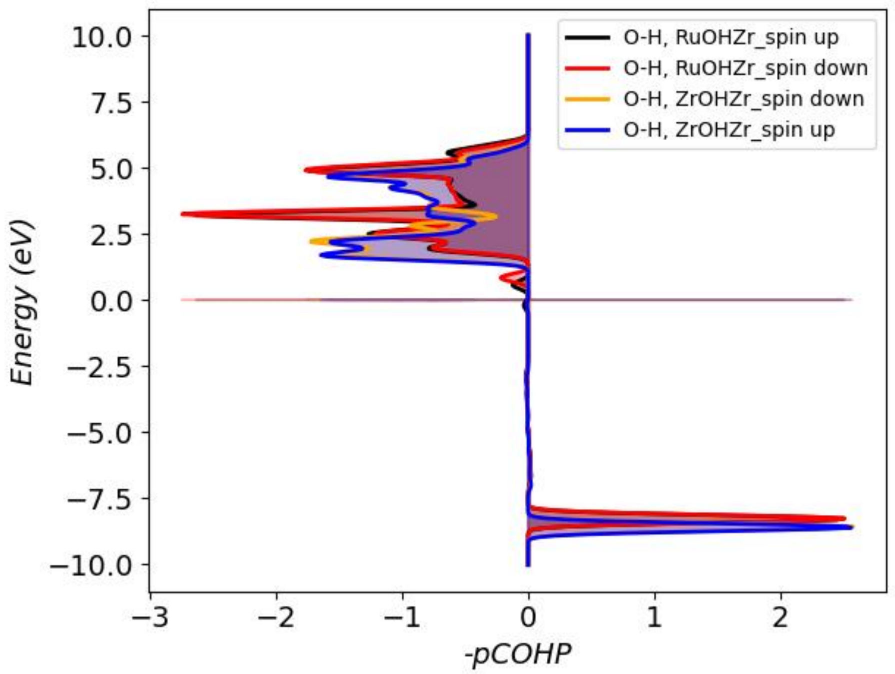

Supplementary Figure 24. The pCOHP plots of the O-H interaction of RuOHZr and ZrOHZr moieties (data obtained from LOBSTER analysis).

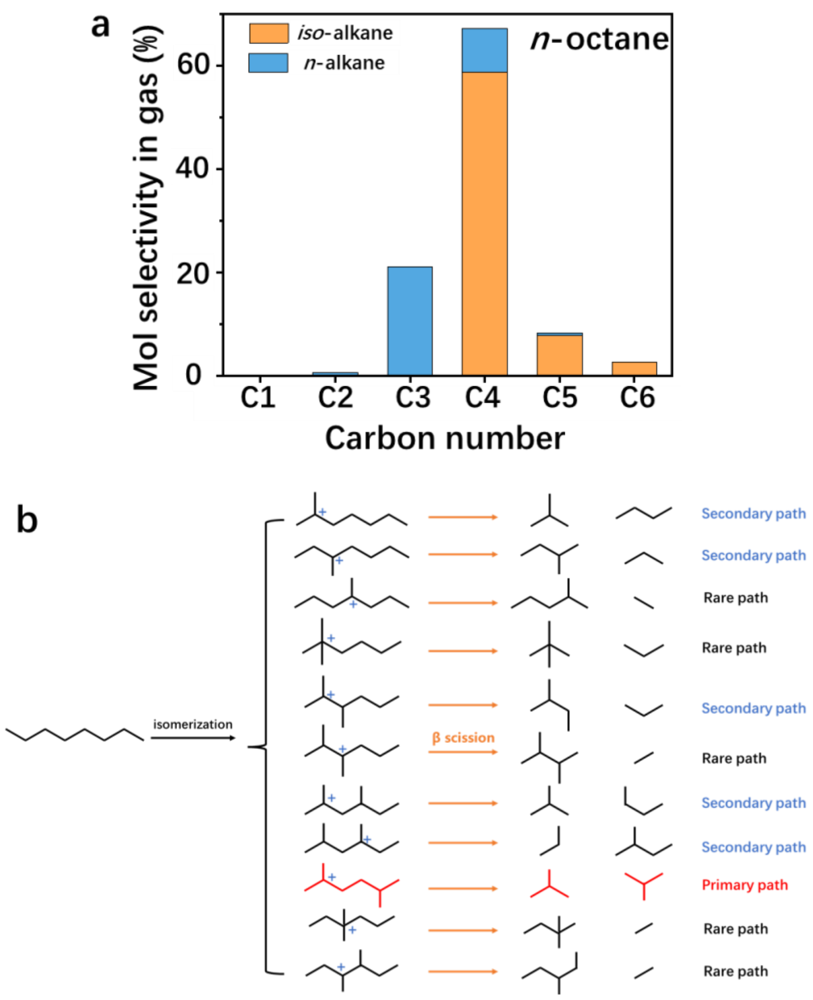

Supplementary Figure 25. (a) Hydrocarbon distribution of gas phase products based on carbon number obtained after the hydrocracking of *n*-octane over Ru_1-ZrO_2 at 250 °C under 3 MPa of H_2 for 12 hours. **(b)** All possible reaction pathways in hydrocracking of *n*-octane.

Supplementary Fig. 25a shows that *iso*-alkane dominates the gas products, so the isomerization of *n*-octane was analyzed and it has a total of 17 isomeric forms. Given the much higher selectivity towards *iso*-butane than all other species, this product can only be achieved via isomerization followed by β-scission (marked in red in **Supplementary Fig. 25b)**). The population of hydrocarbon species, particularly the 60 mol% selectivity for *iso*-butane, indicates the possible primary, secondary and rare pathways in the **Supplementary Fig. 25b**.

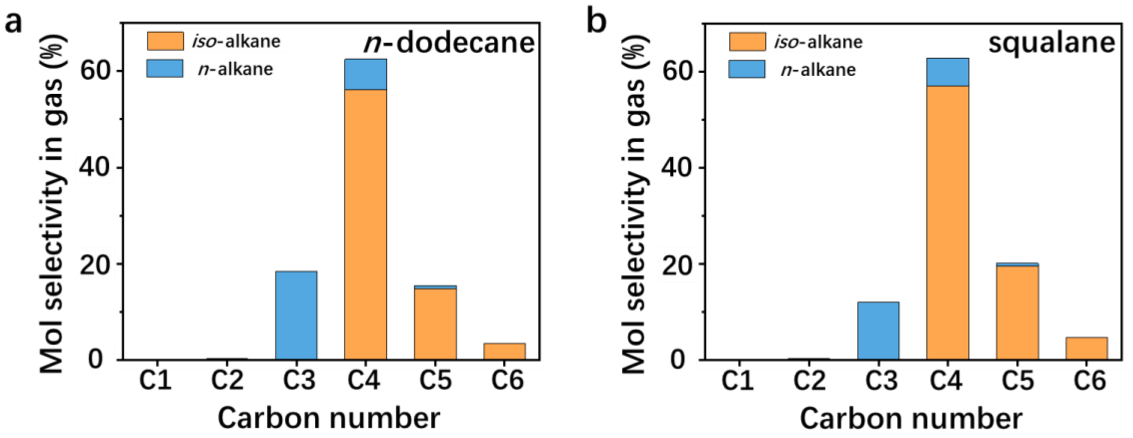

Supplementary Figure 26. Hydrocarbon distribution based on carbon number in the gas products obtained after the hydrocracking of **(a)** *n*-dodecane (C_{12}) and **(b)** squalane (C_{30}) over Ru_1-ZrO_2 at 250 ℃ under 3 MPa of H_2 for 12 hours.

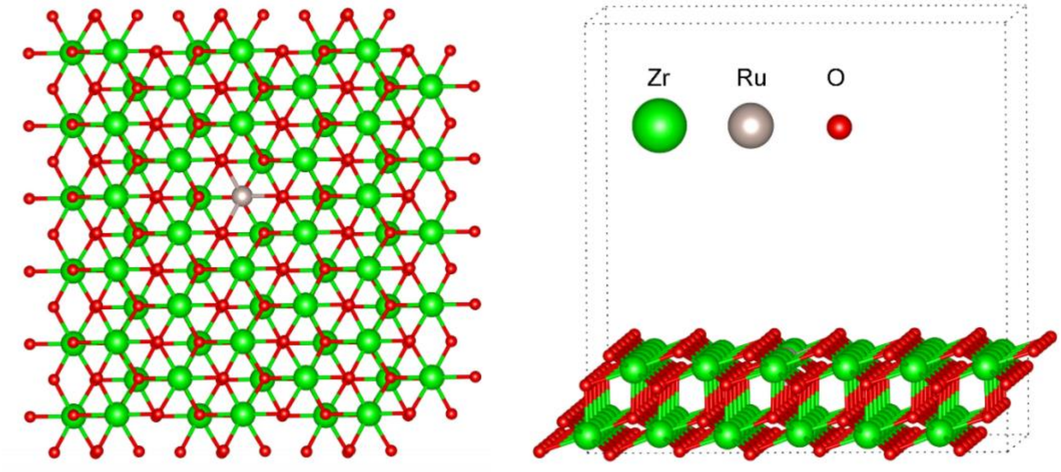

Supplementary Figure 27. Ru_1-ZrO_2 surface model used for DFT calculation (side view and top view).

Density functional theory (DFT) calculations were performed to rationalize the nature of the active site and the reaction mechanism of polypropylene hydrocracking to gas products. The PP substrate was represented by a saturated C_{11} model compound.

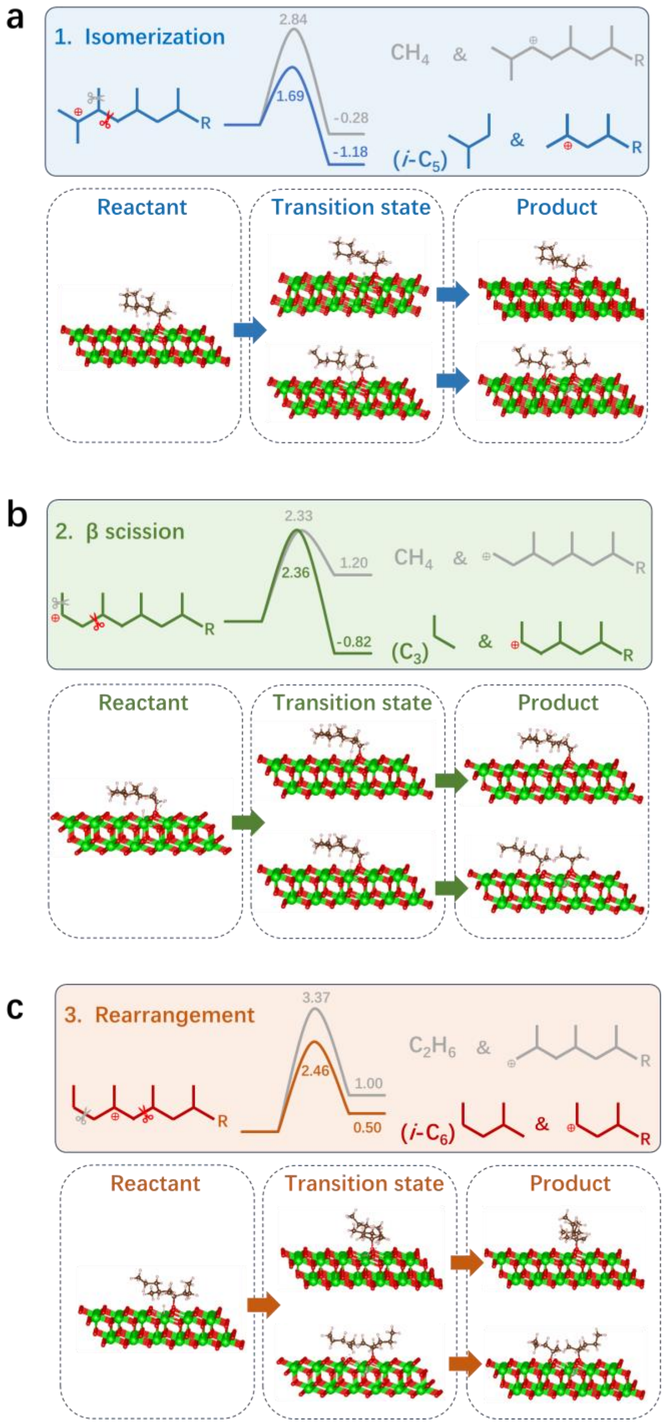

Supplementary Figure 28. DFT calculation optimized reaction intermediates and transition states of C-C bond-breaking reactions in the three routes, **(a)** isomerization, **(b)** β-scission and **(c)** rearrangement.

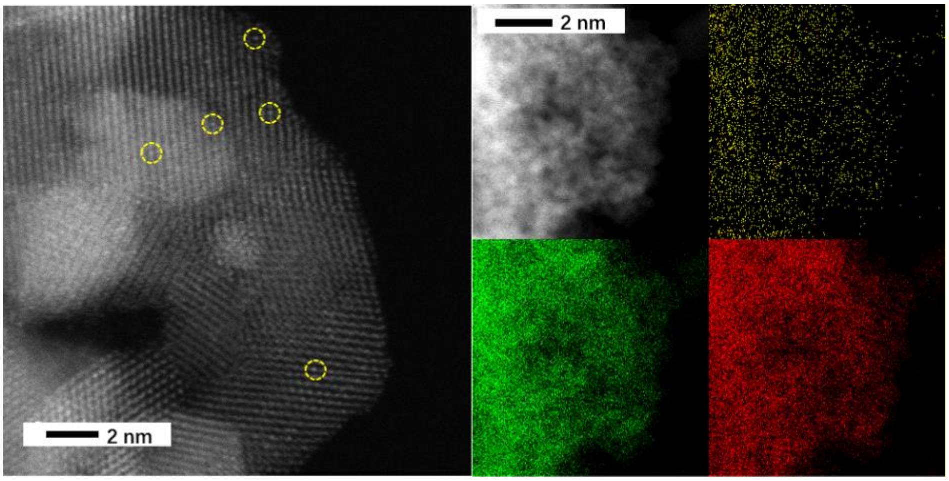

Supplementary Figure 29. HRTEM and EDS element mapping images of used-Ru_1-ZrO_2 at 300 ℃ under 3 MPa of H_2 for 8 hours.

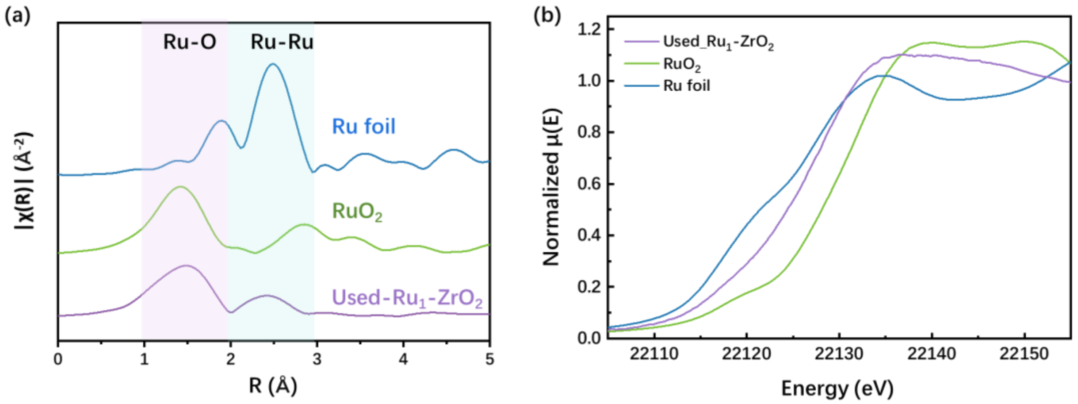

Supplementary Figure 30. Fourier transform of Ru EXAFS and Ru K-edge XANES spectra of used-Ru_1-ZrO_2 at 300 °C under 3 MPa of H_2 for 8 hours**.**

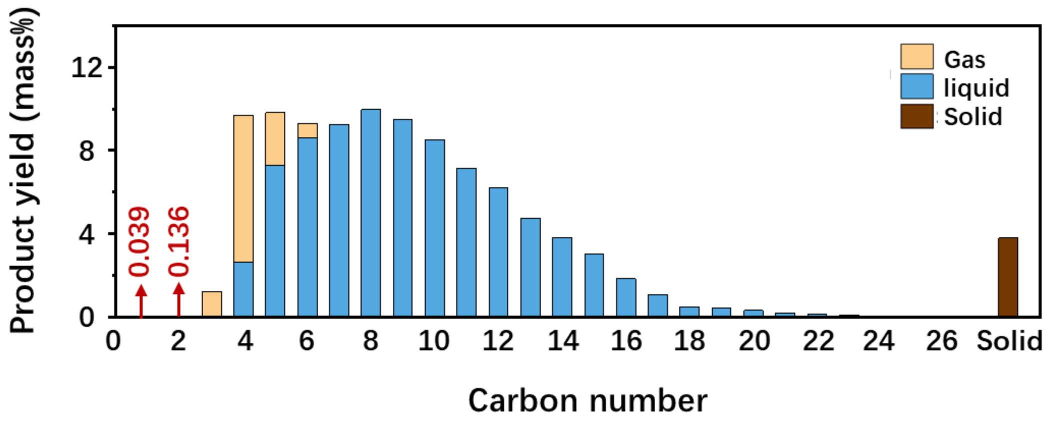

Supplementary Figure 31. Hydrocarbon distribution based on carbon number after hydrocracking of 100 g mixed postconsumer PP and PE wastes over Ru_1-ZrO_2 at 300 ℃ under 3 MPa of H_2 for 8 hours.

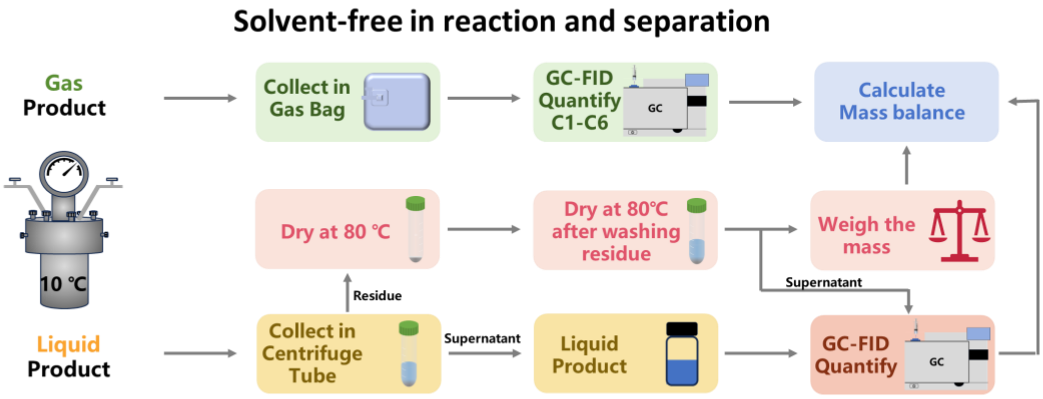

Supplementary Figure 32. Scheme for gas and liquid hydrocarbon product quantification.

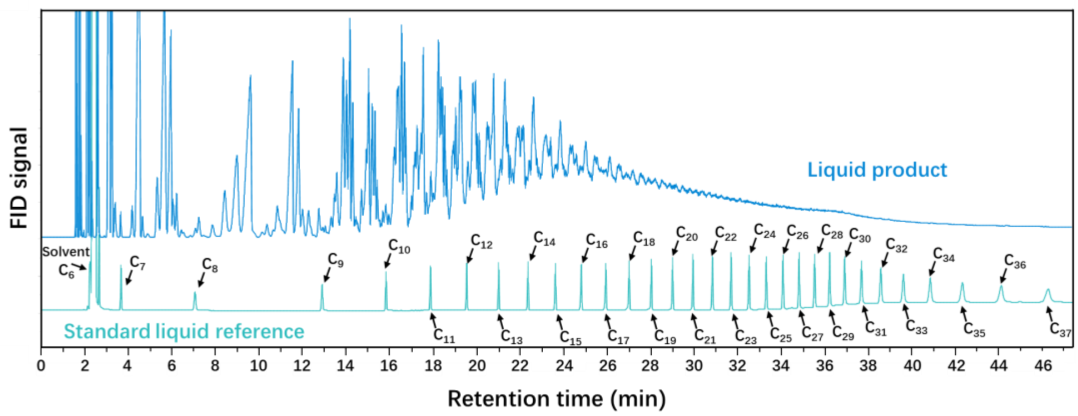

Supplementary Figure 33. GC chromatogram of liquid products from hydrocracking of PP over Ru_1-ZrO_2 at 250 ℃ under 3 MPa of H_2 for 8 hours.

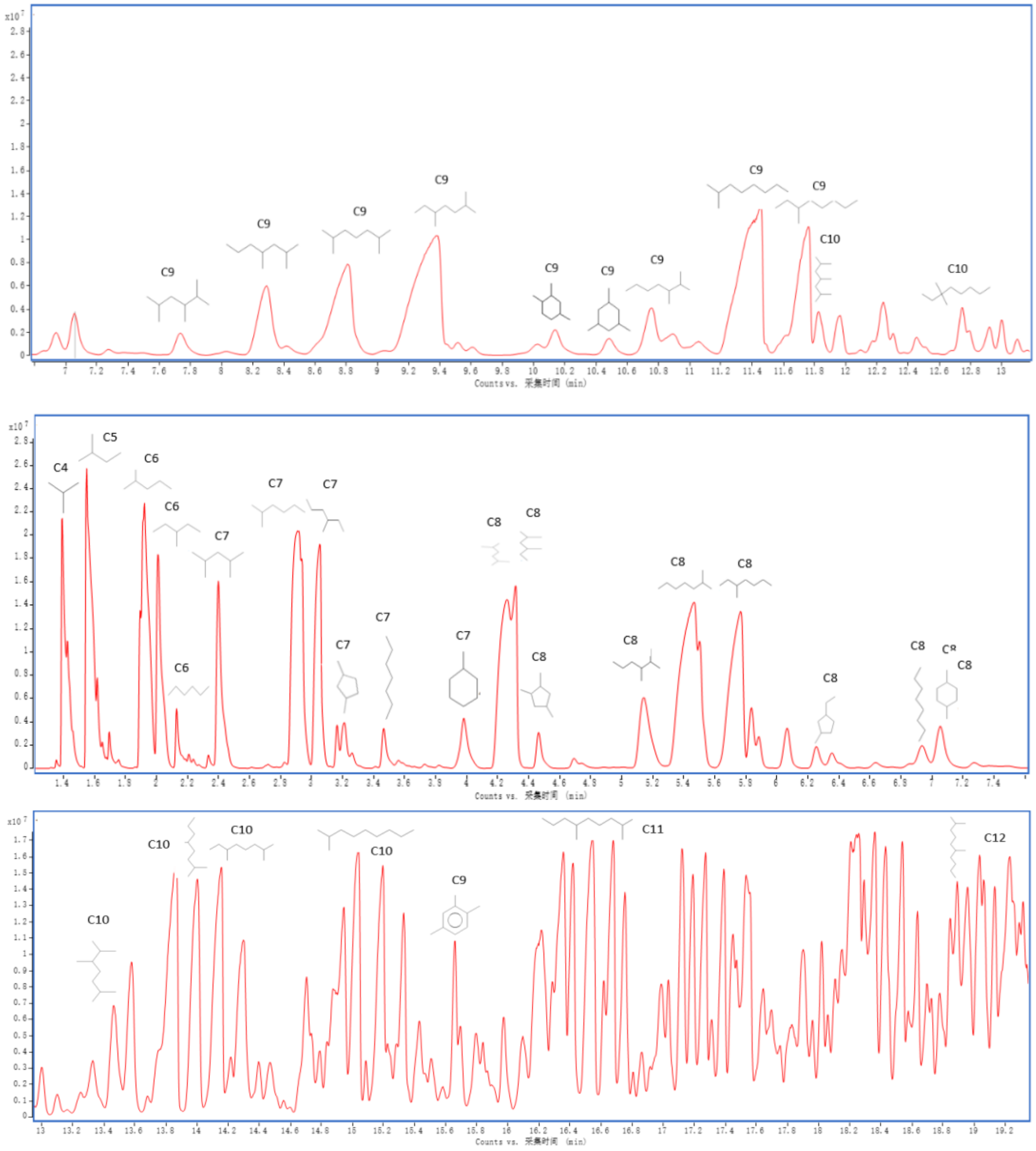

Supplementary Figure 34. GC-MS chromatogram of products from the reaction of PP over Ru_1-ZrO_2 at 250 ℃ under 3 MPa of H_2 for 8 hours.

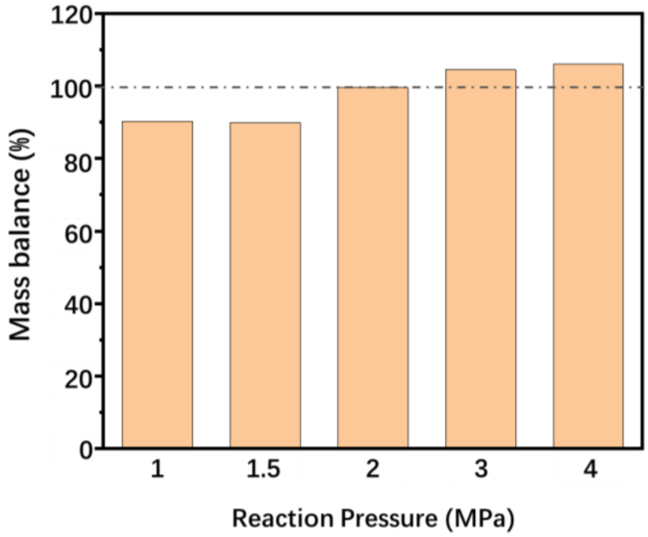

Supplementary Figure 35. Mass balance examination for the products obtained from PP hydrocracking over Ru_1-ZrO_2 at 300 °C under 1-4 MPa of H_2 for 8 hours.

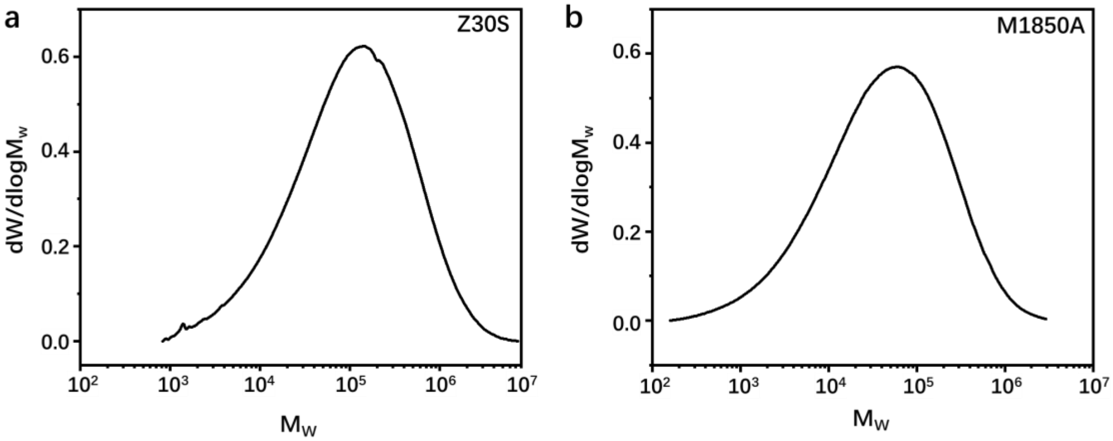

Supplementary Figure 36. GPC test results of **(a)** PP Z30S and **(b)** PE M1850A.

Supplementary References

1 Chen, L. X. *et al.* Disordered, Sub-Nanometer Ru Structures on CeO_2 are Highly Efficient and Selective Catalysts in Polymer Upcycling by Hydrogenolysis. *ACS Catal.* **12**, 4618-4627, (2022).

2 Xin, H. *et al.* Overturning CO_2 Hydrogenation Selectivity with High Activity via Reaction-Induced Strong Metal–Support Interactions. *J. Am. Chem. Soc.* **144**, 4874-4882, (2022).

3 Xiao, T. C., Liu, X. H., Xu, G. Y. & Zhang, Y. Phase tuning of ZrO_2 supported cobalt catalysts for hydrodeoxygenation of 5-hydroxymethylfurfural to 2,5-dimethylfuran under mild conditions. *Appl. Catal. B* **295**, 120270, (2021).

4 Ren, Z. *et al.* Pt/ZrO_2 catalyst with metal-support synergistic effect towards glycerol selective oxidation. *Chem. Eng. J.* **468**, 143623, (2023).

5 Zhou, Q. M. *et al.* Mechanistic Understanding of Efficient Polyethylene Hydrocracking over Two-Dimensional Platinum-Anchored Tungsten Trioxide. *Angew. Chem. Int. Ed.* **62**, e202305644, (2023).
