# Supplementary material for: Upcycling polyolefins to methane-free liquid fuel by a Ru1-ZrO2 catalyst
Source: Nat Commun. 2025 Mar 21;16:2800. doi: 10.1038/s41467-025-57998-x (PMC11928669; doi:10.1038/s41467-025-57998-x)
Supplement: Supplementary file 2 — Transparent Peer Review file [file 41467_2025_57998_MOESM2_ESM.pdf]

nature portfolio

Peer Review File

Upcycling polyolefins to methane-free liquid fuel by a Ru1-ZrO2 catalyst

Corresponding Author: Professor Xiang Wang

This file contains all reviewer reports in order by version, followed by all author rebuttals in order by version.

Version 0:

Reviewer comments:

Reviewer #1

(Remarks to the Author)
This work highlights the application prospect of upcycling polyolefins to methane-free jet fuel using single-atom Ru species supported on ZrO2, with 85 mL liquid obtained from 100 g mixed polyolefins. The mixture of PE and PP, rather than purified ones, is the most possible to exist in daily life, as this work directly targeted on real-life waste. This result is rather innovative for its processing capability, low need for hydrogen or success in enlargement practice, which transforms mixed polyolefin waste into high-value gas and fuel. While utilizing metal most efficiently as single atom species, this work designates certain Bronsted acid site of Ru-O-Zr, which involves a rather controllable synthesis strategy. Furthermore, this work, based on previous work about the single atom catalysts and polyolefin conversion, has shown a rather clear mechanism and achieved a breakthrough, leading the novel pathway of polyolefin conversion between traditional hydrogenolysis and pyrolysis. However, there are some issues required to be investigated and reconsidered in detail:

1. Actually, we see that the process is distinguished from traditional hydrogenolysis and pyrolysis, with inadequate hydrogen for hydrogenolysis. The Fig. 1c showed that lower hydrogen pressure results in a colored product, which might implies the unsaturated bond in the product. Therefore, what ratio of the olefin-like product to the saturated ones? I think this question is quite critical to fuel quality.
2. Moreover, as a process different from pyrolysis with the utilization of hydrogen, the conversion of hydrogen must be shown along with the ratio between H2 and (CH2)n in the feed under different conditions.
3. 85 mL liquid is a good enough result. Nevertheless, for oil-like liquid, it might be much more appropriate to give the result of liquid product weight. And for application as jet fuel, is it comparable with generic jet fuel? With the product performance like the ratio of olefins to alkanes elaborated as mentioned above, it will be a rather competitive result.

Reviewer #2

(Remarks to the Author)
The authors investigated the hydrogenolysis of PE with an atomic Ru-supported ZrO2 catalyst. In this catalyst system, the formation of methane gas was suppressed, but the yield of the target liquid chemicals is quite low (~70%), and the advantage compared to previous studies is very low. To begin with, the fact that the catalyst is 10 wt% in the reaction system is undesirable, indicating that the catalyst's activity is very low. Although the authors did not compare the activity with the reported ones, it is necessary to compare how active it is. Moreover, the effectiveness of single atom Ru in suppressing gas generation has already been reported (Research, 2023, 6, 0032), so there is little novelty in this catalyst system. The reaction mechanism was explained using PP, but it only discusses the stability of carbocations in the dissociated products, which does not apply to PE. Since methane is also not formed when PE is the substrate, this mechanism will not be appropriate. From the perspective of novelty and impact, I would not recommend this paper for publication in Nature Communications.

Reviewer #3

(Remarks to the Author)
The manuscript presents a significant advancement in plastic waste upcycling by demonstrating a novel catalyst design for the selective hydrocracking of post-consumer PE and PP into high-value liquid fuels. The use of atomic Ru-doped ZrO_2 as a

catalyst marks a paradigm shift from traditional supported-metal catalysts to oxide-based mediation, enabling efficient conversion with high selectivity towards jet-fuel-range branched hydrocarbons and the suppression of undesired methane and ethane formation. The manuscript is well organized and is recommended for publication after the revisions noted below.

1) In Figure S13, the amount of H2 consumed was evaluated. What is the ratio of Ru/H? The ratio is over 1? Then, multiple B-acid sites are generated by a single Ru site.
2) The activation of molecular H2 is in a heterotic manner; it means hydride species should be formed at the same time as proton formation. Is there any observation of hydride species using the spectroscopic technique?
3) The theoretical characterization of the B-acid site next to the Ru site is poor. The authors are strongly recommended for additional calculation on the only ZrO2 surface. Is there any difference in the character of B-acid with/without neighboring Ru site (Charge, energy level of orbital, or acidity)?

Reviewer #4

(Remarks to the Author)
The manuscript „Upcycling polyolefins to methane-free jet fuel by a Ru1-ZrO2 catalyst" submitted by Yan et al. describes promising results in the utilization of PP and PE plastic wastes as a resource for fuels. They use a tailored catalyst where Ru is integrated in the ZrO2 structure for the hydrotreating of PP and PE towards olefine fractions in the range for jet fuel applications. One challenge is the loss of carbon due to formation of gaseous products mainly such as Methane and ethane. With the presented catalyst they can minimize methane and ethane formation. Within the manuscript it is speculated that Ru-O-Zr bridges are the active site and a reaction mechanism is presented. The topic is of high interest as well a very up to date. Even though it is a very comprehensive study combining catalyst testing, characterisation, DFT calculations and reference testing the study and the manuscript can be improved in some points as described below:

- Jet fuel is not only the right fraction of C-chain length but has specific requirements such as melting point, heating value, cloud points, density, viscosity etc. Do the obtained Liquid fraction fullfill the specifications to be truly called jet fuel? Otherwise I suggest to change the title and the referencing to the liquid products and not call it jet fuel directly.
- In the abstract: Please add that you use hydrotreating as the technique.
- It is stated: "Converting waste PO to sustainable liquid fuels presents a high potential to partially replace petroleum-route fuels and reduce global net carbon emissions." This is not fully true. If one uses fossil based plastics to produce fuels, the fuel is still fossil based. Please rephrase to what is really meant   reducing plastic waste.
- The literature review should be extended, to that other ways of chemical plastic recycling are available, what are the drawbacks, C fractions formed under what conditions. Furthermore, the review about potential catalysts for hydrotreating and what has been done in literature so far should be improved. It should be specified, what milder conditions exactly means. What are the best results obtained so far in literature?
- There is nowhere written why the specific combination of Ru and ZrO2 has been chosen as the catalyst. The argument that Ru is "a well known methanation metal typically favouring methane formation during hydrocracking" is counter intuitive, why Ru is chosen.
- The Caption of figure 1 needs to be improved. It is not clear from the caption of the presented graphs belong to hydrocracking of PE or PP
- In the results section the dependency of temperature, pressure and reaction time is discussed but without showing any experimental evidence, that these trends hold true for their own catalyst.
- It is stated that a proof test over Ru1-ZrO2 under conditions favourable for methane production was conducted, but the conclusion from this test is missing.
- In general I wonder if the presented results have been repeated at least once. No error bars are shown. Especially the stronger decrease of pressure at higher operation pressure could be also due to slight non-tightness of the reactors instead of more formation of aromatics.
- In the identification of the active site in the XRD where would reflexes of RuO2 be expected? What are the small reflexes at ~15 and 27°?
- In general all provides analytics test the catalyst before the catalytic reaction. I wonder if the atomic dispersion of Ru maintains after the catalyst run and wonder how the surface properties change during catalysis. Especially 40MPa at 300°C is much harsher to the catalyst than the provided TPR under atmospheric pressure. A proof that the catalyst withstands full reduction under operation conditions is needed. In general I suggest to support the results with characterization of the catalyst after the catalytic runs.
- A test of Ru1-ZrO2_500 loss of PP activity is described, but only proven by a picture of the material after reaction. This is not a strong evidence and conversion rates should be provided.
- EDX of Ru1-ZrO2: how long was the EDX collected? The Ru detection looks like a typical scattering image what would be obtained even if no Ru is present.
- The XANES data show no full reduction of Ru. What is the oxidation state of Ru? To what fraction is the Ru in what oxidation state? LCF or similar should be performed.
- Why is the performance of Ru1-ZrO2-500 so much different to Ru/ZrO2?
- Where the DFT calculation really done with PP? Or war as any model compound used?
- The Mechanism in Figure 3a provide details for the formation of C3, C5, and C6 compounds. But the average from the catalytic resulst is C8. How can the tendency for C8 be explained?
- Regarding the general Mechanism of olefin hydrotreating a lot of literature is available (especially focusing hydrotreating of Fischer-Tropsch) which is reaction wise very similar to Polyolefin-Hydrotreating. These literature should be referred to. Given that knowledge the provided mechanism is not very new. Additionally since no operando surface analytics such as DRIFTS or Raman is provided the given mechanism is faily speculative. Within the manuscript no direct proof for the

adsorption of the carbonium ion on the O atom is given. If no surface characterization is available, kinetic investigations together with microkinetic modelling could help to clarify and support the proposed reaction mechanism.

Version 1:

Reviewer comments:

Reviewer #1

(Remarks to the Author)
All comments were addressed and the manuscript has been comprehensively improved. Acceptance is suggested.

Reviewer #3

(Remarks to the Author)
After reviewing the revised manuscript, I am pleased to recommend it for acceptance. The authors have thoroughly addressed all the concerns raised during the initial review process. The revisions have significantly improved the clarity and quality of the work, and the manuscript now presents a comprehensive and well-supported study on the topic. I believe this paper will make a valuable contribution to the field and will be of great interest to the readership of the journal.

Reviewer #4

(Remarks to the Author)
Thank you for your detailed rebutal. My remarks have been addressed.

Open Access This Peer Review File is licensed under a Creative Commons Attribution 4.0 International License, which permits use, sharing, adaptation, distribution and reproduction in any medium or format, as long as you give appropriate credit to the original author(s) and the source, provide a link to the Creative Commons license, and indicate if changes were made.
In cases where reviewers are anonymous, credit should be given to 'Anonymous Referee' and the source.
The images or other third party material in this Peer Review File are included in the article's Creative Commons license, unless indicated otherwise in a credit line to the material. If material is not included in the article's Creative Commons license and your intended use is not permitted by statutory regulation or exceeds the permitted use, you will need to obtain permission directly from the copyright holder.
To view a copy of this license, visit https://creativecommons.org/licenses/by/4.0/

RESPONSES TO THE REVIEWERS

Reviewer 1: This work highlights the application prospect of upcycling polyolefins to methane-free jet fuel using single-atom Ru species supported on ZrO_2, with 85 mL liquid obtained from 100 g mixed polyolefins. The mixture of PE and PP, rather than purified ones, is the most possible to exist in daily life, as this work directly targeted on real-life waste. This result is rather innovative for its processing capability, low need for hydrogen or success in enlargement practice, which transforms mixed polyolefin waste into high-value gas and fuel. While utilizing metal most efficiently as single atom species, this work designates certain Bronsted acid site of Ru-O-Zr, which involves a rather controllable synthesis strategy. Furthermore, this work, based on previous work about the single atom catalysts and polyolefin conversion, has shown a rather clear mechanism and achieved a breakthrough, leading the novel pathway of polyolefin conversion between traditional hydrogenolysis and pyrolysis. However, there are some issues required to be investigated and reconsidered in detail:

1. Actually, we see that the process is distinguished from traditional hydrogenolysis and pyrolysis, with inadequate hydrogen for hydrogenolysis. The Fig. 1c showed that lower hydrogen pressure results in a colored product, which might implies the unsaturated bond in the product. Therefore, what ratio of the olefin-like product to the saturated ones? I think this question is quite critical to fuel quality.

Response:

Thank you for highlighting this important point. We fully agree with your observation that the process differs from traditional hydrogenolysis and pyrolysis. We also believe the color of the liquid product is caused by the unsaturated hydrocarbons. To further quantify the unsaturated hydrocarbons, including the olefin-like product, we re-analyzed the results of ^{1}H NMR for the products obtained under different reaction pressures, which was shown in **Supplementary Figure 8.** Generally, for species related to unsaturated hydrocarbons, hydrogen chemical shifts at 5 - 6 ppm are typically attributed to alkenes (Science, 2022, 375, 1021 – 1024), while those at 6.5 - 9 ppm (Science, 2020, 370, 437 – 441) are associated with aromatic compounds. Based on the observation of the zoom-in region of these two regions (shown below in **Fig. RS1** for your reference), only signals associated with aromatic compounds at 6.5 - 9 ppm are observed, and no peaks corresponding to olefin-like products are observed, which could be due to its low concentration, and the further dilution with CD_2Cl_2 for NMR characterization make it more difficult to observe. Hence, the unsaturated hydrocarbons in the product are primarily aromatics, but they are less than 1%, which has been shown in Figure 1f. This point raised by the reviewer is very important, so we have added the following discussion to **Supplementary Figure 8**:

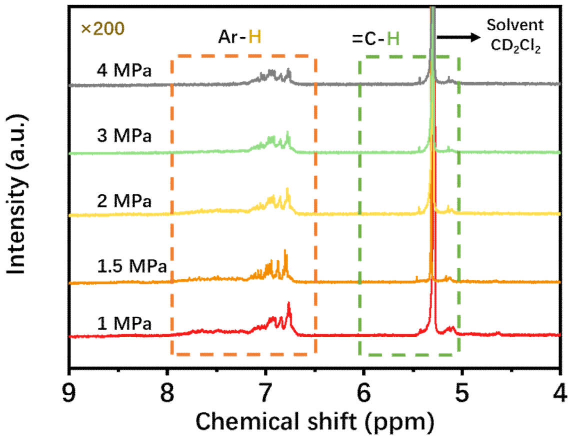

Figure RS1. The enlarged range from 4 - 9 ppm of **Supplementary Figure 8**

Changes made in yellow below Supplementary Information (Page 19)

The appearance of ^{1}H nuclear magnetic resonance (NMR) signals in the regions of 6.5-9.0 ppm and 2.0-3.0 ppm of the liquid is due to the presence of aromatics. However, in the 5-6 ppm region, only signals corresponding to the solvent CD_2Cl_2 were observed. Other unsaturated olefins were not detected due to their low concentration. The relative amount of aromatics was estimated by using the ratio of the integration intensity of aromatics and the integration intensity of all species detected by ^{1}H NMR.

2. Moreover, as a process different from pyrolysis with the utilization of hydrogen, the conversion of hydrogen must be shown along with the ratio between H2 and (CH2)n in the feed under different conditions.

Response:

Thank you for your insightful comment. We have analyzed the hydrogen conversion using the pressure drop of H_2 after the reaction for the reactions performed under different hydrogen pressures (1 MPa, 1.5 MPa, 2 MPa, 3 MPa, 4 MPa) with a consistent substrate weight, i.e. different H_2/substrate mass ratios. The results have been added to the updated **Supplementary Figure 6** (upper x-axis and right y-axis). As the amount or the pressure of H_2 increases (while maintaining a constant substrate mass), the H_2 conversion gradually decreases, but the consumption of H_2 increases. This indicates that the polyolefin cracking reaction is susceptible to the availability of hydrogen. The data and discussion have been added to the Supplementary Information.

Changes made in yellow to the Supplementary Information (Page 17)

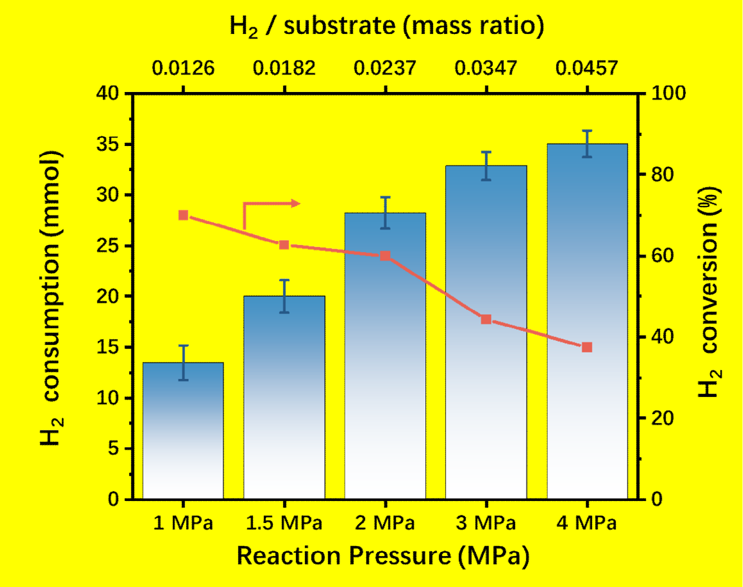

Supplementary Figure 6. Consumption and conversion of H_2 after 8 hours of hydrocracking PP over Ru_1-ZrO_2 at 300 °C under 1-4 MPa of H_2.

The consumption of H_2 was calculated by analyzing the gas products combined with the measured volume of the reactor (See Product Analysis in the Method section). As the amount or the pressure of H_2 increases (while maintaining a constant substrate mass), the H_2 conversion gradually decreases, but the consumption of H_2 increases. Given that the alkanes dominate the product hydrocarbons, one C-C bond cleavage consumes one H_2 molecule. Therefore, the high H_2 consumption indicates more cleavages and the higher pressure of H_2 results in more C-C bonds being cleaved.

3. 85 mL liquid is a good enough result. Nevertheless, for oil-like liquid, it might be much more appropriate to give the result of liquid product weight. And for application as jet fuel, is it comparable with generic jet fuel? With the product performance like the ratio of olefins to alkanes elaborated as mentioned above, it will be a rather competitive result.

Response:

Thank you for pointing out this important aspect. The weight of the 85 mL oil shown in the bottle is about 71g, which was directly collected by centrifuging. However, the total liquid product weight calculated using the method we present in our manuscript is about 82 g, which includes the 71g oil collected by centrifuging and 12g residual calculated using the math after drying the post-reaction catalyst.

Regarding the comparison of the product with commercial jet fuel, we referred to some jet fuel specifications (USAF MIL-T-5624 and GB 6537-2018). Generally, jet fuel primarily consists of alkanes in the C8-C16 range, with aromatics ≤20%, olefins ≤5%. Additionally, certain additives are required to meet performance standards. Our product mainly consists of C8-C16 alkanes with little unsaturated hydrocarbon, which is not a commercial jet fuel. Given jet fuel is not synthesized through a single-step process but rather through blending, our product can serve as a potential feedstock for jet fuel production. This is why we use the term “jet-fuel-ranged branched hydrocarbons” in the abstract of our manuscript. To avoid ambiguity, we have re-checked the whole manuscript and revised instances where only "jet fuel" was mentioned.

Changes made in yellow to the Manuscript

Title: “Upcycling polyolefins to methane-free liquid fuel by a Ru_1-ZrO_2 catalyst”

Page 2: This Brønsted acid site, rather than the atomic Ru dopant itself, selectively governs the internal C-C bond cleavage in polyolefins through a carbonium ion mechanism, thereby enhancing the yield of jet-fuel-ranged hydrocarbons and suppressing methane formation.

Page 3: However, these operating optimizations often result in the under-cracking of polyolefins, leading to a broad carbon distribution with low selectivity for jet-fuel-ranged hydrocarbons. To overcome the economic bundle by maximizing jet fuel component yield and minimizing methane production, the fundamental solution still lies in designing an active site capable of selectively breaking the internal C-C bond of PO rather than the terminal C-C bond.

Page 6: To examine the product preference for jet-fuel-ranged hydrocarbons and resistance to methane formation, a proof test over Ru_1-ZrO_2 was conducted under conditions favorable for methane formation.

Page 14: The use of centrifugation to obtain 85 mL of liquid containing 70% jet-fuel-ranged hydrocarbons significantly lowers the cost of post-reaction product separation.

Reviewer 2: The authors investigated the hydrogenolysis of PE with an atomic Ru-supported ZrO_2 catalyst. In this catalyst system, the formation of methane gas was suppressed, but the yield of the target liquid chemicals is quite low (~70%), and the advantage compared to previous studies is very low. To begin with, the fact that the catalyst is 10 wt% in the reaction system is undesirable, indicating that the catalyst's activity is very low. Although the authors did not compare the activity with the reported ones, it is necessary to compare how active it is. Moreover, the effectiveness of single atom Ru in suppressing gas generation has already been reported (Research, 2023, 6, 0032), so there is little novelty in this catalyst system. The reaction mechanism

was explained using PP, but it only discusses the stability of carbocations in the dissociated products, which does not apply to PE. Since methane is also not formed when PE is the substrate, this mechanism will not be appropriate. From the perspective of novelty and impact, I would not recommend this paper for publication in Nature Communications.

Response:

Thank you for your thoughtful and detailed comments. It is appreciated that we can have the opportunity to further elaborate on our work. To facilitate comparing our work with the previous work, we summarized the state-of-the-art studies on Ru-based catalysts for polyolefin hydroconversion published in the past five years in **Table RS1** below for your reference**.**

(1)_Regarding the concern about the low liquid yield, we reported a 70% liquid yield with a narrow product distribution (C5-C20). As shown in **Table RS1**, 14 out of 15 published studies reported liquid yields with the distribution in C5-C18 are below 70% (see entries 2-5, 7-16, published in the ACS Catal. 2021, 11, 8104−8115; Chem. Catalysis. 2023, 3, 100564; Angew. Chem. Int. Ed. 2024, 63, e202317526; J. Am. Chem. Soc. 2024, 146, 7076−7087, etc.). Notably, entry 6 reported a yield of up to 86%, but they did not specify the carbon number distribution. After carefully learning this study, we found they used CH_2Cl_2 as the solvent to solve all products, which cannot distinguish liquid (C5-C18) from wax (C18-C40). Therefore, the 86% yield is the total of liquid (C5-C18) and wax (C18-C40). Therefore, the yield of 70% liquid with all the products falling in C5-C18 is not lower compared to others.

(2)_We agree with your observation that the amount of catalyst used in the reaction can reflect how active the catalyst is. In **Table RS1**, we listed the substrate-to-Ru ratio (Plastic/Metal Ru), reflecting the usage of Ru in converting plastic waste. In our experiments, we used 4g plastic and 0.005g Ru, this ratio of Ru to plastic is 800, which is superior to entries 2–9 in **Table RS1. It is** comparable to entries 10-11 but showed a higher conversion. Although entry 14 reported a higher Plastic/Metal ratio, the reaction time is 18 hours at 260 °C, which is much longer than our reaction time and higher than our reaction temperature. For entry 16, they used a small amount of Ru metal, but due to its very low loading, 0.4 grams of catalyst was required to work for only 1 gram of plastic. Additionally, the support of Ru used in entry 16 is zeolite, which increases the catalyst cost. Therefore, despite the differences in Ru loading and catalyst types across studies, our experiments show higher conversion, demonstrating the high efficiency of our catalyst system.

(3)_About the innovation, we have discussed current strategies for suppressing methane formation with Ru-based catalysts in the introduction. It is commonly believed that the active site to break C-C bonds is Ru, and most studies focus on tuning the state of Ru, such as oxidation state, particle size, geometry, and surface decoration of metal species. We also cited the article you mentioned (Research 2023, 6, 0032), which changes product selectivity by adjusting the size of Ru particles. However, our research reveals a completely different finding. We discovered that the Brønsted acid site is the active site, and Ru does not directly participate in the cleavage of C-C bonds and suppress the methane formation. This represents a fundamentally different approach compared to previous studies and provides a new perspective on the hydrogenation conversion of polyolefins.

(4)_Regarding the reaction mechanism, we found the branched products from PE are identical to those from PP, which was shown in **Supplementary Figure 3.** Therefore, we speculate isomerization happens and the PE molecules will be isomerized to be branched like PP molecules during the process. To further prove our hypothesis, we used linear alkanes, i.e. n-octane and n-dodecane, as model substrates, and found that the products are almost all branched C4-C6 products regardless of reactants. These results further demonstrate that the linear molecule will be isomerized during the hydrocracking. For your convenience, we have re-placed **Supplementary Figure 25. (a) and 26 (a)** below. Therefore, when we visualized the model, we employed a compound (**Fig 3.b**) that incorporates both PP characteristics (methyl groups) and PE characteristics (linear chain structures) to represent alkane molecules. In this proposed mechanism, we also demonstrated that the linear chain segment first undergoes isomerization into a

structure similar to PP before chain cleavage. Thus, our proposed mechanism accounts for the combined behavior of PE and PP. For your convenience, we have shown **Fig 3.b** below for your convenience.

Once again, we are grateful for your thoughtful and constructive comments. We hope that our revisions address your concerns and demonstrate the significance of our findings.

Table RS1. Summary of Ru-catalysts and catalytic performances for polyolefin hydroconversion

Entry	Catalyst	Plastic/ Metal	Time (h)	Conv. (%)	Yield (wt%)						Reference
					Liquid/Wax			Gas	Jet fuel C8-C18	CH_4	
					Total	Liquid C5-C18	Wax C18-C40				
1	Ru_1-ZrO_2	800	8	98	71.5	71.5	0	28	48	0	our work
2	Ru/C	140	16	93	68	~23 (C8-C18)	~45	21.14	~16	~15	ACS Sustain. Chem. Eng. 2021, 9, 11661−11666
3	Ru-TiO_2	400	16	93.6	65.6	~	~	28.2	~	19.2	ACS Catal. 2021, 11, 8104−8115
4	Ru/TiO_2	400	4	100	20.4	8.2	~	80	~	57.9	Chem. Catalysis. 2023, 3, 100564
5	Ru/C	560	16	96	45	~24 (C8-C18)	~21	50 (C1-C7)	~18	~30	JACS Au 2021, 1, 8−12
6	Ru/TiO_2-8	666	12	78	86.4	~	~	23.2	~	18	Nat. Commun. 2022, 13, 5186
7	5 wt % Ru/ZrO_2	680	24	75	~65.7	56 (C5-C21)	~9.7	12	~	~6	Appl. Catal. B-Environ. 2022, 318, 121870
8	Ru-15WZr	400	2	33	~23	~22	~1	~10	~20.85	~7	JACS Au 2021, 1, 1422−1434
9	Ru_1-TiO_2	400	4	100	9.4	<3	~	90.6	~	40.1	Angew. Chem.-Int. Edit. 2024, 63, e202317526
10	Ru/C	1000	12	81.7	68.2	~	~	10.7	~	~	ACS Catal. 2024, 14, 3228−3240
11	Ru/TiO_2-H600	1000	4	~94	85.5	~41	~44.5	8.5	~28	~6.8	Appl. Catal. B-Environ. 2023, 339, 123122
12	Ru/C-EG120	1000	1.5	97.3	~72.02	~45.3	~26.7	8.3	~34.4	~7.4	J. Am. Chem. Soc. 2024, 146, 7076−7087
13	Ru-VZr	1600	2	~83	~68	~	~	~15	~31	~10	Appl. Catal. B-Environ. 2022, 319, 121899
14	0.125 wt % Ru/CeO_2	2000	18	97.9	~69	~	~	~29	~	~11.6	ACS Catal. 2022, 12, 4618−4627
15	0.2 wt % Ru/CeO_2 (SA)	2000	6	98	94.5	~62	~32.5	3	~45	2.2	Research 2023, 6, 0032
16	p-Ru/SBA-15	2500	4	86	~79	~50.8	~28.2	~7	~35.8	~6.5	Angew. Chem. Int. Ed. 2023, 62, e202313174

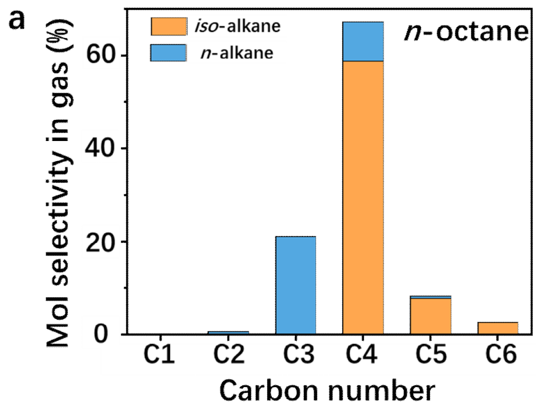

Supplementary Figure 25. (a) Hydrocarbon distribution of gas phase products based on carbon number obtained after the hydrocracking of **n-octane** over Ru_1-ZrO_2 at 250 °C under 3 MPa of H_2

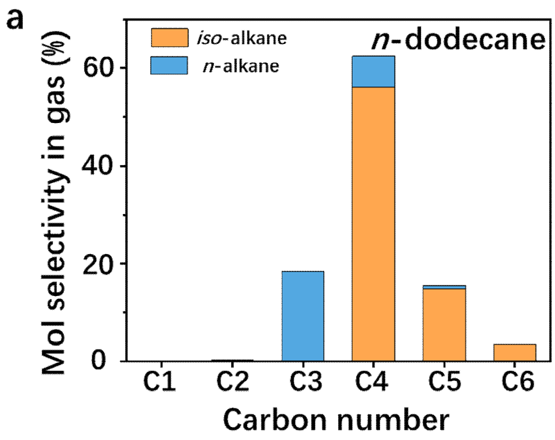

Supplementary Figure 26. (a) Hydrocarbon distribution of gas phase products based on carbon number obtained after the hydrocracking of **n-dodecane** over Ru_1-ZrO_2 at 250 °C under 3 MPa of H_2

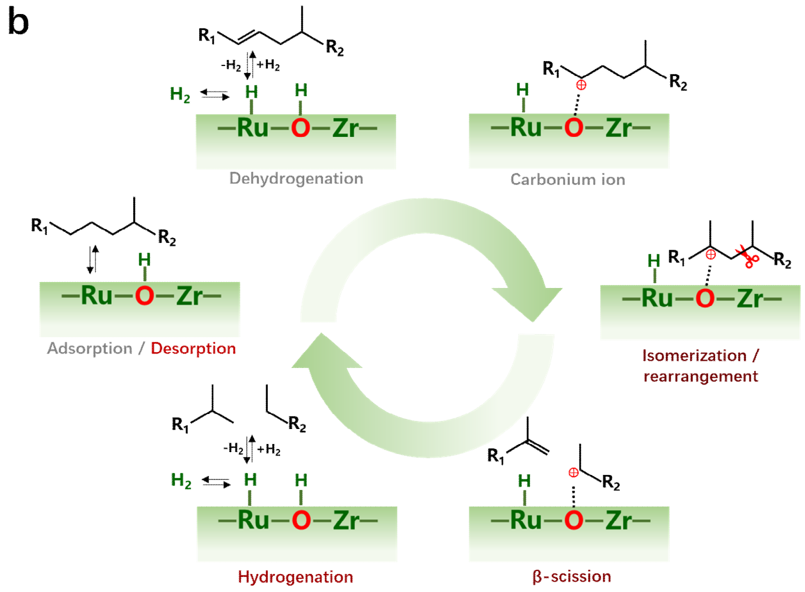

Fig. 3b Schematic catalytic cycle of PO (PE or PP) hydrocracking on Ru_1-ZrO_2.

Reviewer 3: The manuscript presents a significant advancement in plastic waste upcycling by demonstrating a novel catalyst design for the selective hydrocracking of post-consumer PE and PP into high-value liquid fuels. The use of atomic Ru-doped ZrO_2 as a catalyst marks a paradigm shift from traditional supported-metal catalysts to oxide-based mediation, enabling efficient conversion with high selectivity towards jet-fuel-range branched hydrocarbons and the suppression of undesired methane and ethane formation. The manuscript is well organized and is recommended for publication after the revisions noted below.

1) In Figure S13, the amount of H2 consumed was evaluated. What is the ratio of Ru/H? The ratio is over 1? Then, multiple B-acid sites are generated by a single Ru site.

Response:

Thank you for this insightful question. In Figure S13, the amount of H_2 consumed during the H_2-TPR test is approximately 0.25 mmol calibrated using the CuO reduction with H_2, the mass of the Ru_1-ZrO_2 catalyst is 50 mg containing approximately 0.0067 mmol of Ru (based on the loading of Ru at 1.3 wt% calculated XRF analysis). Therefore, the Ru/H molar ratio is 0.0134, less than 1.

We completely agree with your perspective. According to EXAFS data, the coordination number of Ru-O is approximately 4, indicating that one single Ru atom can form more than one Ru-O-Zr moiety, thereby generating multiple Brønsted acid sites. The formation of the multiple Brønsted acid sites is one of the reasons for the largely increased consumption of H_2 on Ru_1-ZrO_2 catalyst compared to that on bare ZrO_2. Another reason is the H spillover caused by the Ru, so that more of ZrO_2 gets reduced.

2) The activation of molecular H2 is in a heterotic manner; it means hydride species should be formed at the same time as proton formation. Is there any observation of hydride species using the spectroscopic technique?

Response:

We sincerely appreciate your suggestion. Following your advice, we conducted the DRIFTS experiment switching the flowing gas from Ar to H_2 over Ru_1-ZrO_2. As shown below in Figure RS2, the peaks at 3771 cm^{-1} and 3674 cm^{-1} grow up as the flowing gas is switched from Ar to H_2. They are attributed to O-H bands of Zr-OH, which indicates that the dissociation of H_2 during the reaction promotes the formation of hydroxyl groups. In the meantime, an indistinct peak at 2088 cm^{-1} can be found. Given the limited studies on Ru-H species, we referred to research on noble metals with similar properties, such as Pd-H (2030 cm^{-1}, J. Phys. Chem. C 2022, 126, 19376−19385) and Pt-H (2110 cm^{-1}, Journal of Catalysis 2023 425 40–49), and speculated that it could be attributed to the band of Ru-H, and its low intensity could be caused by the extremely low concentration of exposed Ru on the Ru_1-ZrO_2 surface, or instability of Ru-H species, or the co-efficiency of Ru-H band in FTIR.

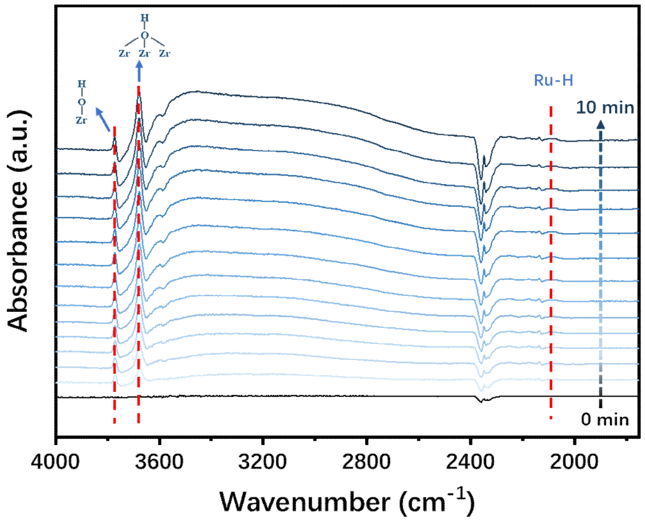

Figure RS2. DRIFTS spectra of Ru_1-ZrO_2 after the feed gas switched from Ar to H_2 with a flow rate of 20 ml/min at 25 ℃

3) The theoretical characterization of the B-acid site next to the Ru site is poor. The authors are strongly recommended for additional calculation on the only ZrO2 surface. Is there any difference in the character of B-acid with/without neighboring Ru site (Charge, energy level of orbital, or acidity)?

Response:

We thank this Referee for the valuable suggestion. We performed further charge and chemical bonding analysis for both RuOHZr and ZrOHZr moieties on the surfaces of Ru-doped ZrO_2 and pure ZrO_2. Bader charge analysis indicates that the H atom of RuOHZr has a net charge of 0.68 |e|, while the Bader charge of the H atom of ZrOHZr is 0.64 |e|. This means that the OH group of RuOHZr is more positively charged and thus, more acidic than that of ZrOHZr. Moreover, we further conducted the crystal orbital Hamilton population (COHP) analysis and added the results to **Supplementary Figure 24**, which shows that both bonding and antibonding O−H interactions of RuOHZr shift to a higher energy level compared to those of ZrOHZr, indicating a relatively weaker O-H interaction and a higher acidity of the OH group of RuOHZr. This is also reflected by the integrated COHP values newly added to **Supplementary Table 5**, which show that the O-H distance of RuOHZr moiety is longer than the *d*(O-H) of ZrOHZr. Additionally, the integrated COHP values of RuOHZr are also closer to the Fermi level, indicating a weaker O-H interaction than that of ZrOHZr. Therefore, it is proposed that the acidity of ZrO_2 is increased by single Ru atom doping which is beneficial for the hydrogenation reaction. We've added the appropriate content to the Manuscript and Supplementary information.

Changes made in yellow to the Manuscript (Page 11)

….. it is concluded that the O atom of the Ru-O-Zr moiety has a dangling H, which acts as the Brønsted acid site.

We further performed charge and chemical bonding analysis for Ru-O-Zr moiety with a dangling H on the Ru-doped ZrO_2. Crystal Orbital Hamilton Population (COHP) analysis (**Supplementary Figure 24)** revealed that, compared to ZrOHZr (assuming it exists), the bonding and antibonding O-H interactions in RuOHZr shifted to higher energy levels. This indicates that the O-H interaction in RuOHZr is relatively weaker, suggesting higher acidity of the OH group. Moreover, the integrated COHP values (**Supplementary Table 5**) show that the O-H bond distance in the RuOHZr moiety is longer than ZrOHZr and is closer to the Fermi level, further suggesting a weaker O-H interaction, i.e. a stonger acidity, in RuOHZr, which is in line with the results of NH_3-TPD.

Changes made in yellow to the Supplementary information (Page 35)

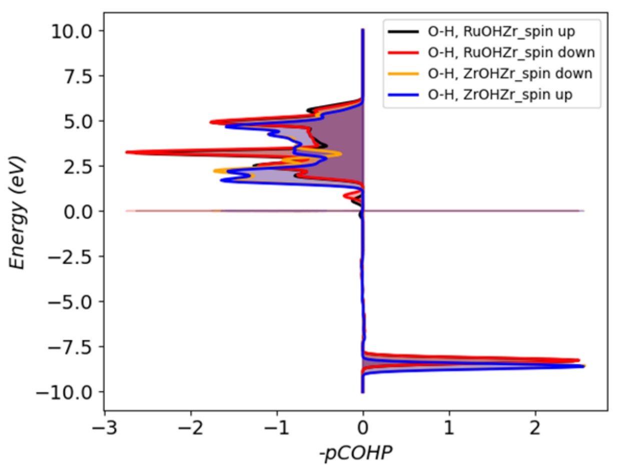

Supplementary Figure 24. The pCOHP plots of the O-H interaction of RuOHZr and ZrOHZr moieties (data obtained from LOBSTER analysis).

Supplementary information (Page 11)

Supplementary Table 5. Integrated values of the spin-polarized COHP denote net bonding characters for the O-H interactions of RuOHZr and ZrOHZr moieties of the surface slab models of Ru_1-ZrO_2 and ZrO_2

COHP	Atom	Atom	Distance *d*(O-H)	ICOHP spin_up	ICOHP spin_down
RuOHZr	O	H	0.97442	-3.86930	-3.88393
ZrOHZr	O	H	0.97404	-3.89581	-3.90427

Reviewer 4: The manuscript „Upcycling polyolefins to methane-free jet fuel by a Ru1-ZrO2 catalyst" submitted by Yan et al. describes promising results in the utilization of PP and PE plastic wastes as a resource for fuels. They use a tailored catalyst where Ru is integrated in the ZrO2 structure for the hydrotreating of PP and PE towards olefine fractions in the range for jet fuel applications. One challenge is the loss of carbon due to formation of gaseous products mainly such as Methane and ethane. With the presented catalyst they can minimize methane and ethane formation. Within the manuscript it is speculated that Ru-O-Zr bridges are the active site and a reaction mechanism is presented. The topic is of high interest as well a very up to date. Even though it is a very comprehensive study combining catalyst testing, characterisation, DFT calculations and reference testing the study and the manuscript can be improved in some points as described below:

1.- Jet fuel is not only the right fraction of C-chain length but has specific requirements such as melting point, heating value, cloud points, density, viscosity etc. Do the obtained Liquid fraction fullfill the specifications to be truly called jet fuel? Otherwise I suggest to change the title and the referencing to the liquid products and not call it jet fuel directly.

Response:

We sincerely appreciate your valuable feedback. As you mentioned, we fully agree that jet fuel has requirements beyond carbon chain length, including other key parameters. Therefore, we have revised the title to "*Upcycling polyolefins to methane-free liquid fuel by a Ru_1-ZrO_2 catalyst.*"

2.- In the abstract: Please add that you use hydrotreating as the technique.

Response:

Thank you for your suggestion. We agree with the importance of clearly stating the technique of hydrotreating used in this study. Actually, in our original abstract, we have used the term "hydrocracking" to indicate that this is a hydrotreating process.

You may check it in the line 3 of the abstract, the sentence starting with " *In this work, we report that atomic Ru-doped ZrO_2 can selectively convert 100 grams of post-consumer polyethylene and polypropylene, yielding 85 mL of liquid in a solvent-free* ***hydrocracking.***"

3.It is stated: "Converting waste PO to sustainable liquid fuels presents a high potential to partially replace petroleum-route fuels and reduce global net carbon emissions." This is not fully true. If one uses fossil based plastics to produce fuels, the fuel is still fossil based. Please rephrase to what is really meant □ reducing plastic waste.

Response:

Thank you for this insightful comment. We agree that most plastics are still derived from fossil resources. However, once the plastic is produced, the fossil has already been explored. If waste plastics (already exploited fossil resources) can be converted to liquid fuel to meet our daily demands for fuels, we don't need to dig for new crude oil. In this way, the technology of converting waste plastics to liquid fuels can contribute to carbon emissions reduction and partially replace petroleum-route fuels. However, an argument does exist, that is whether the plastic oil fuel belongs to the "sustainable" fuel or not. One perspective argues that they are not sustainable, as plastics are primarily made from fossil resources. The other view supports their sustainability, emphasizing that the fuel represents a re-utilization of waste materials. As this topic is relatively new, there is still no agreement on the word "sustainable", so we deleted this word in our sentence and provided the revised content on Page 3 of the manuscript below.

Changes made in yellow to the manuscript (Page 3)

Converting waste PO to liquid fuels presents a high potential to partially replace petroleum-route fuels and reduce global net carbon emissions.

4.- The literature review should be extended, to that other ways of chemical plastic recycling are available, what are the drawbacks, C fractions formed under what conditions. Furthermore, the review about potential catalysts for hydrotreating and what has been done in literature so far should be improved. It should be specified, what milder conditions exactly means. What are the best results obtained so far in literature?

Response:

Thank you for your constructive suggestion. We have expanded the Introduction by including more detailed discussions of other chemical recycling methods and current progress of the hydrotreating catalysts, especially the efforts on suppressing CH_4 formation during the hydrocracking of plastics. As we are focusing on suppressing CH_4 formation while using Ru based catalyst, to the best of our knowledge, M. Chu et al. (Research 2023, 6, 0032) employed an ultra-low loading strategy to prepare a single-atom 0.2 Ru/CeO_2 catalyst, reducing methane yield to approximately 2.2%, representing the lowest CH_4 yield reported to date in Ru-based catalysts, but the liquid product distribution is at C5-C35. The broad distribution of production means the long-chain molecule is still under-cracked when the reaction is ceased. As the reaction time is further extended, the long chain of under-cracked molecules, like C20-C35, will be get shorter and shorter to under C20, but more CH4 yield will be generated. Our results demonstrate that we have reduced the yields of methane and ethane to nearly 0, while keeping the hydrocarbons in a narrow range, i.e. C5-C18. Thank you for raising this question, which helps to further highlight our findings, so we have added the relevant discussions to the introduction of the manuscript and shown it below for your convenience.

Changes made in yellow to the manuscript (Page 3)

…In contrast, hydrocracking with metal/oxide bifunctional catalysts has shown promise in producing high-value fuels, waxes, and lubricants under milder conditions, usually below 300 ℃[16-24]. Nevertheless, achieving high selectivity for liquid fuels, particularly in the jet fuel range (C_8-C_{16}), over metal/oxide catalysts remains challenging.

Among metal/oxide catalysts, Ru-based catalysts have been studied in the hydroconversion of polyolefins due

to their outstanding efficiency in breaking C-C bonds[25]. However, its tendency to generate substantial amounts of low-value methane significantly diminishes the economic viability of the hydrocracking process. Several operating strategies have been developed to suppress methane formation, such as decreasing reaction temperature[26], shortening reaction time, and increasing H_2 pressure[27,28]. However, these operating optimizations often result in the under-cracking of polyolefins, leading to a broad carbon distribution with low selectivity for jet-fuel-ranged hydrocarbons. To overcome the economic bundle by maximizing jet fuel component yield and minimizing methane production, the fundamental solution still lies in designing an active site capable of selectively breaking the internal C-C bond of PO rather than the terminal C-C bond. Efforts have been focused on the manipulation of supported metal species[17], and regulation of the oxidation state[29,30], particle size[21,31-34] and surface decoration[35] of these metal species has proved its effectiveness in reducing undesired methane production. For instance, positively charged Ru species have been reported to favor the cleavage of the internal C−C bond over the terminal C-C bond, thus inhibiting methane formation, but the yield of methane is still at 4.5% with a conversion of PE at 80%[36]. Ru nanoparticles with multiple exposed Ru sites tend to cleave the terminal C–C bond producing methane, and the sub-nanometer particle or single atom of Ru was found effective in suppressing methane formation, but a low methane yield was only obtained in the scenario of under-cracking of PO with a wide distribution of the liquid/wax products up to C_{40} [31,32]. Decoration of 5 wt% Ru/ZrO_2 with 25 wt% WO_3 significantly reduced methane yield from 15% to 5%, but this improvement came at the cost of PO conversion reduced from 75% to 60% [35]. Therefore, the low methane output and the high jet fuel yield still cannot be simultaneously achieved at a high polyolefin conversion, appearing either under-cracking or over-cracking. Still, if the metal site governing the internal C-C bond cleavage is unclear, impeding the rational design of active sites that can selectively promote internal C-C scission while not triggering terminal cascades.

5.There is nowhere written why the specific combination of Ru and ZrO2 has been chosen as the catalyst. The argument that Ru is “a well known methanation metal typically favouring methane formation during hydrocracking” is counter intuitive, why Ru is chosen.

Response:

We appreciate this comment. ZrO_2 is a well-known amphoteric oxide, making it highly versatile for catalytic applications. As reported in the literature (ACS Catal. 2020, 10, 5086−5100), Ru is one of the metals for the fastest cleaving C−C bonds predicted by DFT. Moreover, Ru-based catalysts such as Ru/TiO_2, Ru/CeO_2, and Ru/C were widely studied and found more active than Pt-based catalysts, but they produce significant amounts of methane. Therefore, suppressing methane while keeping the high activity in yielding liquid fuel is a great challenge to move forward in the study of Ru-based catalysts. Combined with the question 4, we have added the justification and more background of Ru-based catalyst and the challenge of suppressing methane to the introduction of the manuscript (refer to the Question 4).

6.- The Caption of figure 1 needs to be improved. It is not clear from the caption of the presented graphs belong to hydrocracking of PE or PP

Response:

Thank you for your reminder. When we prepared the caption, we referred to the author’s guidance of the journal. The caption for Figure 1 was formatted in accordance with the journal's requirements: the general description uses the "a-b," format, followed by specific explanations in the "(a)" format.The plastic in **Fig. 1a** was used PP and PE, while in **Fig. 1b-f** were used PP. Thank you for raising this question, we found the “f” was missing in the “b-e”, so we have made this correction as follows:

Changes made in yellow to the manuscript (Page 5)

Fig. 1 | Solvent-free hydrocracking of PE and PP to methane-free fuels. **a,** The yields of liquid and gaseous products in solvent-free hydrocracking 4 g PE or PP over Ru_1-ZrO_2 at 250 ℃ under 3 MPa H_2 for 8 hours. **b-f,** Hydrocarbon distribution **(b)**, Photo **(c)**, UV- Vis absorption spectra **(d)**, $^{13}C-^{1}H$ HSQC NMR **(e)** and relative amount of aromatics **(f)** calculated based on ^{1}H NMR (**Supplementary Fig. 8**) of the liquid product of hydrocracking PP on Ru_1-ZrO_2 at 300 ℃ under 1.5 MPa H_2 **(e)** and 1-4 MPa H_2 **(f)** for 8 hours.

7- In the results section the dependency of temperature, pressure and reaction time is discussed but without showing any experimental evidence, that these trends hold true for their own catalyst.

Response:

We appreciate the opportunity to further elaborate on our work. In fact, the experimental evidence has already been included. It was initially compiled in Supplementary Figures 1, 4, and 5, Supplementary Table 1, confirming the effects of pressure, temperature, and reaction time on activity and methane yield. We re-showed them below for your reference.

In the manuscript, we have detailed the impact of pressure. As shown in **Supplementary Figure 5** and **Supplementary Table 1**, with the increase in H_2 pressure, the conversion increased from 92% to 100%. At the same time, due to the catalyst's excellent resistance to methane formation. Even though the yields of methane and ethane were very low, they also decreased with increasing H_2 pressure, from 0.214% and 0.483% to 0.025% and 0.016%, respectively, meeting the trend of H_2 pressure impact. Actually, we already showed the evidence and discussed it in our main text on the Page 6. We re-showed it here for your convenience.

"*As previously reported*[4,12,27,33,34]*, the low H_2 pressure and high reaction temperatures favor the formation of methane and ethane. This trend holds true in this proof test (**Supplementary Table 1 lists C_1-C_2 values from Fig. 1b**). However, Ru_1-ZrO_2 still keeps the methane yield below 0.2 wt% while maintaining liquid fuels (C_5-C_{20}) around 70 wt%, even under the reaction condition most greatly favoring methane formation.*"

Regarding the effect of reaction temperature, high temperatures enhance the thermodynamic driving force toward terminal C-C bond cleavage, leading to increased methane production. However, they also shorten the required reaction time, which is often practical in industrial settings. When the temperature was increased from 250°C (**Supplementary Figure 1**) to 300°C (**Supplementary Figure 4**), the reaction time could be reduced to 2 hours to achieve a similar conversion of around 98%, and methane production rose from 0.007% to 0.023%. Our catalyst still meets the trend and keeps the ultra-low yield of methane at high temperatures.

Regarding the reaction-time-led overcracking, when the reaction time was extended from 2 hours to 8 hours at 300°C, there was no significant change in conversion as it was already near 100% after 2 hours. However, due to the selective cleavage of non-terminal C-C bonds by Ru_1-ZrO_2, the methane yield only slightly increased from 0.023% to 0.025%. Our catalyst still meets the trend and demonstrates a superior selectivity in suppressing methane.

We hope these discussions address your concerns.

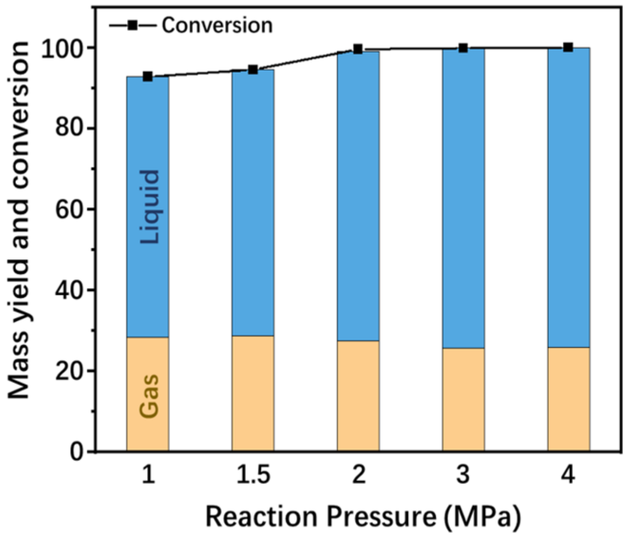

Supplementary Figure 5. The mass yields of gas and liquid products, and the conversion of PP over Ru_1-ZrO_2 after 8 hours at 300 °C under 1-4 MPa of H_2.

Supplementary Table 1. The yields of methane and ethane products at different pressures of H_2 as shown in **Fig. 1b**.

Product	Mass yield %				
	**1 MPa**	**1.5 MPa**	**2 MPa**	**3 MPa**	**4 MPa**
**Methane**	0.214	0.163	0.040	0.025	0.025
**Ethane**	0.483	0.374	0.158	0.117	0.116

Reaction conditions: 300 °C, 8 hours, 600 RPM, 4 g PP, 0.4 g Ru_1-ZrO_2.

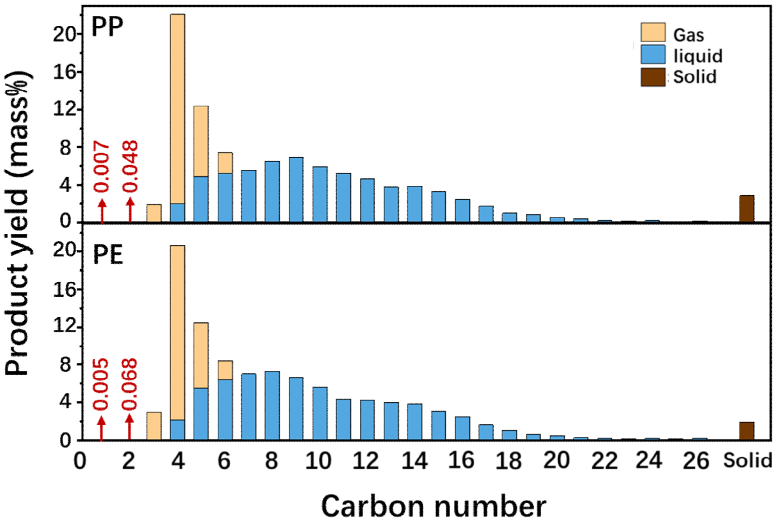

Supplementary Figure 1. Hydrocarbon distribution based on carbon number after hydrocracking of PP and PE over Ru_1-ZrO_2 at 250 °C under 3 MPa of H_2 for 8 hours. The gas and liquid products are labeled in orange and blue bars, respectively. The solid residue is shown in brown.

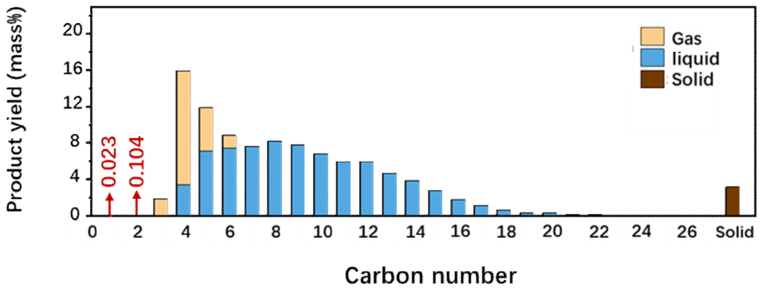

Supplementary Figure 4. Hydrocarbon distribution based on carbon number after hydrocracking of PP over Ru_1-ZrO_2 at 300 °C under 3 MPa of H_2 for 2 hours.

8- It is stated that a proof test over Ru1-ZrO2 under conditions favourable for methane production was conducted, but the conclusion from this test is missing.

Response:

We appreciate the opportunity to re-emphasize the conclusion from the proof test, which was indeed included in the manuscript on **Page 7**. We showed it below again for your reference.

"However, Ru_1-ZrO_2 still keeps ***the methane yield below 0.2 wt%*** *while* ***maintaining liquid fuels (C5-C20) around 70 wt%****, even under the reaction condition most greatly favoring methane formation."*

9.- In general I wonder if the presented results have been repeated at least once. No error bars are shown. Especially the stronger decrease of pressure at higher operation pressure could be also due to slight non-tightness of the reactors instead of more formation of aromatics.

Response:

Thank you for raising the importance of the error bar. We checked the leakage prior to every reaction. No step will be moved forward unless the pressure can be held for an hour without a pressure drop. We showed in Question 12 that how good the capability of holding the pressure of our reactor is when there is no reaction happened. Additionally, all experiments were repeated at leat for three times, and the variation of H_2 pressure changes is within 0.1 MPa, confirming the reliability of the data. To ensure the authenticity of the data, we have added error bars to **Supplementary Figure 6** and shown them below for your convenience.

Changes made in yellow to the Supplementary information (Page 17)

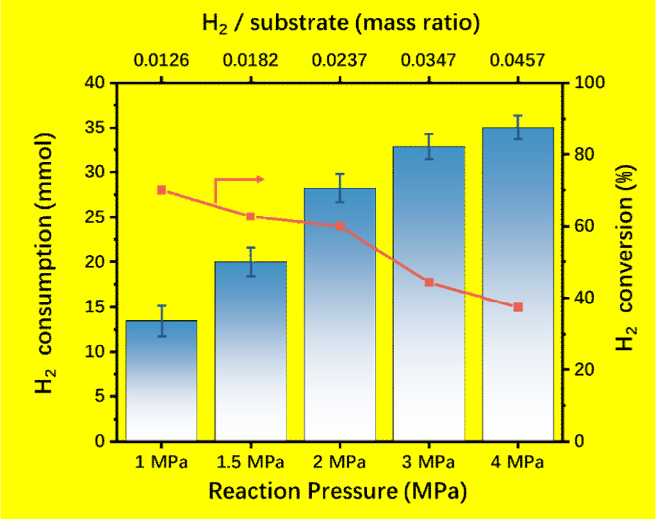

Supplementary Figure 6. Consumption and conversion of H_2 after 8 hours of hydrocracking PP over Ru_1-ZrO_2 at 300 ℃ under 1-4 MPa of H_2.

10- In the identification of the active site in the XRD where would reflexes of RuO2 be expected? What are the small reflexes at ~15 and 27° ?

Response:

Thank you for this insightful comment. We have added the XRD patterns of RuO_2 to the **Supplementary Figure 9 and 17.** According to the standard PDF cards, the XRD peaks at 28.1°, 35.1°, and 40.1° are assigned to RuO_2. Therefore, the small diffraction peaks at approximately 15° and 27° are not attributed to RuO_2, and they are also observed in pure ZrO_2. These small peaks are most likely attributed to the monoclinic phase of ZrO_2 (m-ZrO_2), which is also supported by the standard PDF card (PDF#97-006-8727).

Thank you again for your question, We have added the discussion and clarified the PDF card of m-ZrO_2 and RuO_2 in the revised Supplementary Information to ensure accurate interpretation of the XRD results.

Changes made in yellow to the Supplementary information (Page 20)

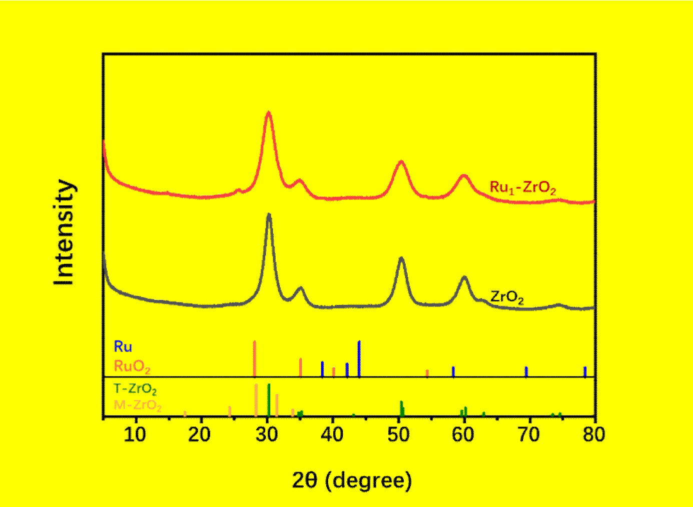

Supplementary Figure 9. XRD patterns of Ru_1-ZrO_2 and ZrO_2

The XRD patterns of Ru_1-ZrO_2 and ZrO_2 catalyst show the peaks of tetragonal ZrO_2 (T-ZrO_2, PDF#50-1089) at 30.27°, 34.81°, 50.38°, 60.20°, corresponding to (0 1 1), (0 0 2), (1 1 2), (1 2 0) crystal faces, respectively. In addition, a small diffraction peak is observed at ~25°, which is likely due to the monoclinic ZrO_2 (M-ZrO_2, PDF#97-006-8727). No characteristic diffraction peaks of hexagonal close-packed Ru (PDF#06-0663) and RuO_2 (PDF#97-001-5071) were observed, generally appearing at 44° and 28° . This indicates that Ru is highly dispersed on the ZrO_2 surface.

Changes made in yellow to the Supplementary information (Page 28)

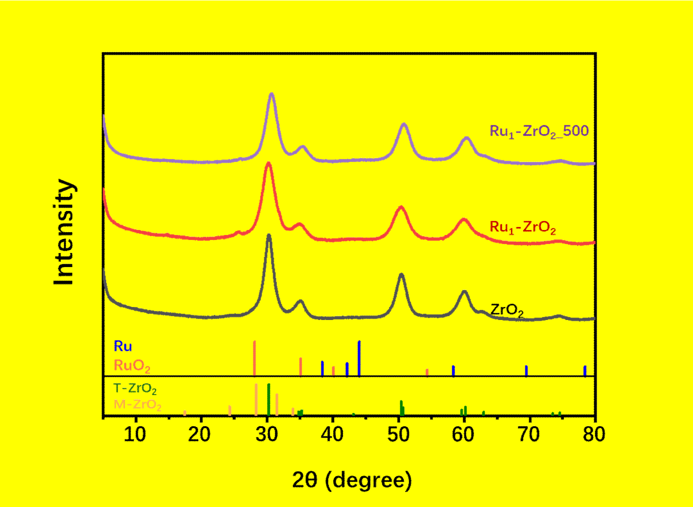

Supplementary Figure 17. XRD of Ru_1-ZrO_2_500, Ru_1-ZrO_2 and ZrO_2.

11- In general all provides analytics test the catalyst before the catalytic reaction. I wonder if the atomic dispersion of Ru maintains after the catalyst run and wonder how the surface properties change during catalysis. Especially 40MPa at 300° C is much harsher to the catalyst than the provided TPR under atmospheric pressure. A proof that the catalyst withstands full reduction under operation conditions is needed. In general I suggest to support the results with characterization of the catalyst after the catalytic runs.

Response:

Thank you for this important question. To evaluate the stability of Ru_1-ZrO_2, we performed post-reaction characterizations using HRTEM, EDS mapping, and XAS. As shown in **Supplementary Figure 29** after the reaction at 4 MPa and 300 °C for 8 hours, Ru remains highly dispersed, with no noticeable aggregation compared to its pre-reaction state. Furthermore, EXAFS (**Supplementary Figure 30(a)**) and XANES (**Supplementary Figure 30(b)**) analyses further confirm that Ru retains a single-atom dispersion state on the ZrO_2 after the reaction. In the manuscript, we also mentioned that after six cycles of experiments, the catalyst's activity remained unchanged, indicating that the surface properties of the catalyst remained.

We have added these results to the revised manuscript and Supplementary information as follows:

Changes made in yellow to the Manuscript (Page 14)

The robustness of Ru_1-ZrO_2 was assessed by the cycling experiment. No decrease in liquid yield, which is maintained at around 70 wt%, was observed after 6 cycles (Fig. 4a). HRTEM and EDS mapping (**Supplementary Figure 29)** revealed Ru species do not undergo aggregation after the reaction. Furthermore, EXAFS and XANES (**Supplementary Figure 30)** confirmed the catalyst's exceptional stability, with Ru remaining embedded as an atomic dopant within the ZrO_2 lattice after the reaction.

Changes made in yellow to the Supplementary Information (Page 40)

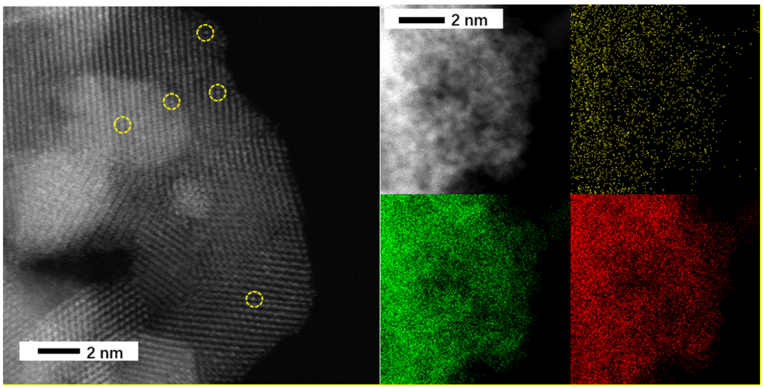

Supplementary Figure 29. HRTEM and EDS element mapping images of Ru_1-ZrO_2-used after the hydrocracking of PP at 300 °C under 3 MPa of H_2 for 8 hours.

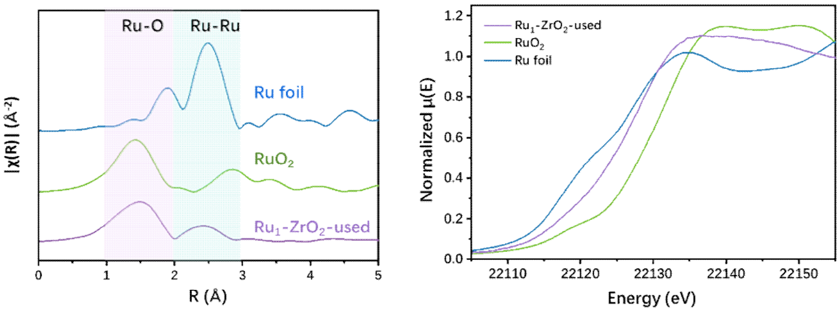

Supplementary Figure 30. Fourier transform of Ru EXAFS and Ru K-edge XANES spectra of Ru_1-ZrO_2-used after the hydrocracking of PP at 300 °C under 3 MPa of H_2 for 8 hours.

12- A test of Ru_1-ZrO_2_500 loss of PP activity is described, but only proven by a picture of the material after reaction. This is not a strong evidence and conversion rates should be provided.

Response:

Thank you for raising this excellent point. To further illustrate that Ru_1-ZrO_2_500 had completely lost its activity, we added a figure of the pressure as a function of reaction time during the reaction as **Supplementary Figure 16.** The results show that the pressure remains unchanged during the reaction when Ru_1-ZrO_2-500 is used as the catalyst. This indicates that there is no reaction happened. On the other hand, we further performed a detailed mass balance to confirm this. During the reaction, 4.0169 g of substrate was added, and 3.9604 g of unreacted solid was collected after the reaction. The minor discrepancy is attributed to unavoidable losses during product collection, as the plastic undergoes melting and re-solidification, some of them stick on the stirring paddles and walls of the reactor. Therefore, two more evidence confirmed that the catalyst has entirely lost its activity.

We have added these two pieces of evidence and relevant discussion to the manuscript and Supplementary Information as follows:

Changes made in yellow to the Manuscript (Page 9):

We found that in Ru_1-ZrO_2_500, the disappearance of the Ru-O-Zr reduction signal in the H_2-TPR profile (**Fig. 2f**) is accompanied by the loss of activity in PP hydrocracking, as demonstrated by the weight-balanced post-reaction solid residue (**Supplementary Fig. 15**) and the unchanged pressure during the reaction (**Supplementary Fig. 16**).

Changes made in yellow to the Supplementary Information (Page 27)

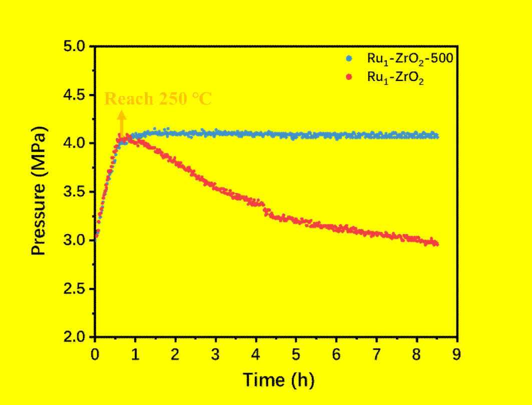

Supplementary Figure 16. Pressure variation during the reaction of hydrocracking PP over Ru_1-ZrO_2 and Ru_1-ZrO_2-500 at 250 °C under 3 MPa H_2 for 8h.

13.- EDX of Ru_1-ZrO_2: how long was the EDX collected? The Ru detection looks like a typical scattering image what would be obtained even if no Ru is present.

Response:

Thank you for raising this question. Exactly, similar EDS mapping results could be obtained even without Ru, and we understand those are noise, so we did not claim that the random bright dots are attributed to the Ru atoms. Instead, we just used the EDS mapping to demonstrate the absence of Ru clusters, as a 2-minute acquisition time is long enough to see accumulated centered dots reflected by Ru clusters or nanoparticles. Moreover, we acknowledge that EDS resolution is limited and prone to noise, so we cannot use it to characterize the Ru single atoms. Therefore, we performed further EXAFS experiments to study the status of Ru. What is concluded using EDS is presented here again, which was shown in Page 7:

"The particle size of Ru_1-ZrO_2 is around 5 nm (Fig. 2a), and energy-dispersive X-ray spectroscopy (EDS) element mappings confirm that the ***absence* of *Ru clusters*** *in the catalyst (Fig. 2b)"*

14- The XANES data show no full reduction of Ru. What is the oxidation state of Ru? To what fraction is the Ru in what oxidation state? LCF or similar should be performed.

Response:

Thank you for this insightful question. Actually, we discussed the oxidation state of Ru in the Ru_1-ZrO_2 catalyst based on the characterization of XPS, which was originally shown in Supplementary Figure 21a. The XPS results for Ru_1-ZrO_2 reveal that Ru predominantly exists in the Ru^{4+} (84.4%) and Ru^{3+} (15.5%) oxidation states, with no significant presence of metallic Ru^0. This indicates that Ru is stabilized in an oxidized state within the ZrO_2 lattice. These findings are also consistent with XANES data. In contrast, the XPS analysis of Ru_1-ZrO_2-500 shows that approximately half of the Ru species have been reduced to metallic Ru^0 following high-temperature reduction at 500 °C. We showed the figure below again for your convenience.

We added more discussions about the status of Ru to the Supplementary Information as follows:

Changes made to the Supplementary Information in yellow (Page 32):

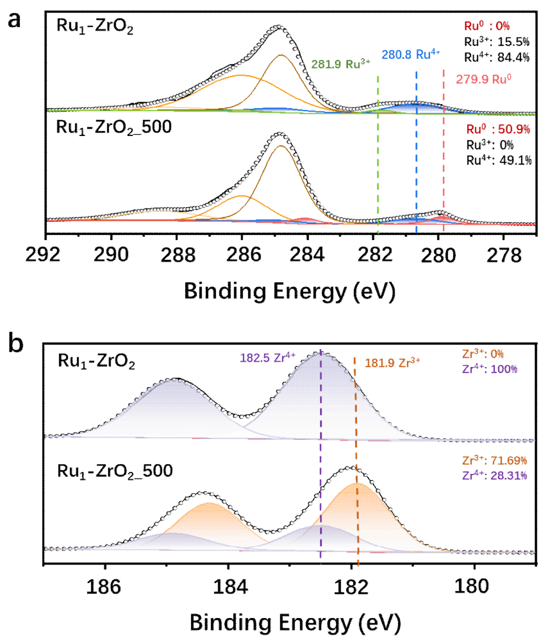

Supplementary Figure 21. (a) Ru 3*d* and **(b)** Zr 3*d* XPS spectra of Ru_1-ZrO_2_500 and Ru_1-ZrO_2.

The XPS results show that Ru exists predominantly in the Ru^{4+}(84.4%) and Ru^{3+}(15.5%) oxidation states in Ru_1-ZrO_2, with no significant presence of metallic Ru^0. These findings align with the XANES and confirm that Ru remains in an oxidized state. However, in Ru_1-ZrO_2-500, approximately half of the Ru species were reduced to Ru^0. The Zr 3*d* XPS peak around 182 eV exhibits two contributions, Zr^{4+} 3*d* 5/2 and Zr^{3+} 3*d* 5/2, located at respectively 182.5 eV and 181.9 eV.[35,36] It is observed that the contribution of Zr^{3+} in Ru_1-ZrO_2_500 is much more significant than that in Ru_1-ZrO_2, indicating a reduction from Zr^{4+} to Zr^{3+} after the H_2 treatment on Ru_1-ZrO_2.

15- Why is the performance of Ru_1-ZrO_2-500 so much different to Ru/ZrO_2?

Response:

Thank you for this question. Ru_1-ZrO_2-500 refers to the catalyst obtained by reducing Ru_1-ZrO_2 in a 20% H_2/Ar atmosphere at 500 °C for 2 hours. In contrast, Ru/ZrO_2 is prepared by impregnating a commercial ZrO_2 support with Ru precursor solution using an incipient wetness impregnation method.

Regarding their performance in hydrocracking PO, there are no differences, neither of them shows any activity.

Regarding their performance in the H_2-TPR (Fig. 2f), Ru/ZrO_2 exhibits a reduction peak at around 180 °C corresponding to Ru clusters (**Supplementary Figure 12a**). However, Ru_1-ZrO_2-500, having already undergone high-temperature reduction, does not display the peak.

Regarding their performance in the H_2-D_2 exchange experiment (Fig. 2g), Ru_1-ZrO_2-500 demonstrates a stronger ability to activate H_2 than Ru/ZrO_2. Because Ru_1-ZrO_2-500 retains the original high dispersion of Ru, its performance is similar to that of Ru_1-ZrO_2. In contrast, Ru/ZrO_2, which was synthesized via impregnation, has larger particles with poorer dispersion, resulting in a smaller HD area. This difference in particle size and dispersion has already been discussed in the manuscript. For your convenience, we have placed the mentioned data below. We hope this explanation addresses your concern.

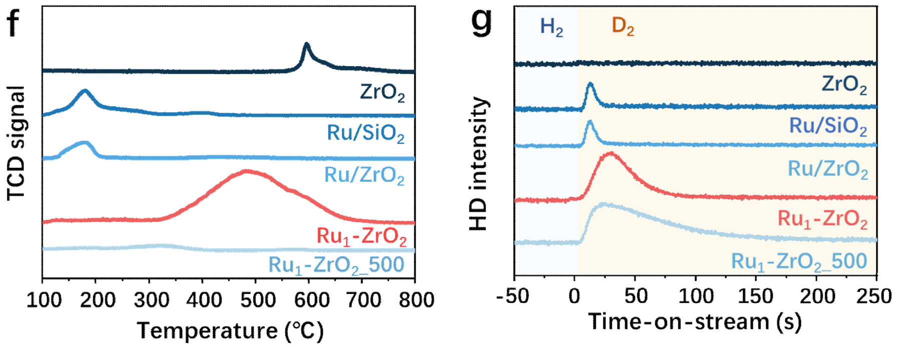

Fig 2. f H_2-TPR profiles of ZrO_2, Ru/SiO_2, Ru/ZrO_2, Ru_1-ZrO_2, and Ru_1-ZrO_2_500. These samples were not pretreated in H_2 at 275 °C to obtain their complete TPR profiles. **g,** H_2/D_2 switch experiment tracking m/z=3 (HD) by mass spectrometer: 10% H_2/Ar flow switched to 10% D_2/Ar flow with a constant rate of 20 mL/min at 250 °C at the time of 0 s.

16-Where the DFT calculation really done with PP? Or war as any model compound used?

Response:

Thank you very much for your reminder. In the DFT calculations, an alkane of C11 with the branched methyl groups was used as a model compound. We showed the structure of the compound in **Supplementary Figure 28,** but forgot to mention it in the Methods. We have added the name of the model compound to the Methods of the Manuscript.

Changes made to the manuscript in yellow on Page 21 :

The alkane of C11 with the branched methyl groups was used as a model compound. The adsorption energy of the reaction intermediate was calculated as $E_{ads} = E_{adsorbate+surface} - E_{adsorbate} - E_{clean-surface}$.

17- The Mechanism in Figure 3a provide details for the formation of C3, C5, and C6 compounds. But the average from the catalytic resulst is C8. How can the tendency for C8 be explained?

Response:

Thank you for your insightful question. The purpose of Fig. 3a (re-shown below for your convenience) is to explain why no methane is observed in the gas. In the case of the C-C bond breaking near the end of the long chain, a small gaseous molecule forms meanwhile a long-chain molecule forms as well. If the C-C bond breaking happens in the middle of the chain, it results in two long-chain molecules (This case was depicted in **Fig. 3b**). Since the number of carbons in the middle of the chain far exceeds those at the ends, most carbonium ions are formed in the middle, leading to liquid products as the dominant product. In summary, we aim to use the gas production scenario to illustrate why methane formation is effectively suppressed, so we showed the case of the C-C bond breaking near the end of the chain.

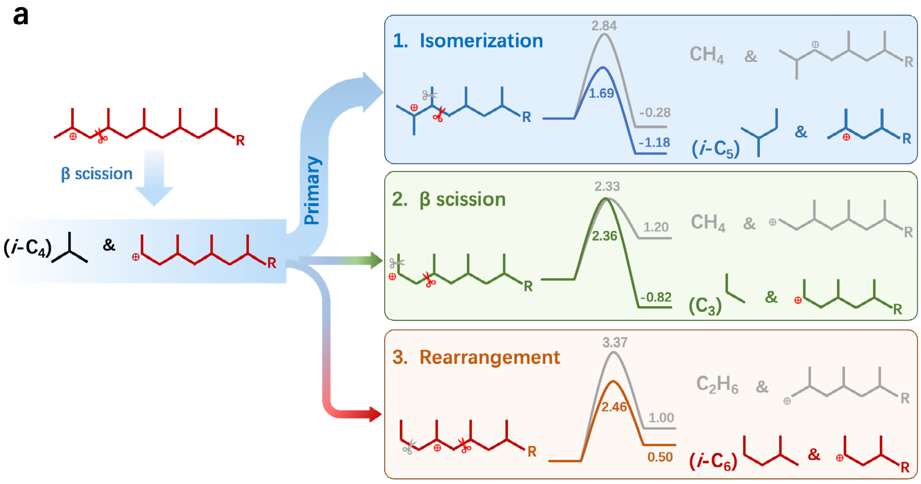

Fig. 3a, Reaction pathways and DFT calculated energy profiles of PP hydrocracking to gas products via β-scission mechanism with carbonium ions (energy unit: eV). The molecule structures in red or blue color are the same type of carbonium circularizing in the hydrocracking loop.

18- Regarding the general Mechanism of olefin hydrotreating a lot of literature is available (especially focusing hydrotreating of Fischer-Tropsch) which is reaction wise very similar to Polyolefin-Hydrotreating. These literature should be referred to. Given that knowledge the provided mechanism is not very new. Additionally since no operando surface analytics such as DRIFTS or Raman is provided the given mechanism is faily speculative. Within the manuscript no direct proof for the adsorption of the carbonium ion on the O atom is given. If no surface characterization is available, kinetic investigations together with microkinetic modelling could help to clarify and support the proposed reaction mechanism.

Response:

Thank you for your insightful comment. The hydrogenation conversion of polyolefins is similar to the hydrocracking of alkanes/olefins, as described in several literature (Journal of Catalysis. 1998,174, 177–184; Ind. Eng. Chem. Res. 1996, 35, 386–396; Journal of Catalysis. 2004, 221, 241–251). To further support our understanding

of the reaction mechanism, we have added those literature as references 44 - 46. We showed it below for your convenience.

Due to the high temperature, high pressure, and the gas-liquid-solid three-phase involved in this reaction, it is very challenging to design or find an operando cell to accommodate these harsh reaction conditions for spectroscopy. Similar to the kinetics study, this reaction is conducted in an autoclave instead of the fixed-bed reactor, and it involves the interfaces of gas, liquid, and solid, it is extremely challenging to keep the reaction in a kinetic controlled regime and define a model for the three-phase interaction. However, we agree that operando spectroscopy and kinetics modeling are very useful for understanding the mechanism, and we are actively seeking collaborators who possess or can develop state-of-the-art high-pressure high-temperature spectroscopic techniques to address this challenge and provide further insights into the reaction mechanism.

Changes made to the manuscript in yellow on Page 12

During PP deconstruction, the formation of the more stable tertiary carbonium ion is favored over, primary and secondary carbonium ions[44-46].

Reference:

44. Maesen, T. L. M., Calero, S., Schenk, M. & Smit, B. Alkane hydrocracking: shape selectivity or kinetics? *J. Catal.* 221, 241-251, (2004).
45. Girgis, M. J. & Tsao, Y. P. Impact of catalyst metal acid balance in n-hexadecane hydroisomerization and hydrocracking. *Industrial & Engineering Chemistry Research* 35, 386-396, (1996).
46. Souverijns, W., Martens, J. A., Froment, G. F. & Jacobs, P. A. Hydrocracking of isoheptadecanes on Pt/H-ZSM-22: An example of pore mouth catalysis. *J. Catal.* 174, 177-184, (1998).
